# Supplementary figures and images for: Animal, Fungi, and Plant Genome Sequences Harbor Different Non-Canonical Splice Sites
Source: Cells. 2020 Feb 18;9(2):458. doi: 10.3390/cells9020458 (PMC7072748; doi:10.3390/cells9020458)

# animals

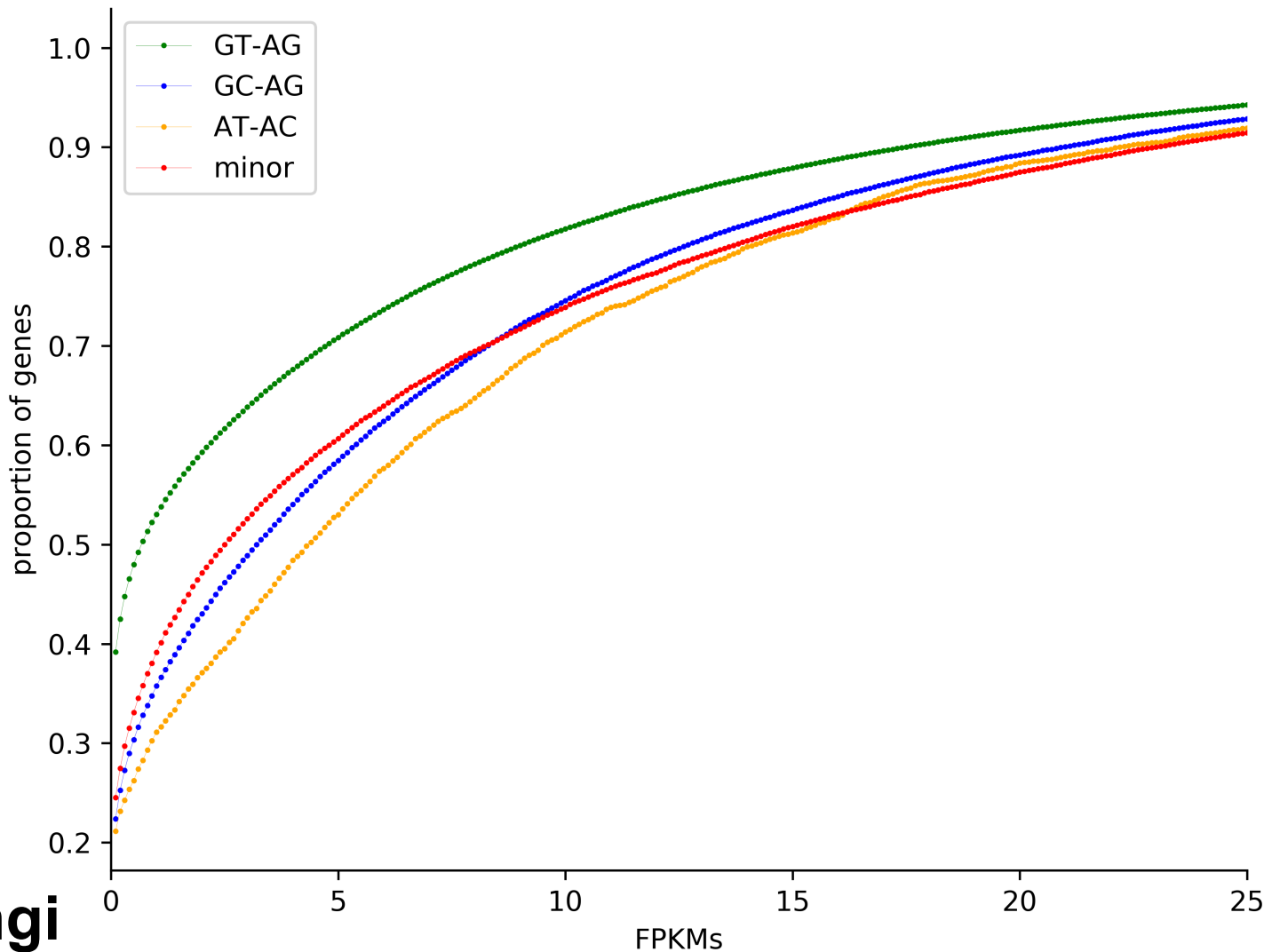

# fungi

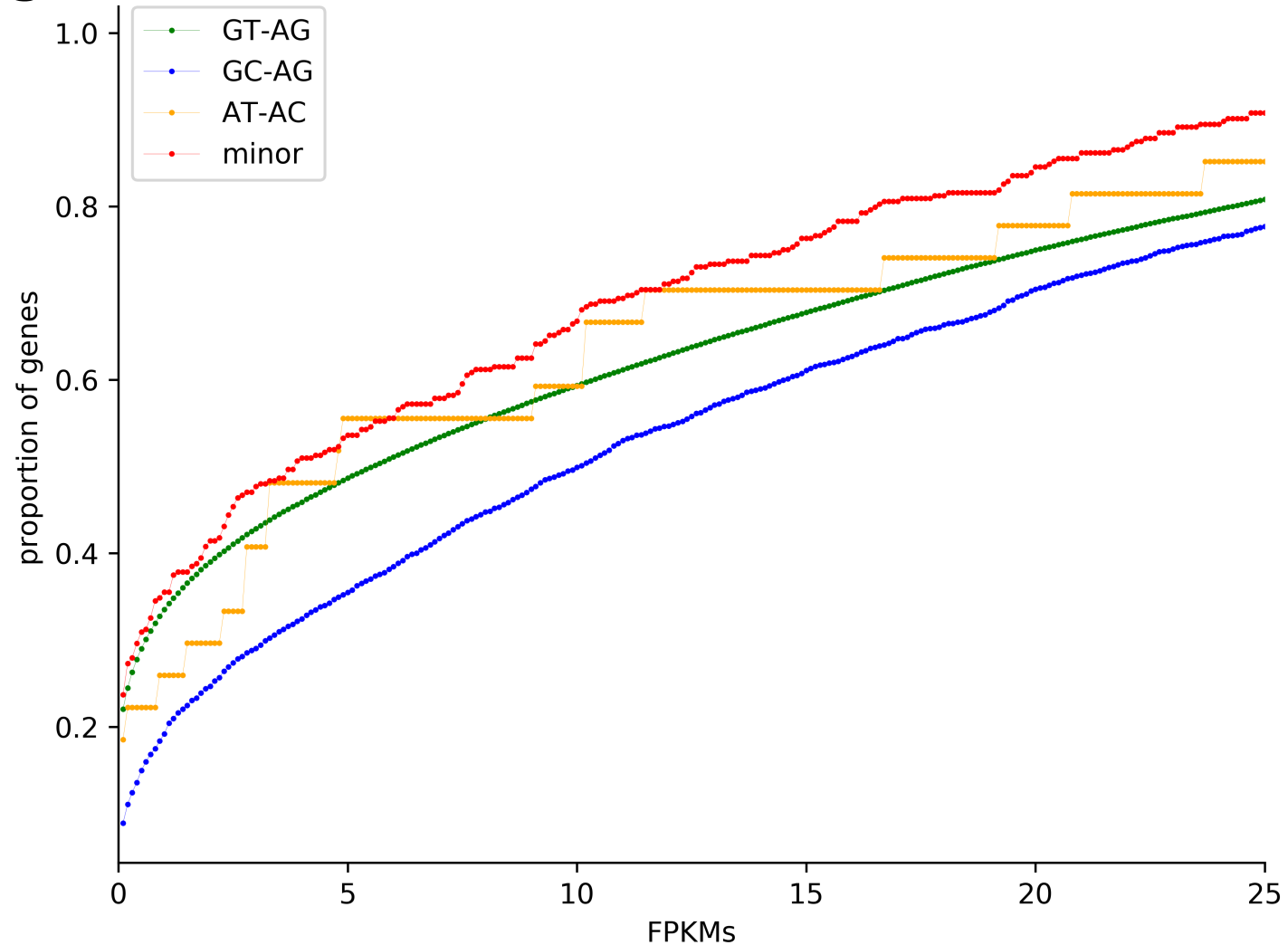

# animals

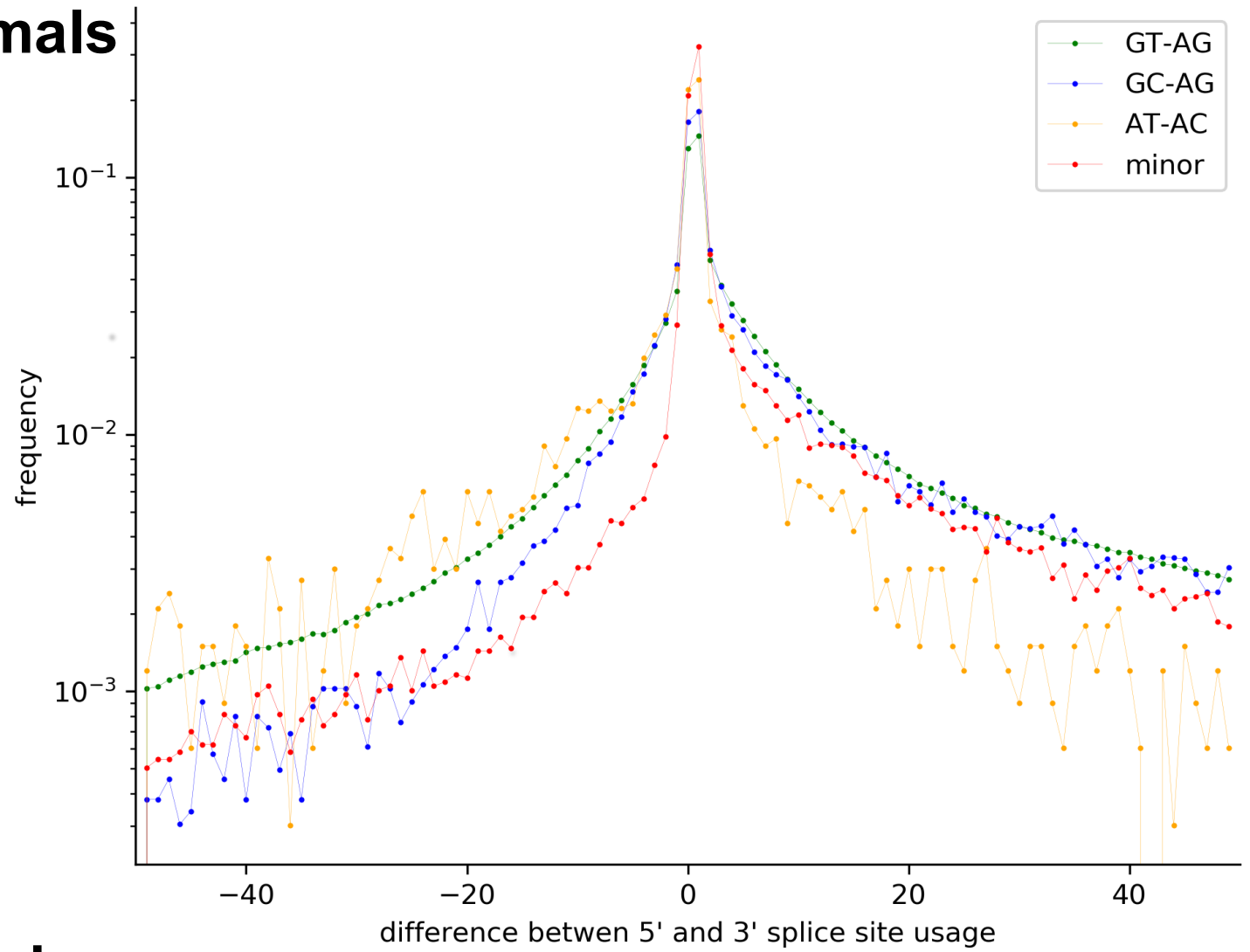

# fungi

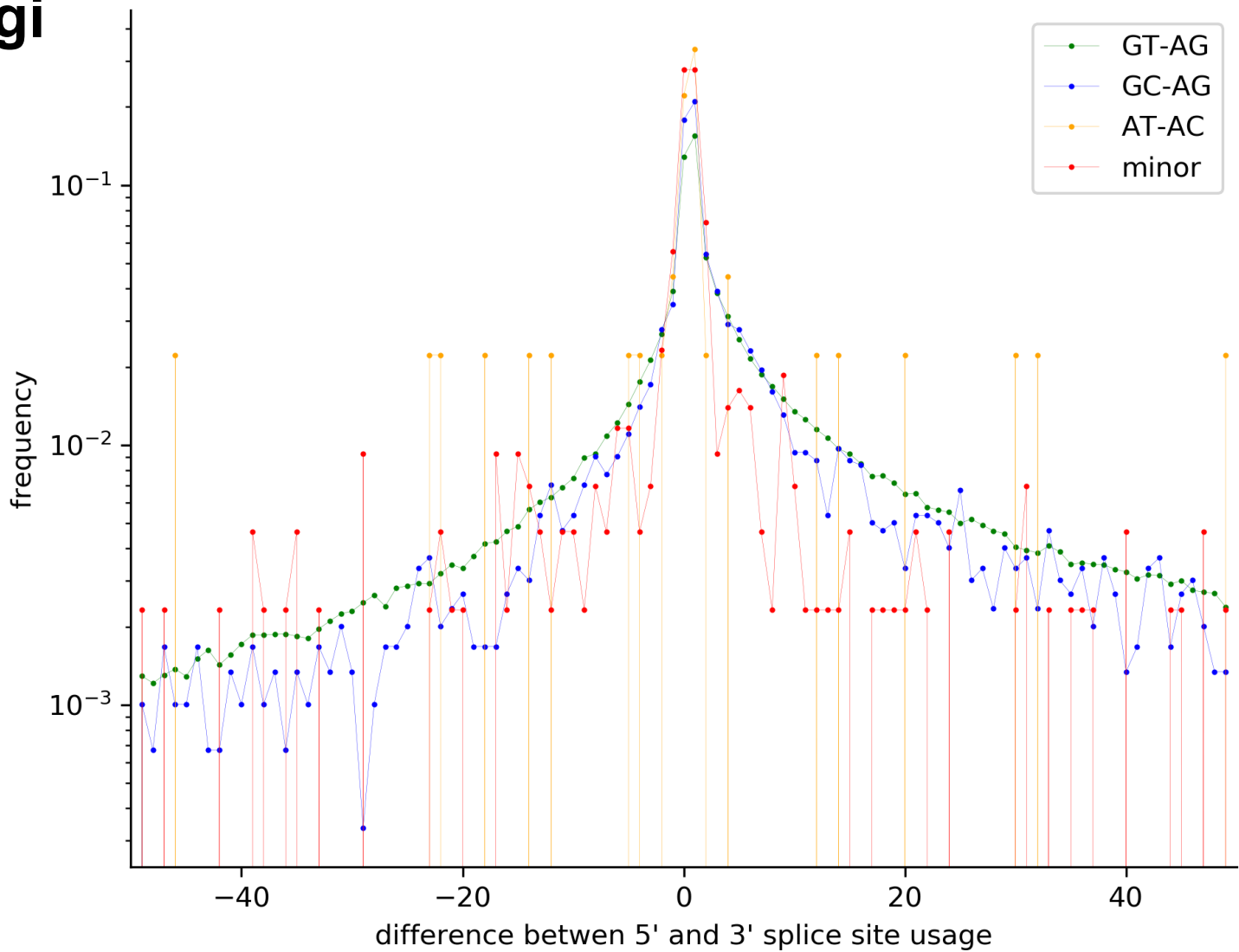

Supplement: Supplementary file 1 [file cells-09-00458-s001.zip › supplements/File S14.pdf]

# animals

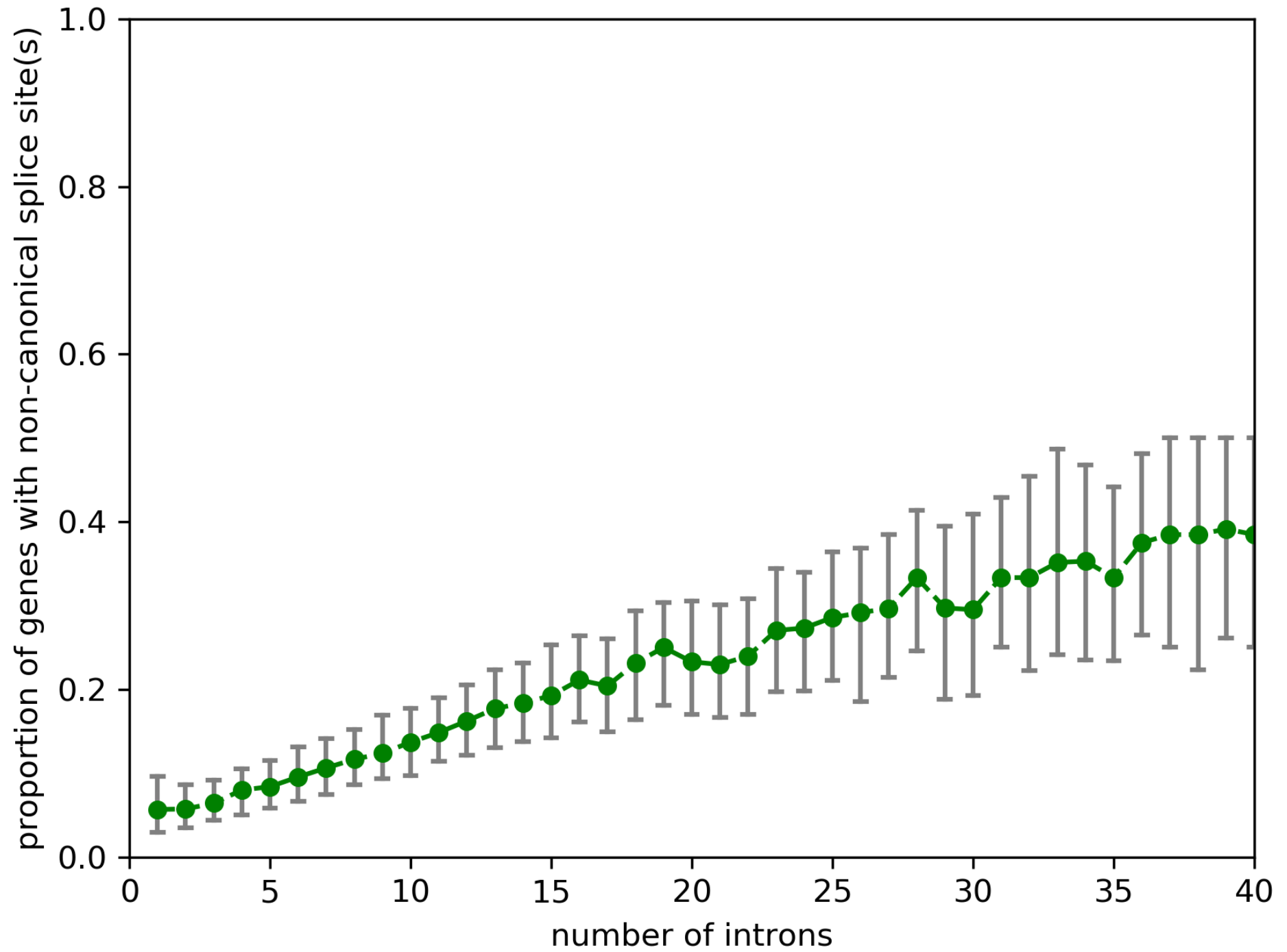

**fungi**

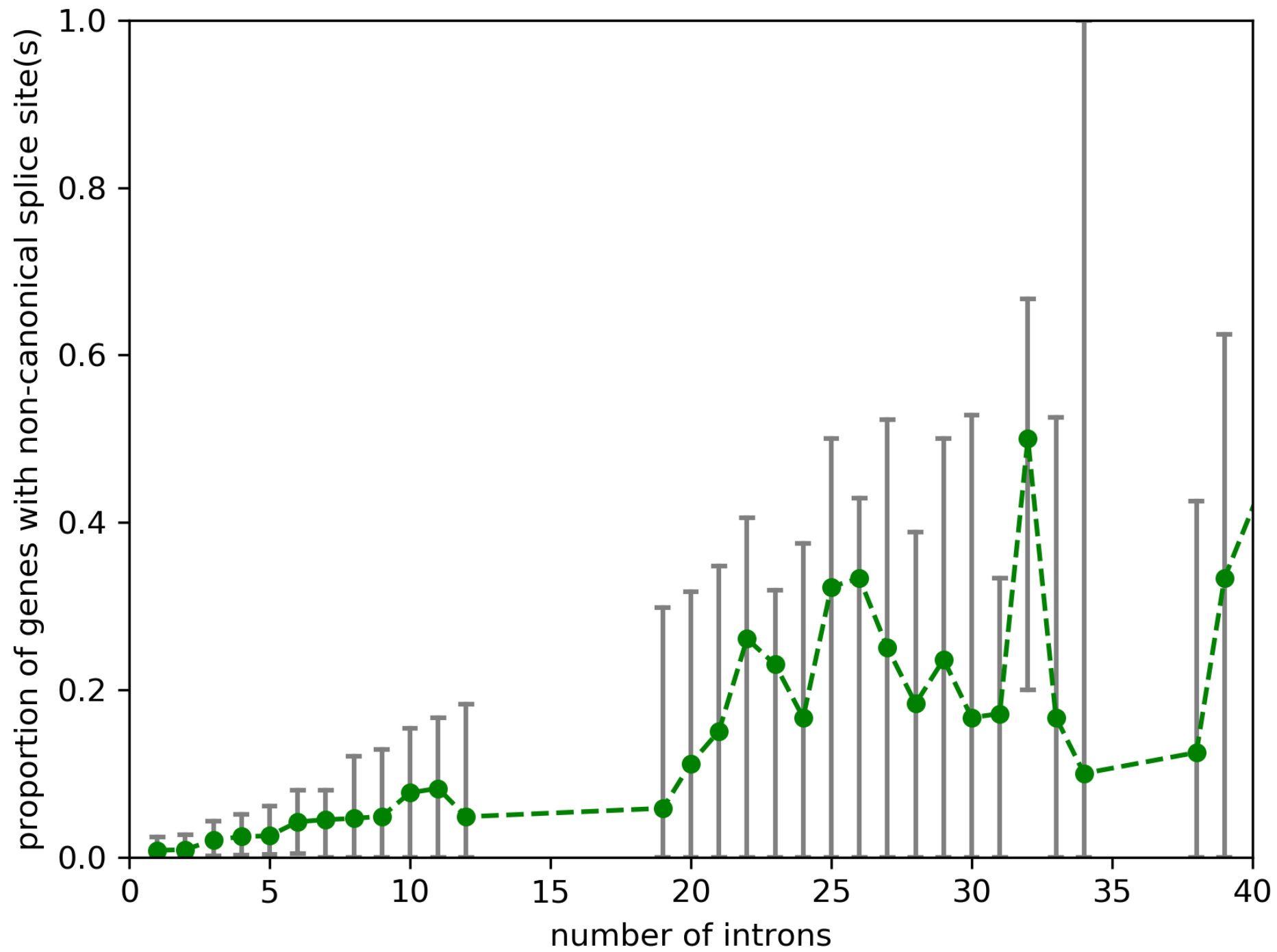

Supplement: Supplementary file 1 [file cells-09-00458-s001.zip › supplements/File S13.pdf]

animals

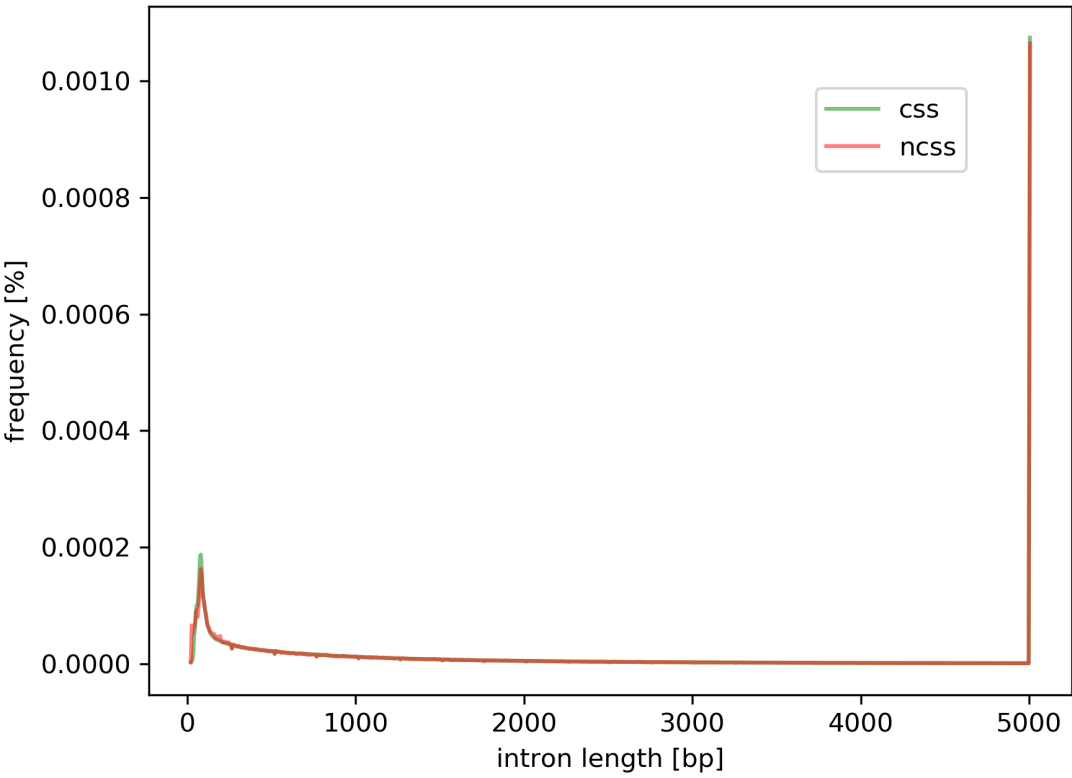

fungi

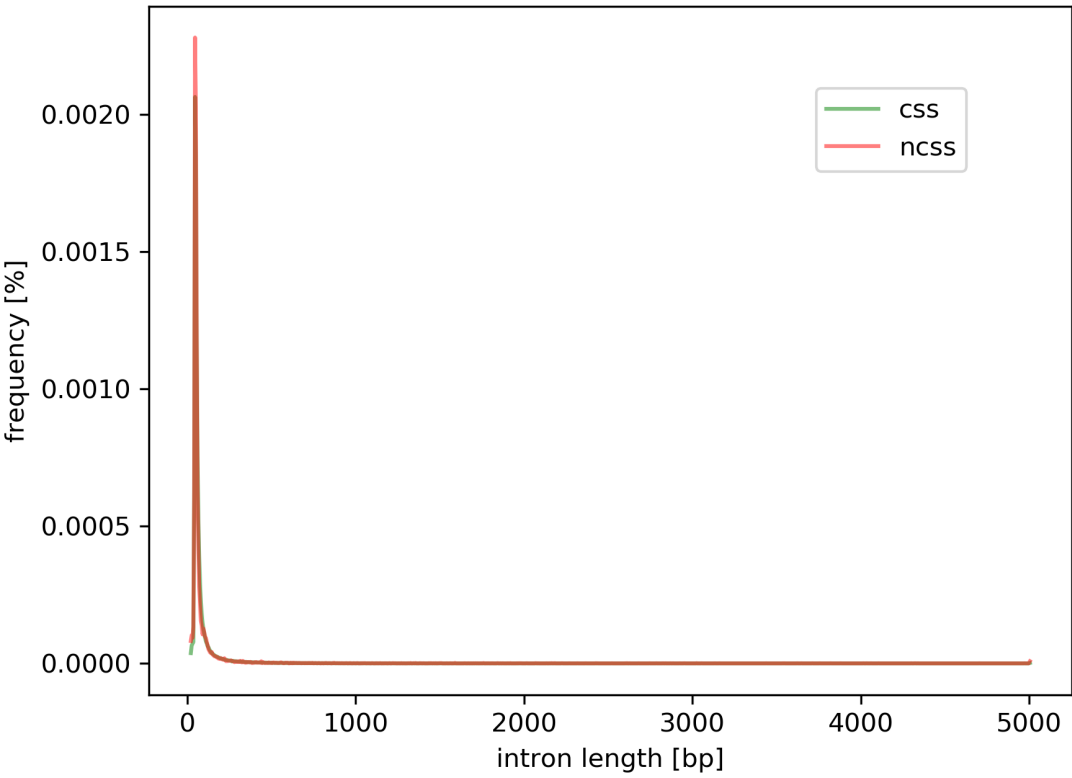

Supplement: Supplementary file 1 [file cells-09-00458-s001.zip › supplements/File S21.pdf]

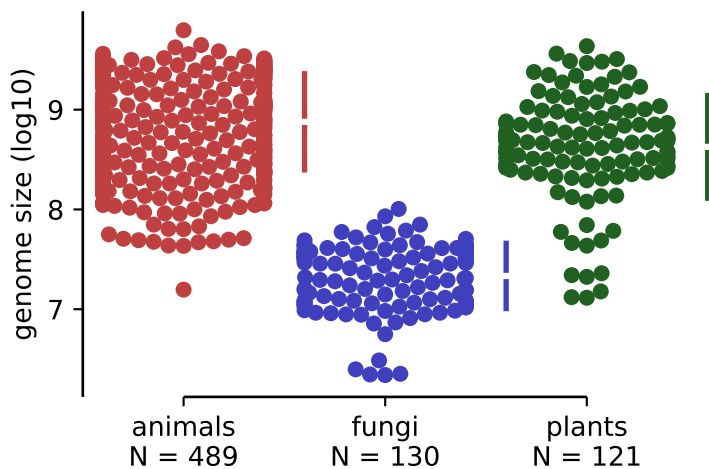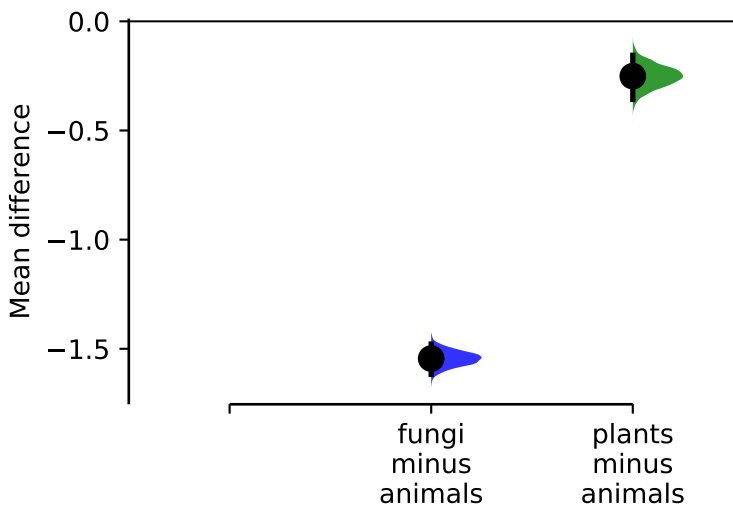

Supplement: Supplementary file 1 [file cells-09-00458-s001.zip › supplements/File S5.pdf]

# animals

$r = 0.43437813971655936$ ,  $p = 7.866123362747553e-24$

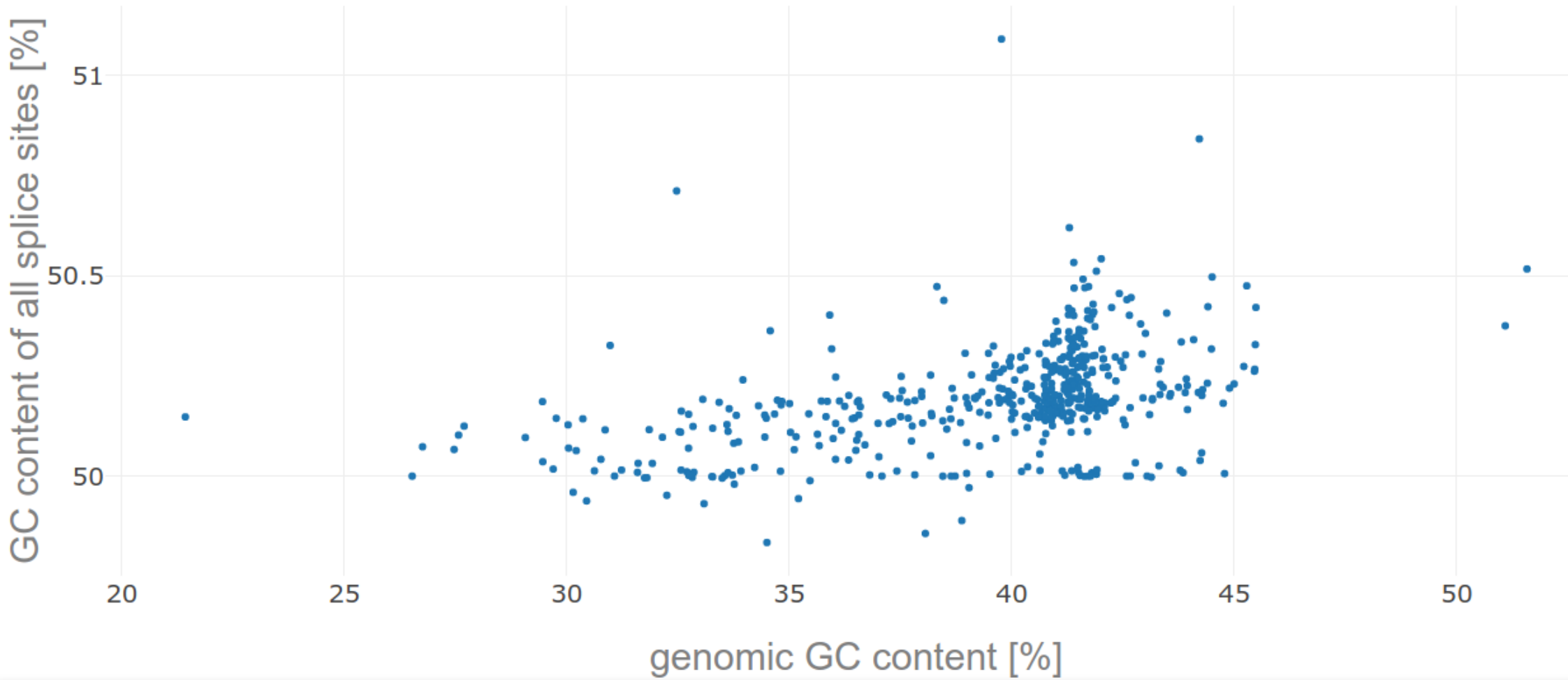

# fungi

$r = 0.23568093817494543$ ,  $p = 0.007891452637704533$

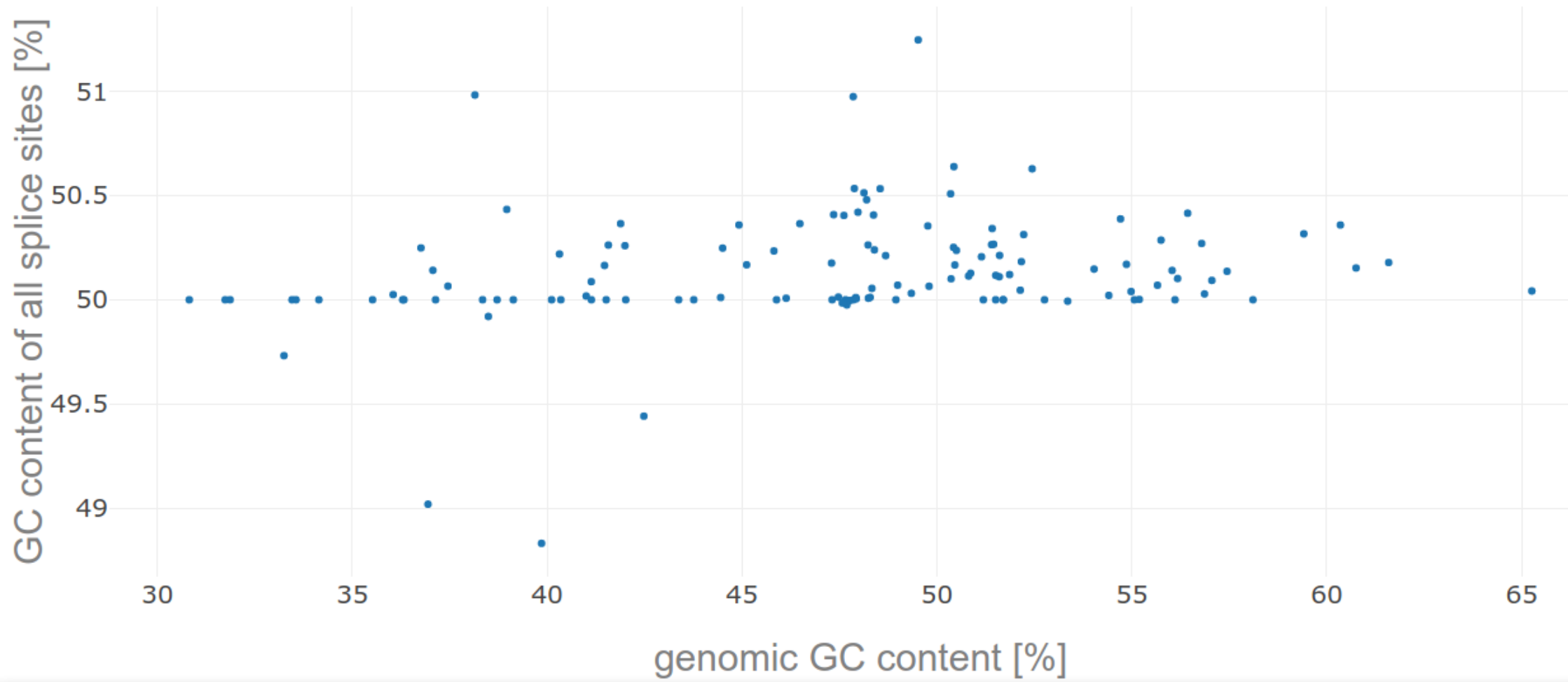

# plants

$r = 0.40330104697931257$ ,  $p = 4.504868690928474e-06$

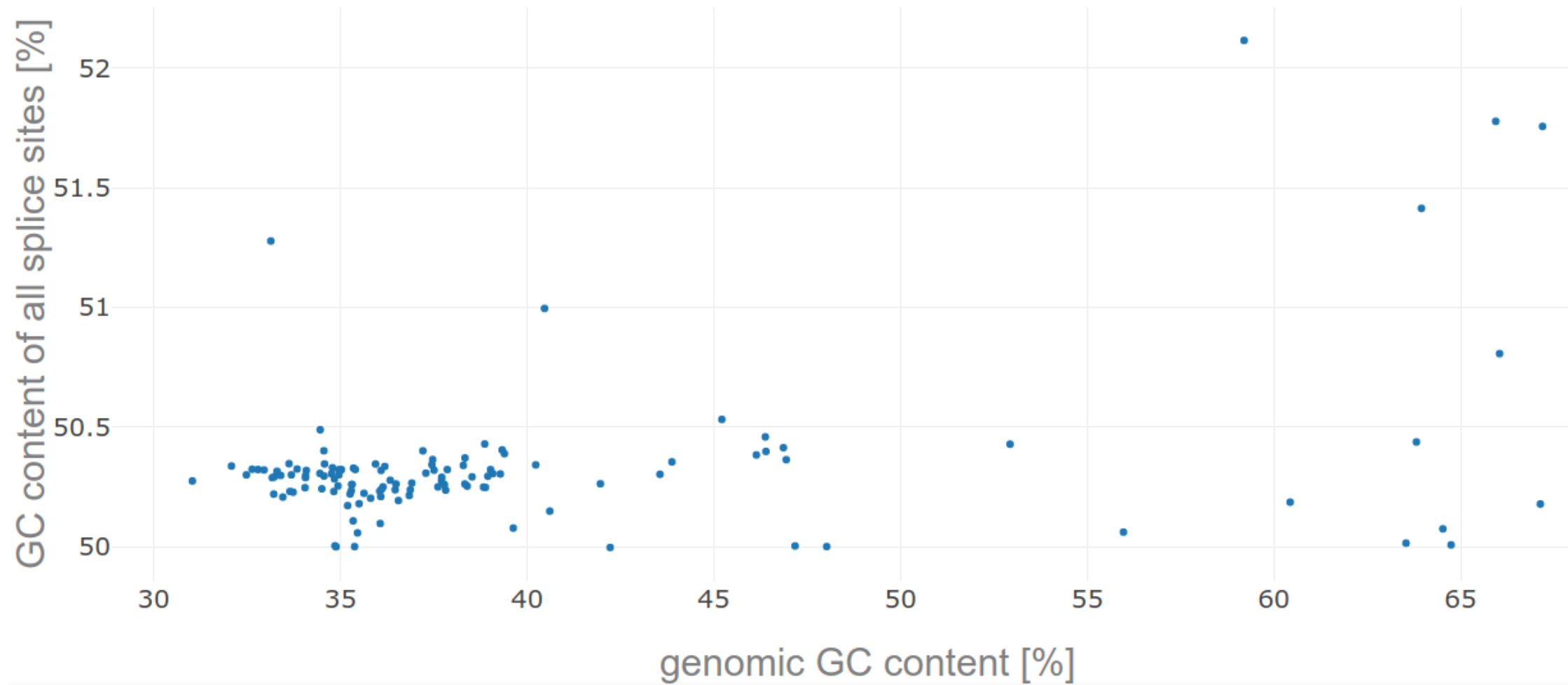

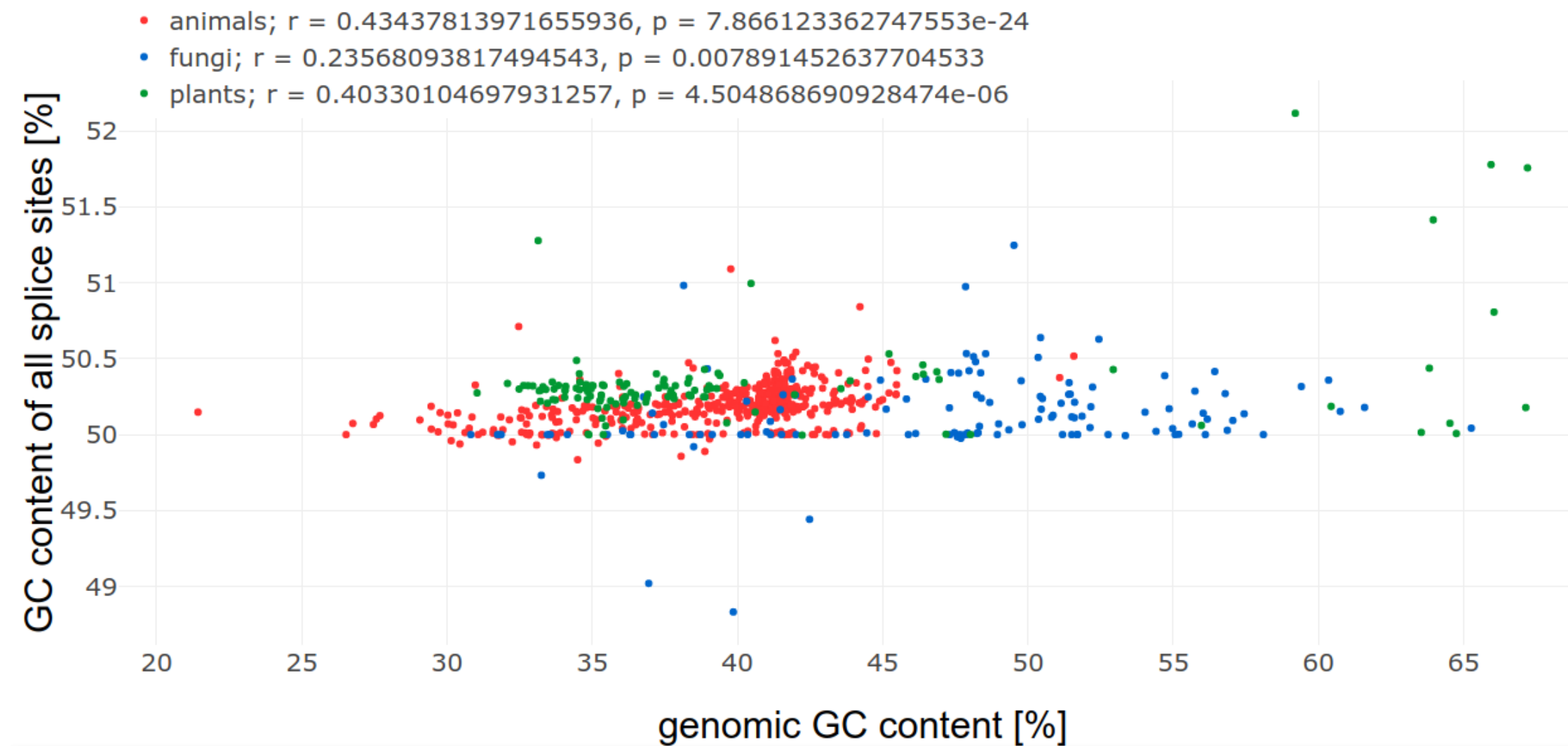

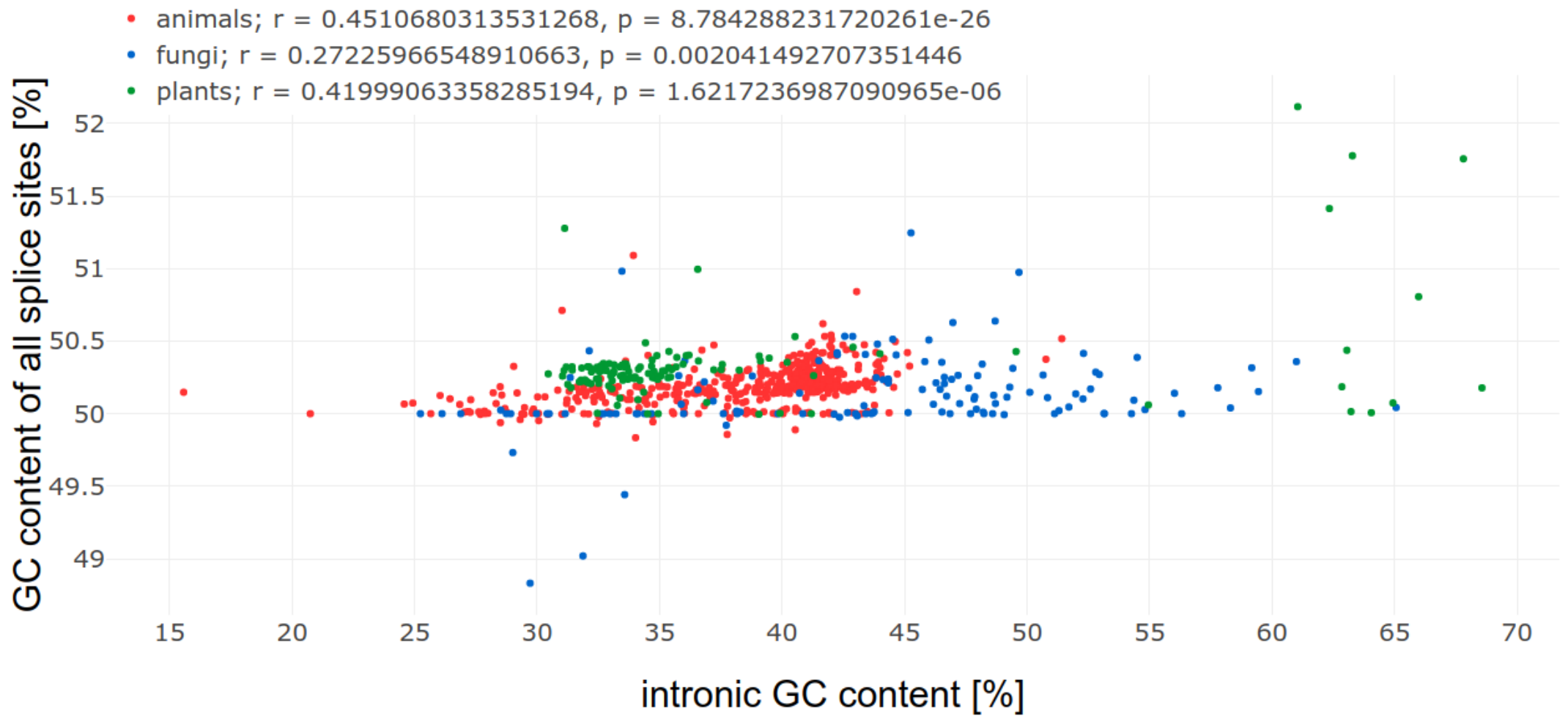

Supplement: Supplementary file 1 [file cells-09-00458-s001.zip › supplements/File S11.pdf]

animals

number of supported splice sites

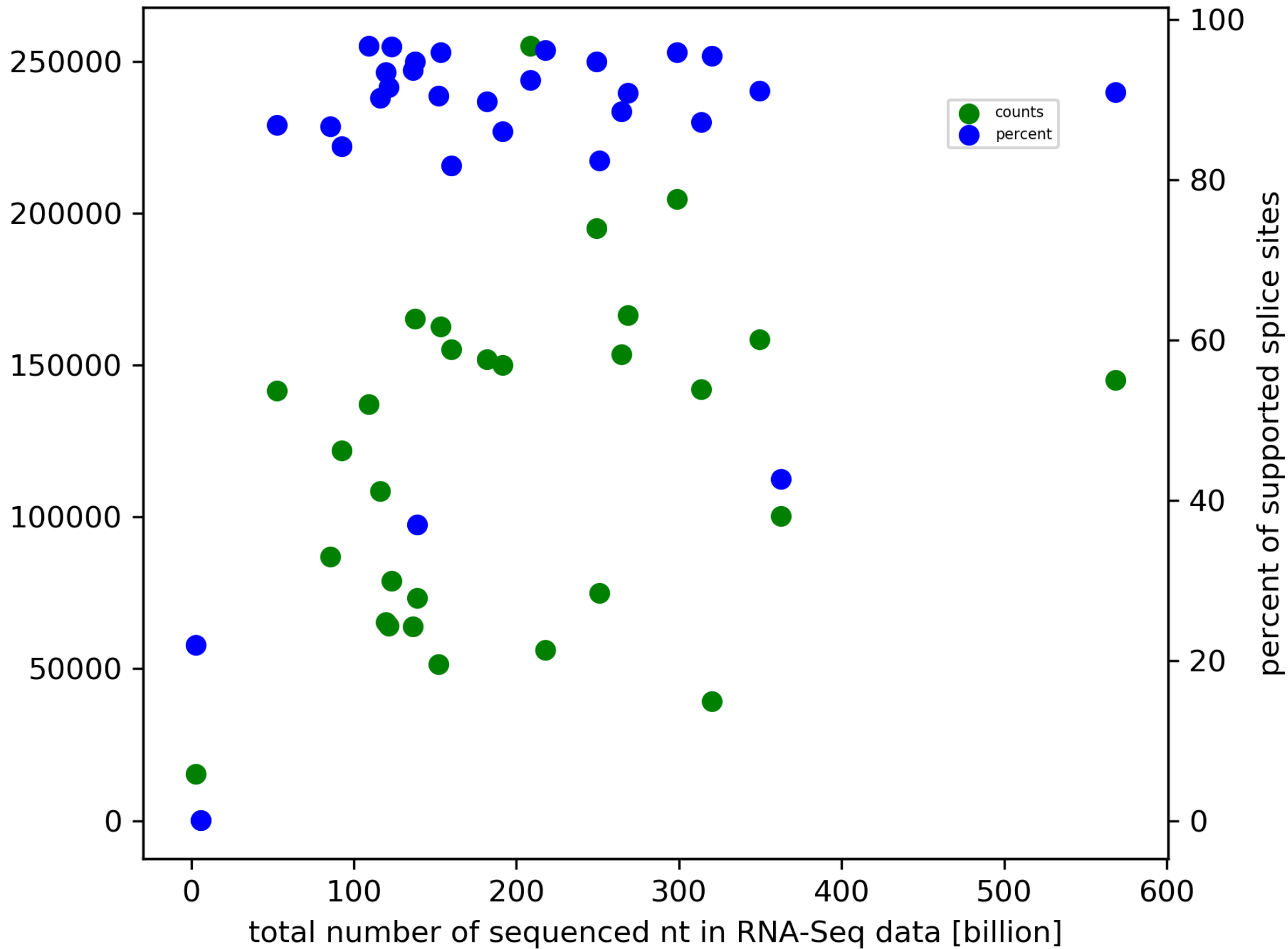

# animals

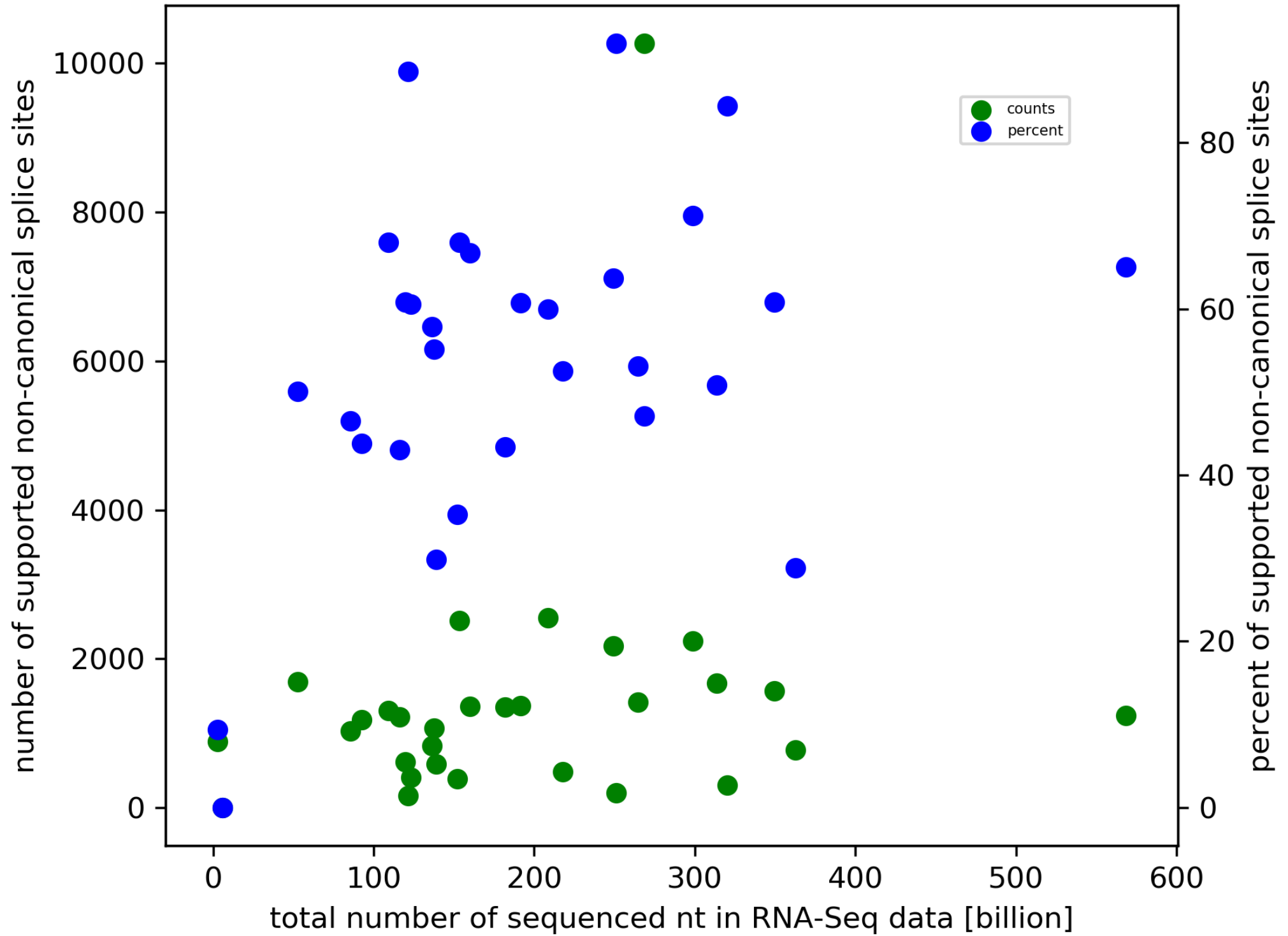

fungi

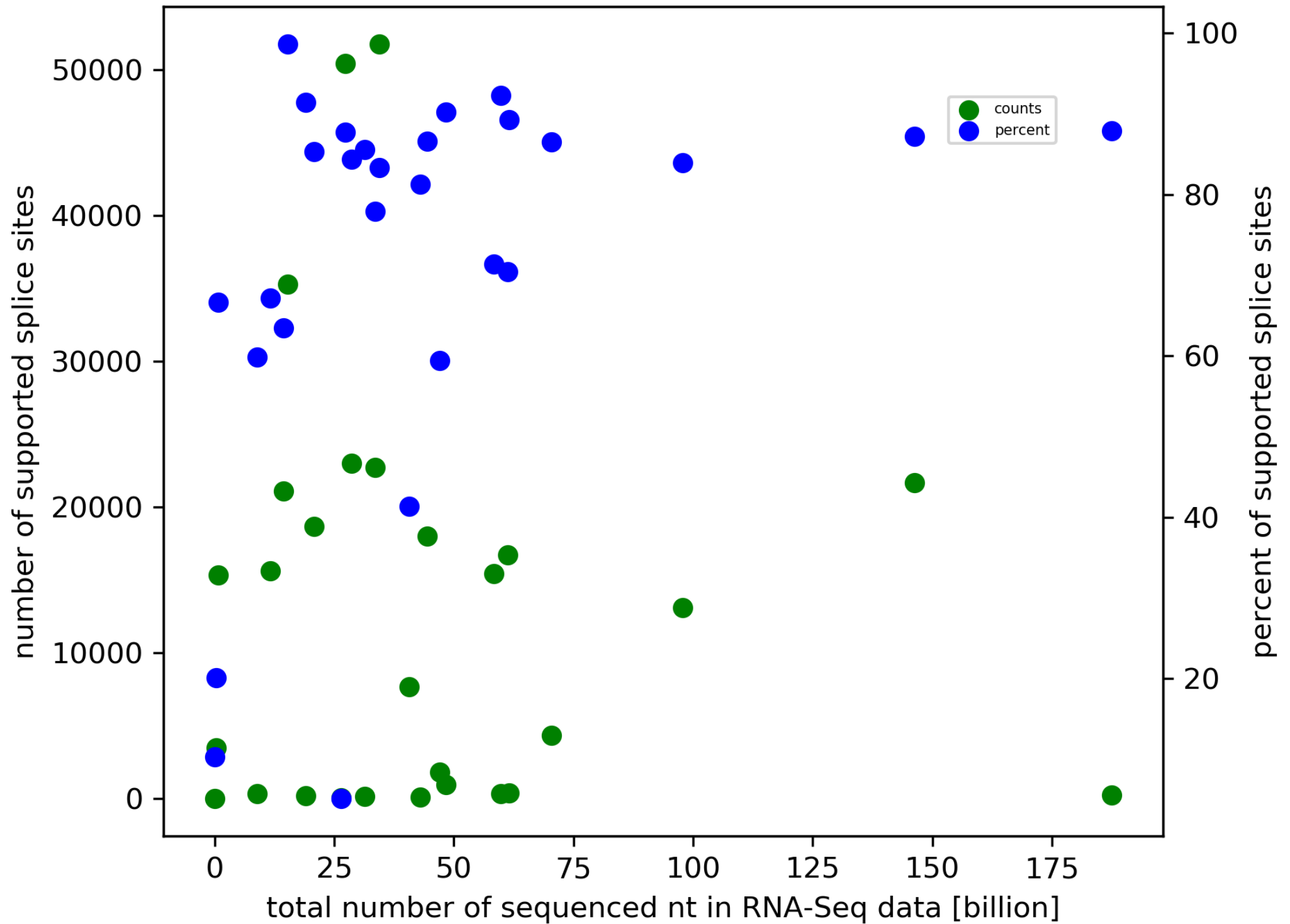

fungi

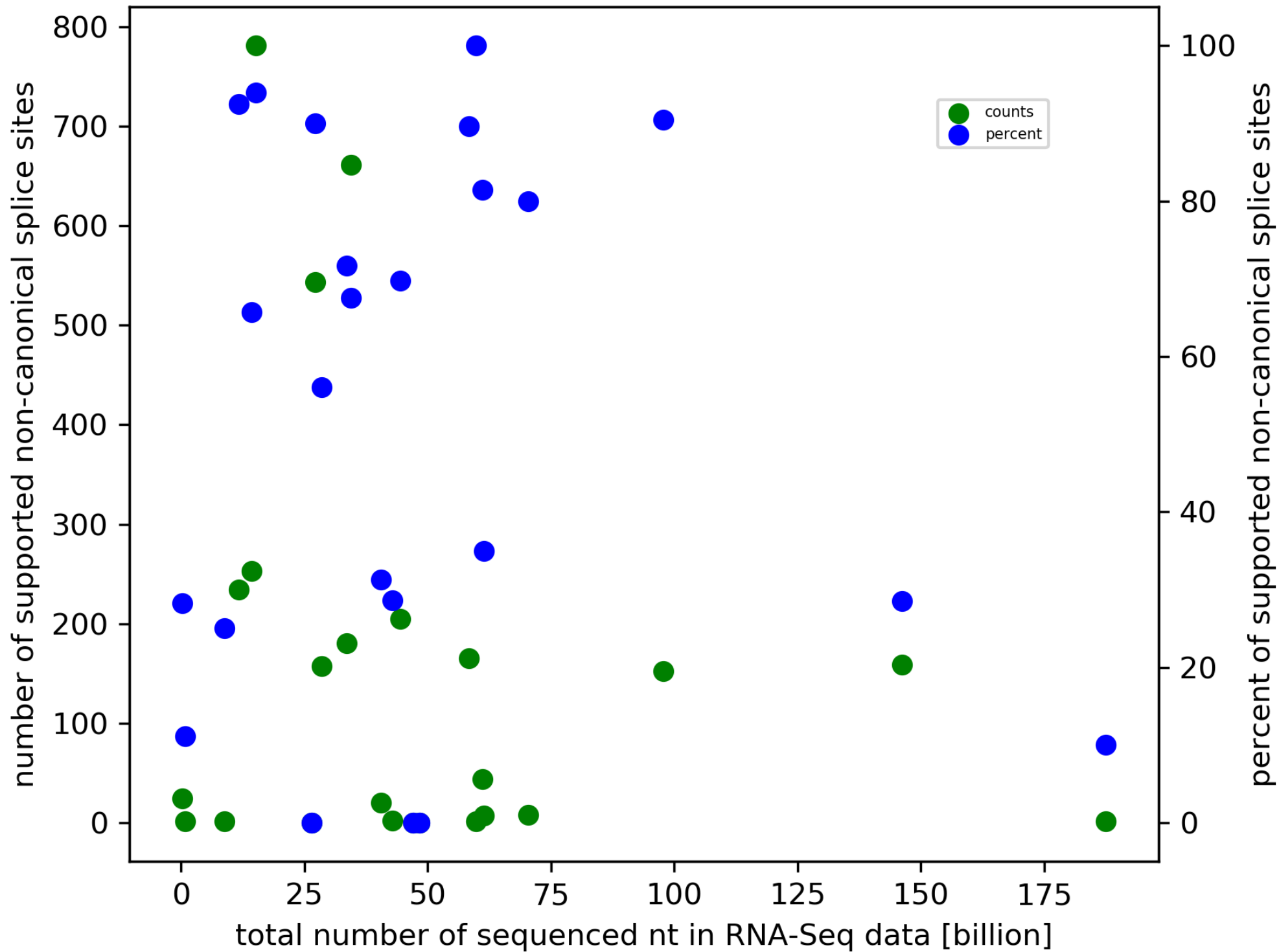

Supplement: Supplementary file 1 [file cells-09-00458-s001.zip › supplements/File S12.pdf]
