# Supplementary material for: Animal, Fungi, and Plant Genome Sequences Harbor Different Non-Canonical Splice Sites
Source: Cells. 2020 Feb 18;9(2):458. doi: 10.3390/cells9020458 (PMC7072748; doi:10.3390/cells9020458)
Supplement: Supplementary file 1 [file cells-09-00458-s001.zip › supplements/File S19.zip!/files_for_S19/892.pdf]

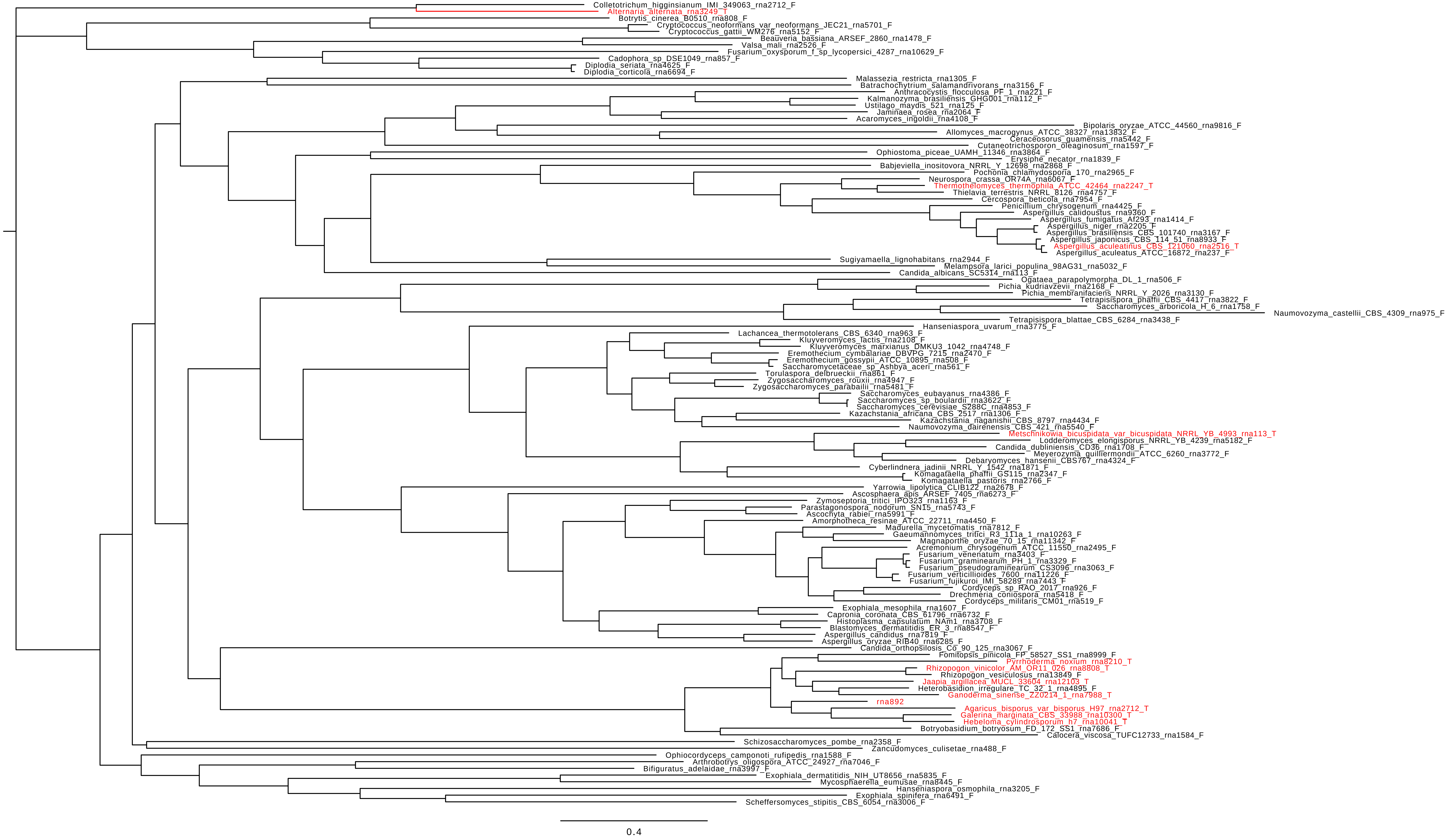

Colletotrichum_higginsianum_IMI_349063_rna2712_F
Alternaria_alternata_rna3249_T
Botrytis_cinerea_B0510_rna808_F
Cryptococcus_neoformans_var_neoformans_JEC21_rna5701_F
Cryptococcus_gattii_WM276_rna5152_F
Beauveria_bassiana_ARSEF_2860_rna1478_F
Valsa_mali_rna2526_F
Fusarium_oxysporum_f_sp_lycopersici_4287_rna10629_F
Cadophora_sp_DSE1049_rna857_F
Diplodia_seriata_rna4625_F
Diplodia_corticola_rna6694_F
Malassezia_restricta_rna1305_F
Batrachochytrium_salamandrivorans_rna3156_F
Anthracocystis_flocculosa_PF_1_rna221_F
Kalmanozyma_brasiliensis_GHG001_rna112_F
Ustilago_maydis_521_rna125_F
Jaminaea_rosea_rna2064_F
Acaromyces_ingoldii_rna4108_F
Bipolaris_oryzae_ATCC_44560_rna9816_F
Allomyces_macrogynus_ATCC_38327_rna13832_F
Ceraceosorus_guamensis_rna5442_F
Cutaneotrichosporon_oleaginosum_rna1597_F
Ophiostoma_piceae_UAMH_11346_rna3864_F
Erysiphe_necator_rna1839_F
Babjeviella_inositovora_NRRL_Y_12698_rna2868_F
Pochonia_chlamydosporia_170_rna2965_F
Neurospora_crassa_OR74A_rna6067_F
Thermothelomyces_thermophila_ATCC_42464_rna2247_T
Thielavia_terrestris_NRRL_8126_rna4757_F
Cercospora_beticola_rna7954_F
Penicillium_chrysogenum_rna4425_F
Aspergillus_calidoustus_rna9360_F
Aspergillus_fumigatus_Af293_rna1414_F
Aspergillus_niger_rna2205_F
Aspergillus_brasiliensis_CBS_101740_rna3167_F
Aspergillus_japonicus_CBS_114_51_rna8933_F
Aspergillus_aculeatinus_CBS_121060_rna2516_T
Aspergillus_aculeatus_ATCC_16872_rna237_F
Sugiyamaella_lignohabitans_rna2944_F
Melampsora_larici_populina_98AG31_rna5032_F
Candida_albicans_SC5314_rna113_F
Ogataea_parapolymorpha_DL_1_rna506_F
Pichia_kudriavzevii_rna2168_F
Pichia_membranifaciens_NRRL_Y_2026_rna3130_F
Tetrapisispora_phaffii_CBS_4417_rna3822_F
Saccharomyces_arboricola_H_6_rna1758_F
Naumovozyma_castellii_CBS_4309_rna975_F
Tetrapisispora_blattae_CBS_6284_rna3438_F
Hanseniaspora_uvarum_rna3775_F
Lachancea_thermotolerans_CBS_6340_rna963_F
Kluyveromyces_lactis_rna2108_F
Kluyveromyces_marxianus_DMKU3_1042_rna4748_F
Eremothecium_cymbalariae_DBVPG_7215_rna2470_F
Eremothecium_gossypii_ATCC_10895_rna508_F
Saccharomycetaceae_sp_Ashbya_aceri_rna561_F
Torulaspora_delbrueckii_rna861_F
Zygosaccharomyces_rouxii_rna4947_F
Zygosaccharomyces_parabailii_rna5481_F
Saccharomyces_eubayanus_rna4386_F
Saccharomyces_sp_boulardii_rna3622_F
Saccharomyces_cerevisiae_S288C_rna4853_F
Kazachstania_africana_CBS_2517_rna1306_F
Kazachstania_naganishii_CBS_8797_rna4434_F
Naumovozyma_dairenensis_CBS_421_rna5540_F
Metschnikowia_bicuspidata_var_bicuspidata_NRRL_YB_4993_rna113_T
Lodderomyces_elongisporus_NRRL_YB_4239_rna5182_F
Candida_dubliniensis_CD36_rna1708_F
Meyerozyma_guilliermondii_ATCC_6260_rna3772_F
Debaryomyces_hansenii_CBS767_rna4324_F
Cyberlindnera_jadinii_NRRL_Y_1542_rna1871_F
Komagataella_phaffii_GS115_rna2347_F
Komagataella_pastoris_rna2766_F
Yarrowia_lipolytica_CLIB122_rna2678_F
Ascosphaera_apis_ARSEF_7405_rna6273_F
Zymoseptoria_tritici_IPO323_rna1163_F
Parastagonospora_nodorum_SN15_rna5743_F
Ascochyta_rabiei_rna5991_F
Amorphotheca_resinae_ATCC_22711_rna4450_F
Madurella_mycetomatis_rna7812_F
Gaeumannomyces_tritici_R3_111a_1_rna10263_F
Magnaporthe_oryzae_70_15_rna11342_F
Acremonium_chrysogenum_ATCC_11550_rna2495_F
Fusarium_venenatum_rna3403_F
Fusarium_graminearum_PH_1_rna3329_F
Fusarium_pseudograminearum_CS3096_rna3063_F
Fusarium_verticillioides_7600_rna11226_F
Fusarium_fujikuroi_IMI_58289_rna7443_F
Cordyceps_sp_RAO_2017_rna926_F
Drechmeria_coniospora_rna5418_F
Cordyceps_militaris_CM01_rna519_F
Exophiala_mesophila_rna1607_F
Capronia_coronata_CBS_61796_rna6732_F
Histoplasma_capsulatum_NAm1_rna3708_F
Blastomyces_dermatitidis_ER_3_rna8547_F
Aspergillus_candidus_rna7819_F
Aspergillus_oryzae_RIB40_rna6285_F
Candida_orthopsilosis_Co_90_125_rna3067_F
Fomitopsis_pinicola_FP_58527_SS1_rna8999_F
Pyrrhoderma_noxium_rna8210_T
Rhizopogon_vinicolor_AM_OR11_026_rna8808_T
Rhizopogon_vesiculosus_rna13849_F
Jaapia_argillacea_MUCL_33604_rna12103_T
Heterobasidion_irregulare_TC_32_1_rna4895_F
Ganoderma_sinense_ZZ0214_1_rna7988_T
rna892
Agaricus_bisporus_var_bisporus_H97_rna2712_T
Galerina_marginata_CBS_33988_rna10300_T
Hebeloma_cylindrosporum_h7_rna10041_T
Botryobasidium_botryosum_FD_172_SS1_rna7686_F
Calocera_viscosa_TUFC12733_rna1584_F
Schizosaccharomyces_pombe_rna2358_F
Zancudomyces_culisetae_rna488_F
Ophiocordyceps_camponoti_rufipedis_rna1588_F
Arthrobotrys_oligospora_ATCC_24927_rna7046_F
Bifiguratus_adelaidae_rna3997_F
Exophiala_dermatitidis_NIH_UT8656_rna5835_F
Mycosphaerella_eumusae_rna8445_F
Hanseniaspora_osmophila_rna3205_F
Exophiala_spinifera_rna6491_F
Scheffersomyces_stipitis_CBS_6054_rna3006_F
0.4
