# Supplementary material for: Animal, Fungi, and Plant Genome Sequences Harbor Different Non-Canonical Splice Sites
Source: Cells. 2020 Feb 18;9(2):458. doi: 10.3390/cells9020458 (PMC7072748; doi:10.3390/cells9020458)
Supplement: Supplementary file 1 [file cells-09-00458-s001.zip › supplements/File S19.zip!/files_for_S19/1133.pdf]

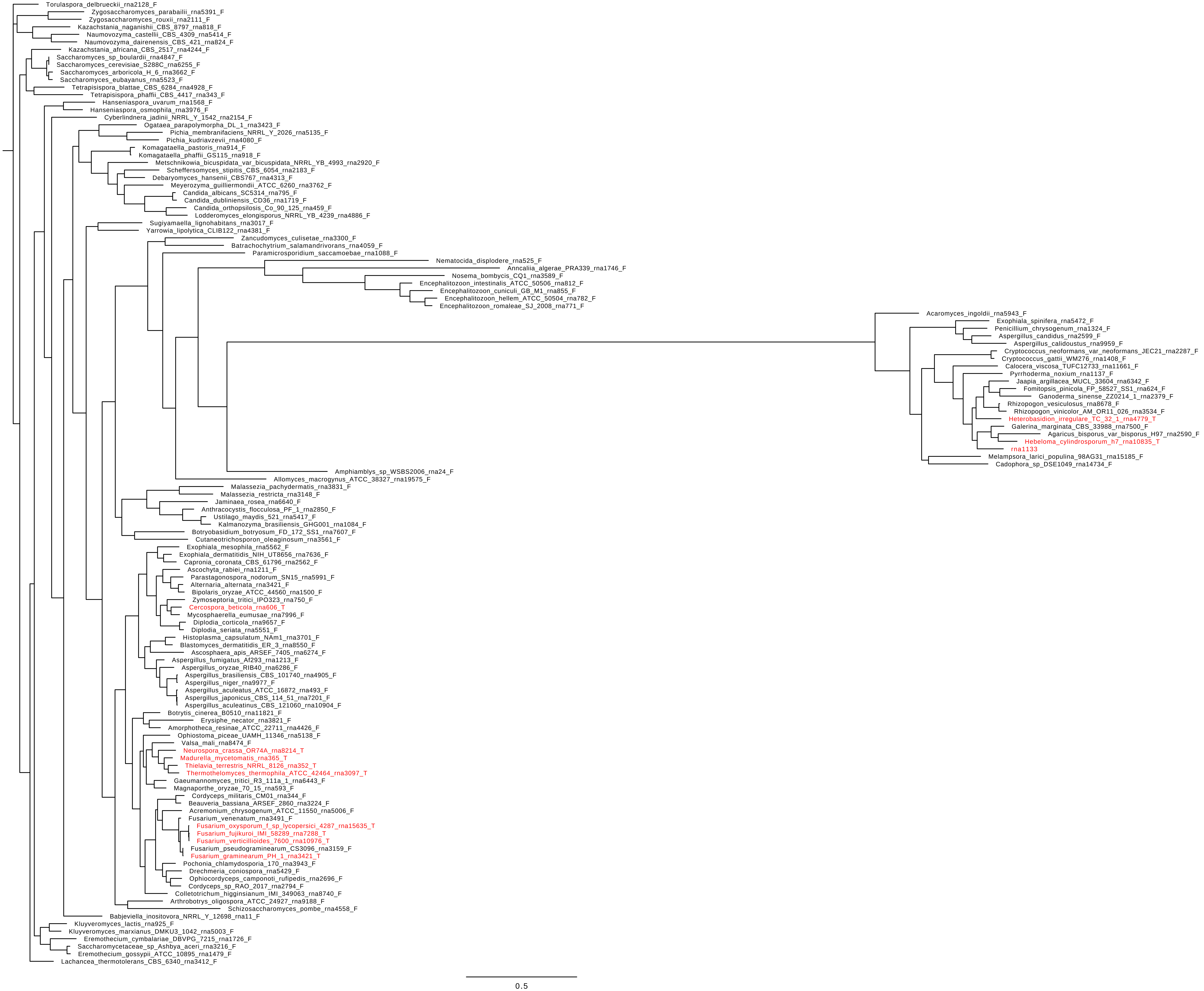

Torulaspora_delbrueckii_rna2128_F
Zygosaccharomyces_parabailii_rna5391_F
Zygosaccharomyces_rouxii_rna2111_F
Kazachstania_naganishii_CBS_8797_rna818_F
Naumovozyma_castellii_CBS_4309_rna5414_F
Naumovozyma_dairenensis_CBS_421_rna824_F
Kazachstania_africana_CBS_2517_rna4244_F
Saccharomyces_sp_boulardii_rna4847_F
Saccharomyces_cerevisiae_S288C_rna6255_F
Saccharomyces_arboricola_H_6_rna3662_F
Saccharomyces_eubayanus_rna5523_F
Tetrapisispora_blattae_CBS_6284_rna4928_F
Tetrapisispora_phaffii_CBS_4417_rna343_F
Hanseniaspora_uvarum_rna1568_F
Hanseniaspora_osmophila_rna3976_F
Cyberlindnera_jadinii_NRRL_Y_1542_rna2154_F
Ogataea_parapolymorpha_DL_1_rna3423_F
Pichia_membranifaciens_NRRL_Y_2026_rna5135_F
Pichia_kudriavzevii_rna4080_F
Komagataella_pastoris_rna914_F
Komagataella_phaffii_GS115_rna918_F
Metschnikowia_bicuspidata_var_bicuspidata_NRRL_YB_4993_rna2920_F
Scheffersomyces_stipitis_CBS_6054_rna2183_F
Debaryomyces_hansenii_CBS767_rna4313_F
Meyerozyma_guilliermondii_ATCC_6260_rna3762_F
Candida_albicans_SC5314_rna795_F
Candida_dubliniensis_CD36_rna1719_F
Candida_orthopsilosis_Co_90_125_rna459_F
Lodderomyces_elongisporus_NRRL_YB_4239_rna4886_F
Sugiyamaella_lignohabitans_rna3017_F
Yarrowia_lipolytica_CLIB122_rna4381_F
Zancudomyces_culisetae_rna3300_F
Batrachochytrium_salamandrivorans_rna4059_F
Paramicrosporidium_saccamoebae_rna1088_F
Nematocida_displodere_rna525_F
Anncaliia_algerae_PRA339_rna1746_F
Nosema_bombycis_CQ1_rna3589_F
Encephalitozoon_intestinalis_ATCC_50506_rna812_F
Encephalitozoon_cuniculi_GB_M1_rna855_F
Encephalitozoon_hellem_ATCC_50504_rna782_F
Encephalitozoon_romaleae_SJ_2008_rna771_F
Acaromyces_ingoldii_rna5943_F
Exophiala_spinifera_rna5472_F
Penicillium_chrysogenum_rna1324_F
Aspergillus_candidus_rna2599_F
Aspergillus_calidoustus_rna9959_F
Cryptococcus_neoformans_var_neoformans_JEC21_rna2287_F
Cryptococcus_gattii_WM276_rna1408_F
Calocera_viscosa_TUFC12733_rna11661_F
Pyrrhoderma_noxium_rna1137_F
Jaapia_argillacea_MUCL_33604_rna6342_F
Fomitopsis_pinicola_FP_58527_SS1_rna624_F
Ganoderma_sinense_ZZ0214_1_rna2379_F
Rhizopogon_vesiculosus_rna8678_F
Rhizopogon_vinicolor_AM_OR11_026_rna3534_F
Heterobasidion_irregulare_TC_32_1_rna4779_T
Galerina_marginata_CBS_33988_rna7500_F
Agaricus_bisporus_var_bisporus_H97_rna2590_F
Hebeloma_cylindrosporum_h7_rna10835_T
rna1133
Melampsora_larici_populina_98AG31_rna15185_F
Cadophora_sp_DSE1049_rna14734_F
Amphiamblys_sp_WSBS2006_rna24_F
Allomyces_macrogynus_ATCC_38327_rna19575_F
Malassezia_pachydermatis_rna3831_F
Malassezia_restricta_rna3148_F
Jaminaea_rosea_rna6640_F
Anthracocystis_flocculosa_PF_1_rna2850_F
Ustilago_maydis_521_rna5417_F
Kalmanozyma_brasiliensis_GHG001_rna1084_F
Botryobasidium_botryosum_FD_172_SS1_rna7607_F
Cutaneotrichosporon_oleaginosum_rna3561_F
Exophiala_mesophila_rna5562_F
Exophiala_dermatitidis_NIH_UT8656_rna7636_F
Capronia_coronata_CBS_61796_rna2562_F
Ascochyta_rabiei_rna1211_F
Parastagonospora_nodorum_SN15_rna5991_F
Alternaria_alternata_rna3421_F
Bipolaris_oryzae_ATCC_44560_rna1500_F
Zymoseptoria_tritici_IPO323_rna750_F
Cercospora_beticola_rna606_T
Mycosphaerella_eumusae_rna7996_F
Diplodia_corticola_rna9657_F
Diplodia_seriata_rna5551_F
Histoplasma_capsulatum_NAm1_rna3701_F
Blastomyces_dermatitidis_ER_3_rna8550_F
Ascosphaera_apis_ARSEF_7405_rna6274_F
Aspergillus_fumigatus_Af293_rna1213_F
Aspergillus_oryzae_RIB40_rna6286_F
Aspergillus_brasiliensis_CBS_101740_rna4905_F
Aspergillus_niger_rna9977_F
Aspergillus_aculeatus_ATCC_16872_rna493_F
Aspergillus_japonicus_CBS_114_51_rna7201_F
Aspergillus_aculeatinus_CBS_121060_rna10904_F
Botrytis_cinerea_B0510_rna11821_F
Erysiphe_necator_rna3821_F
Amorphotheca_resinae_ATCC_22711_rna4426_F
Ophiostoma_piceae_UAMH_11346_rna5138_F
Valsa_mali_rna8474_F
Neurospora_crassa_OR74A_rna8214_T
Madurella_mycetomatis_rna365_T
Thielavia_terrestris_NRRL_8126_rna352_T
Thermothelomyces_thermophila_ATCC_42464_rna3097_T
Gaeumannomyces_tritici_R3_111a_1_rna6443_F
Magnaporthe_oryzae_70_15_rna593_F
Cordyceps_militaris_CM01_rna344_F
Beauveria_bassiana_ARSEF_2860_rna3224_F
Acremonium_chrysogenum_ATCC_11550_rna5006_F
Fusarium_venenatum_rna3491_F
Fusarium_oxysporum_f_sp_lycopersici_4287_rna15635_T
Fusarium_fujikuroi_IMI_58289_rna7288_T
Fusarium_verticillioides_7600_rna10976_T
Fusarium_pseudograminearum_CS3096_rna3159_F
Fusarium_graminearum_PH_1_rna3421_T
Pochonia_chlamydosporia_170_rna3943_F
Drechmeria_coniospora_rna5429_F
Ophiocordyceps_camponoti_rufipedis_rna2696_F
Cordyceps_sp_RAO_2017_rna2794_F
Colletotrichum_higginsianum_IMI_349063_rna8740_F
Arthrobotrys_oligospora_ATCC_24927_rna9188_F
Schizosaccharomyces_pombe_rna4558_F
Babjeviella_inositovora_NRRL_Y_12698_rna11_F
Kluyveromyces_lactis_rna925_F
Kluyveromyces_marxianus_DMKU3_1042_rna5003_F
Eremothecium_cymbalariae_DBVPG_7215_rna1726_F
Saccharomycetaceae_sp_Ashbya_aceri_rna3216_F
Eremothecium_gossypii_ATCC_10895_rna1479_F
Lachancea_thermotolerans_CBS_6340_rna3412_F
0.5
