# Supplementary material for: Animal, Fungi, and Plant Genome Sequences Harbor Different Non-Canonical Splice Sites
Source: Cells. 2020 Feb 18;9(2):458. doi: 10.3390/cells9020458 (PMC7072748; doi:10.3390/cells9020458)
Supplement: Supplementary file 1 [file cells-09-00458-s001.zip › supplements/File S19.zip!/files_for_S19/2438.pdf]

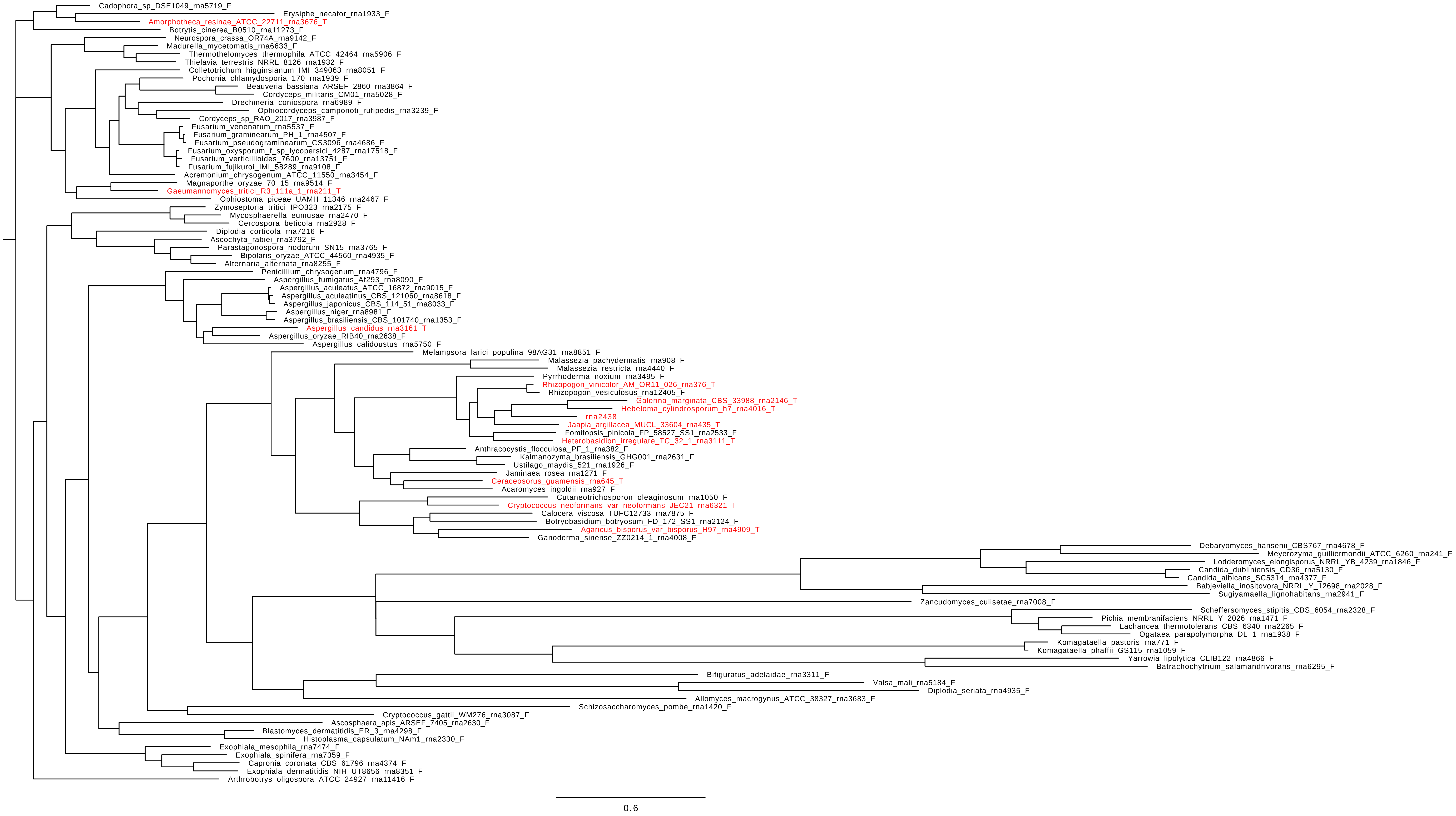
Cadophora_sp_DSE1049_rna5719_F
Erysiphe_necator_rna1933_F
Amorphotheca_resinae_ATCC_22711_rna3676_T
Botrytis_cinerea_B0510_rna11273_F
Neurospora_crassa_OR74A_rna9142_F
Madurella_mycetomatis_rna6633_F
Thermothelomyces_thermophila_ATCC_42464_rna5906_F
Thielavia_terrestris_NRRL_8126_rna1932_F
Colletotrichum_higginsianum_IMI_349063_rna8051_F
Pochonia_chlamydosporia_170_rna1939_F
Beauveria_bassiana_ARSEF_2860_rna3864_F
Cordyceps_militaris_CM01_rna5028_F
Drechmeria_coniospora_rna6989_F
Ophiocordyceps_camponoti_rufipedis_rna3239_F
Cordyceps_sp_RAO_2017_rna3987_F
Fusarium_venenatum_rna5537_F
Fusarium_graminearum_PH_1_rna4507_F
Fusarium_pseudograminearum_CS3096_rna4686_F
Fusarium_oxysporum_f_sp_lycopersici_4287_rna17518_F
Fusarium_verticillioides_7600_rna13751_F
Fusarium_fujikuroi_IMI_58289_rna9108_F
Acremonium_chrysogenum_ATCC_11550_rna3454_F
Magnaporthe_oryzae_70_15_rna9514_F
Gaeumannomyces_tritici_R3_111a_1_rna211_T
Ophiostoma_piceae_UAMH_11346_rna2467_F
Zymoseptoria_tritici_IPO323_rna2175_F
Mycosphaerella_eumusae_rna2470_F
Cercospora_beticola_rna2928_F
Diplodia_corticola_rna7216_F
Ascochyta_rabiei_rna3792_F
Parastagonospora_nodorum_SN15_rna3765_F
Bipolaris_oryzae_ATCC_44560_rna4935_F
Alternaria_alternata_rna8255_F
Penicillium_chrysogenum_rna4796_F
Aspergillus_fumigatus_Af293_rna8090_F
Aspergillus_aculeatus_ATCC_16872_rna9015_F
Aspergillus_aculeatinus_CBS_121060_rna8618_F
Aspergillus_japonicus_CBS_114_51_rna8033_F
Aspergillus_niger_rna8981_F
Aspergillus_brasiliensis_CBS_101740_rna1353_F
Aspergillus_candidus_rna3161_T
Aspergillus_oryzae_RIB40_rna2638_F
Aspergillus_calidoustus_rna5750_F
Melampsora_larici_populina_98AG31_rna8851_F
Malassezia_pachydermatis_rna908_F
Malassezia_restricta_rna4440_F
Pyrrhoderma_noxium_rna3495_F
Rhizopogon_vinicolor_AM_OR11_026_rna376_T
Rhizopogon_vesiculosus_rna12405_F
Galerina_marginata_CBS_33988_rna2146_T
Hebeloma_cylindrosporum_h7_rna4016_T
rna2438
Jaapia_argillacea_MUCL_33604_rna435_T
Fomitopsis_pinicola_FP_58527_SS1_rna2533_F
Heterobasidion_irregulare_TC_32_1_rna3111_T
Anthracocystis_flocculosa_PF_1_rna382_F
Kalmanozyma_brasiliensis_GHG001_rna2631_F
Ustilago_maydis_521_rna1926_F
Jaminaea_rosea_rna1271_F
Ceraceosorus_guamensis_rna645_T
Acaromyces_ingoldii_rna927_F
Cutaneotrichosporon_oleaginosum_rna1050_F
Cryptococcus_neoformans_var_neoformans_JEC21_rna6321_T
Calocera_viscosa_TUFC12733_rna7875_F
Botryobasidium_botryosum_FD_172_SS1_rna2124_F
Agaricus_bisporus_var_bisporus_H97_rna4909_T
Ganoderma_sinense_ZZ0214_1_rna4008_F
Debaryomyces_hansenii_CBS767_rna4678_F
Meyerozyma_guilliermondii_ATCC_6260_rna241_F
Lodderomyces_elongisporus_NRRL_YB_4239_rna1846_F
Candida_dubliniensis_CD36_rna5130_F
Candida_albicans_SC5314_rna4377_F
Babjeviella_inositovora_NRRL_Y_12698_rna2028_F
Sugiyamaella_lignohabitans_rna2941_F
Zancudomyces_culisetae_rna7008_F
Scheffersomyces_stipitis_CBS_6054_rna2328_F
Pichia_membranifaciens_NRRL_Y_2026_rna1471_F
Lachancea_thermotolerans_CBS_6340_rna2265_F
Ogataea_parapolymorpha_DL_1_rna1938_F
Komagataella_pastoris_rna771_F
Komagataella_phaffii_GS115_rna1059_F
Yarrowia_lipolytica_CLIB122_rna4866_F
Batrachochytrium_salamandrivorans_rna6295_F
Bifiguratus_adelaidae_rna3311_F
Valsa_mali_rna5184_F
Diplodia_seriata_rna4935_F
Allomyces_macrogynus_ATCC_38327_rna3683_F
Schizosaccharomyces_pombe_rna1420_F
Cryptococcus_gattii_WM276_rna3087_F
Ascosphaera_apis_ARSEF_7405_rna2630_F
Blastomyces_dermatitidis_ER_3_rna4298_F
Histoplasma_capsulatum_NAm1_rna2330_F
Exophiala_mesophila_rna7474_F
Exophiala_spinifera_rna7359_F
Capronia_coronata_CBS_61796_rna4374_F
Exophiala_dermatitidis_NIH_UT8656_rna8351_F
Arthrobotrys_oligospora_ATCC_24927_rna11416_F
0.6
