# Supplementary material for: Animal, Fungi, and Plant Genome Sequences Harbor Different Non-Canonical Splice Sites
Source: Cells. 2020 Feb 18;9(2):458. doi: 10.3390/cells9020458 (PMC7072748; doi:10.3390/cells9020458)
Supplement: Supplementary file 1 [file cells-09-00458-s001.zip › supplements/File S19.zip!/files_for_S19/2669.pdf]

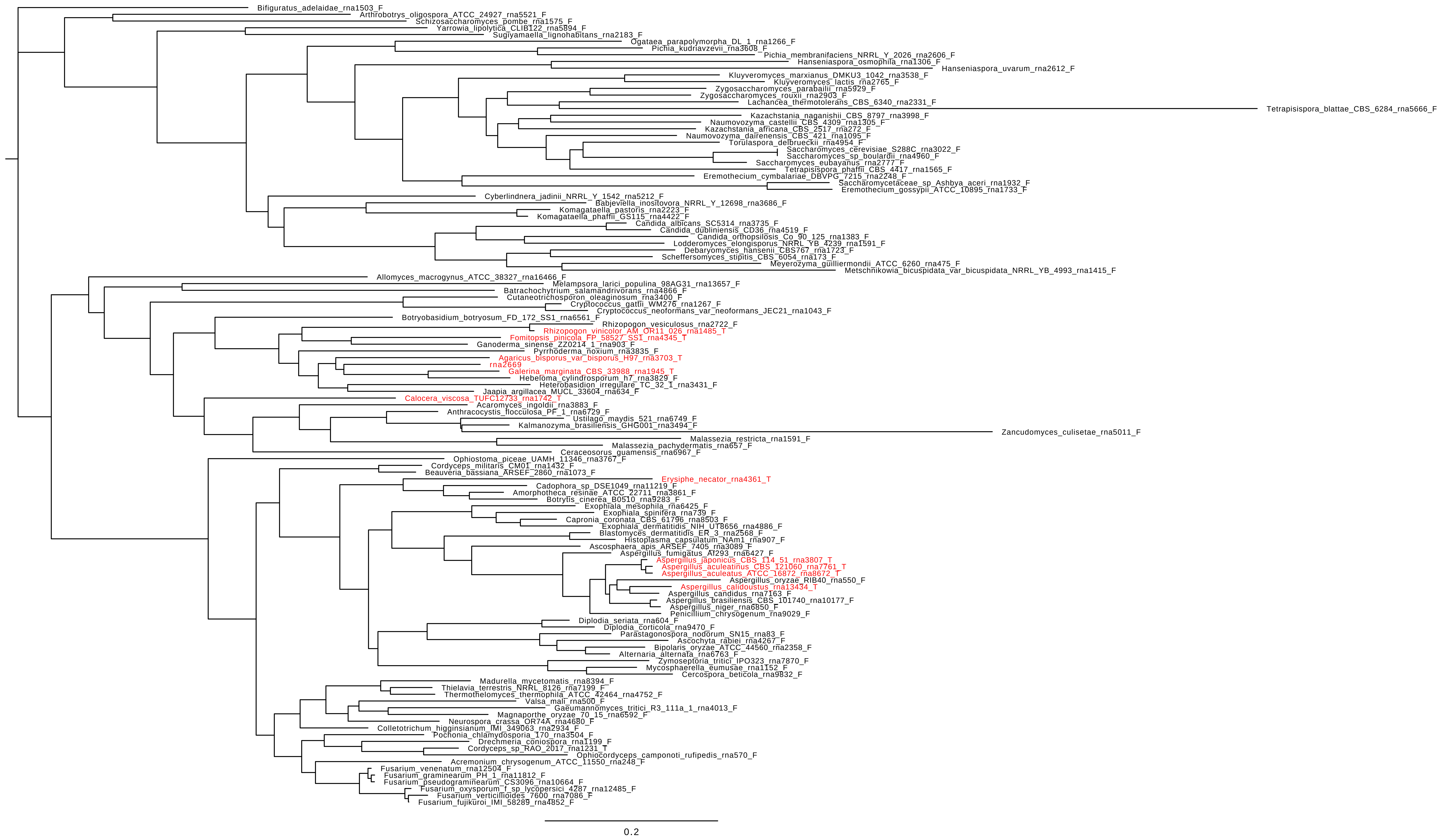

Bifiguratus_adelaidae_rna1503_F
Arthrobotrys_oligospora_ATCC_24927_rna5521_F
Schizosaccharomyces_pombe_rna1575_F
Yarrowia_lipolytica_CLIB122_rna5894_F
Sugiyamaella_lignohabitans_rna2183_F
Ogataea_parapolymorpha_DL_1_rna1266_F
Pichia_kudriavzevii_rna3608_F
Pichia_membranifaciens_NRRL_Y_2026_rna2606_F
Hanseniaspora_osmophila_rna1306_F
Hanseniaspora_uvarum_rna2612_F
Kluyveromyces_marxianus_DMKU3_1042_rna3538_F
Kluyveromyces_lactis_rna2765_F
Zygosaccharomyces_parabailii_rna5929_F
Zygosaccharomyces_rouxii_rna2903_F
Lachancea_thermotolerans_CBS_6340_rna2331_F
Tetrapisispora_blattae_CBS_6284_rna5666_F
Kazachstania_naganishii_CBS_8797_rna3998_F
Naumovozyma_castellii_CBS_4309_rna1305_F
Kazachstania_africana_CBS_2517_rna272_F
Naumovozyma_dairenensis_CBS_421_rna1095_F
Torulaspora_delbrueckii_rna4954_F
Saccharomyces_cerevisiae_S288C_rna3022_F
Saccharomyces_sp_boulardii_rna4960_F
Saccharomyces_eubayanus_rna2777_F
Tetrapisispora_phaffii_CBS_4417_rna1565_F
Eremothecium_cymbalariae_DBVPG_7215_rna2248_F
Saccharomycetaceae_sp_Ashbya_aceri_rna1932_F
Eremothecium_gossypii_ATCC_10895_rna1733_F
Cyberlindnera_jadinii_NRRL_Y_1542_rna5212_F
Babjeviella_inositovora_NRRL_Y_12698_rna3686_F
Komagataella_pastoris_rna2223_F
Komagataella_phaffii_GS115_rna4422_F
Candida_albicans_SC5314_rna3735_F
Candida_dubliniensis_CD36_rna4519_F
Candida_orthopsilosis_Co_90_125_rna1383_F
Lodderomyces_elongisporus_NRRL_YB_4239_rna1591_F
Debaryomyces_hansenii_CBS767_rna1723_F
Scheffersomyces_stipitis_CBS_6054_rna173_F
Meyerozyma_guilliermondii_ATCC_6260_rna475_F
Metschnikowia_bicuspidata_var_bicuspidata_NRRL_YB_4993_rna1415_F
Allomyces_macrogynus_ATCC_38327_rna16466_F
Melampsora_larici_populina_98AG31_rna13657_F
Batrachochytrium_salamandrivorans_rna4866_F
Cutaneotrichosporon_oleaginosum_rna3400_F
Cryptococcus_gattii_WM276_rna1267_F
Cryptococcus_neoformans_var_neoformans_JEC21_rna1043_F
Botryobasidium_botryosum_FD_172_SS1_rna6561_F
Rhizopogon_vesiculosus_rna2722_F
Rhizopogon_vinicolor_AM_OR11_026_rna1485_T
Fomitopsis_pinicola_FP_58527_SS1_rna4345_T
Ganoderma_sinense_ZZ0214_1_rna903_F
Pyrrhoderma_noxium_rna3835_F
Agaricus_bisporus_var_bisporus_H97_rna3703_T
rna2669
Galerina_marginata_CBS_33988_rna1945_T
Hebeloma_cylindrosporum_h7_rna3829_F
Heterobasidion_irregulare_TC_32_1_rna3431_F
Jaapia_argillacea_MUCL_33604_rna634_F
Calocera_viscosa_TUFC12733_rna1742_T
Acaromyces_ingoldii_rna3883_F
Anthracocystis_flocculosa_PF_1_rna6729_F
Ustilago_maydis_521_rna6749_F
Kalmanozyma_brasiliensis_GHG001_rna3494_F
Zancudomyces_culisetae_rna5011_F
Malassezia_restricta_rna1591_F
Malassezia_pachydermatis_rna657_F
Ceraceosorus_guamensis_rna6967_F
Ophiostoma_piceae_UAMH_11346_rna3767_F
Cordyceps_militaris_CM01_rna1432_F
Beauveria_bassiana_ARSEF_2860_rna1073_F
Erysiphe_necator_rna4361_T
Cadophora_sp_DSE1049_rna11219_F
Amorphotheca_resinae_ATCC_22711_rna3861_F
Botrytis_cinerea_B0510_rna9283_F
Exophiala_mesophila_rna6425_F
Exophiala_spinifera_rna739_F
Capronia_coronata_CBS_61796_rna8503_F
Exophiala_dermatitidis_NIH_UT8656_rna4886_F
Blastomyces_dermatitidis_ER_3_rna2568_F
Histoplasma_capsulatum_NAm1_rna907_F
Ascosphaera_apis_ARSEF_7405_rna3089_F
Aspergillus_fumigatus_Af293_rna6427_F
Aspergillus_japonicus_CBS_114_51_rna3807_T
Aspergillus_aculeatinus_CBS_121060_rna7761_T
Aspergillus_aculeatus_ATCC_16872_rna8672_T
Aspergillus_oryzae_RIB40_rna550_F
Aspergillus_calidoustus_rna13434_T
Aspergillus_candidus_rna7163_F
Aspergillus_brasiliensis_CBS_101740_rna10177_F
Aspergillus_niger_rna6850_F
Penicillium_chrysogenum_rna9029_F
Diplodia_seriata_rna604_F
Diplodia_corticola_rna9470_F
Parastagonospora_nodorum_SN15_rna83_F
Ascochyta_rabiei_rna4267_F
Bipolaris_oryzae_ATCC_44560_rna2358_F
Alternaria_alternata_rna6763_F
Zymoseptoria_tritici_IPO323_rna7870_F
Mycosphaerella_eumusae_rna1152_F
Cercospora_beticola_rna9832_F
Madurella_mycetomatis_rna8394_F
Thielavia_terrestris_NRRL_8126_rna7199_F
Thermothelomyces_thermophila_ATCC_42464_rna4752_F
Valsa_mali_rna500_F
Gaeumannomyces_tritici_R3_111a_1_rna4013_F
Magnaporthe_oryzae_70_15_rna6592_F
Neurospora_crassa_OR74A_rna4680_F
Colletotrichum_higginsianum_IMI_349063_rna2934_F
Pochonia_chlamydosporia_170_rna3504_F
Drechmeria_coniospora_rna1199_F
Cordyceps_sp_RAO_2017_rna1231_T
Ophiocordyceps_camponoti_rufipedis_rna570_F
Acremonium_chrysogenum_ATCC_11550_rna248_F
Fusarium_venenatum_rna12504_F
Fusarium_graminearum_PH_1_rna11812_F
Fusarium_pseudograminearum_CS3096_rna10664_F
Fusarium_oxysporum_f_sp_lycopersici_4287_rna12485_F
Fusarium_verticillioides_7600_rna7086_F
Fusarium_fujikuroi_IMI_58289_rna4852_F
0.2
