# Supplementary material for: Animal, Fungi, and Plant Genome Sequences Harbor Different Non-Canonical Splice Sites
Source: Cells. 2020 Feb 18;9(2):458. doi: 10.3390/cells9020458 (PMC7072748; doi:10.3390/cells9020458)
Supplement: Supplementary file 1 [file cells-09-00458-s001.zip › supplements/File S19.zip!/files_for_S19/3444.pdf]

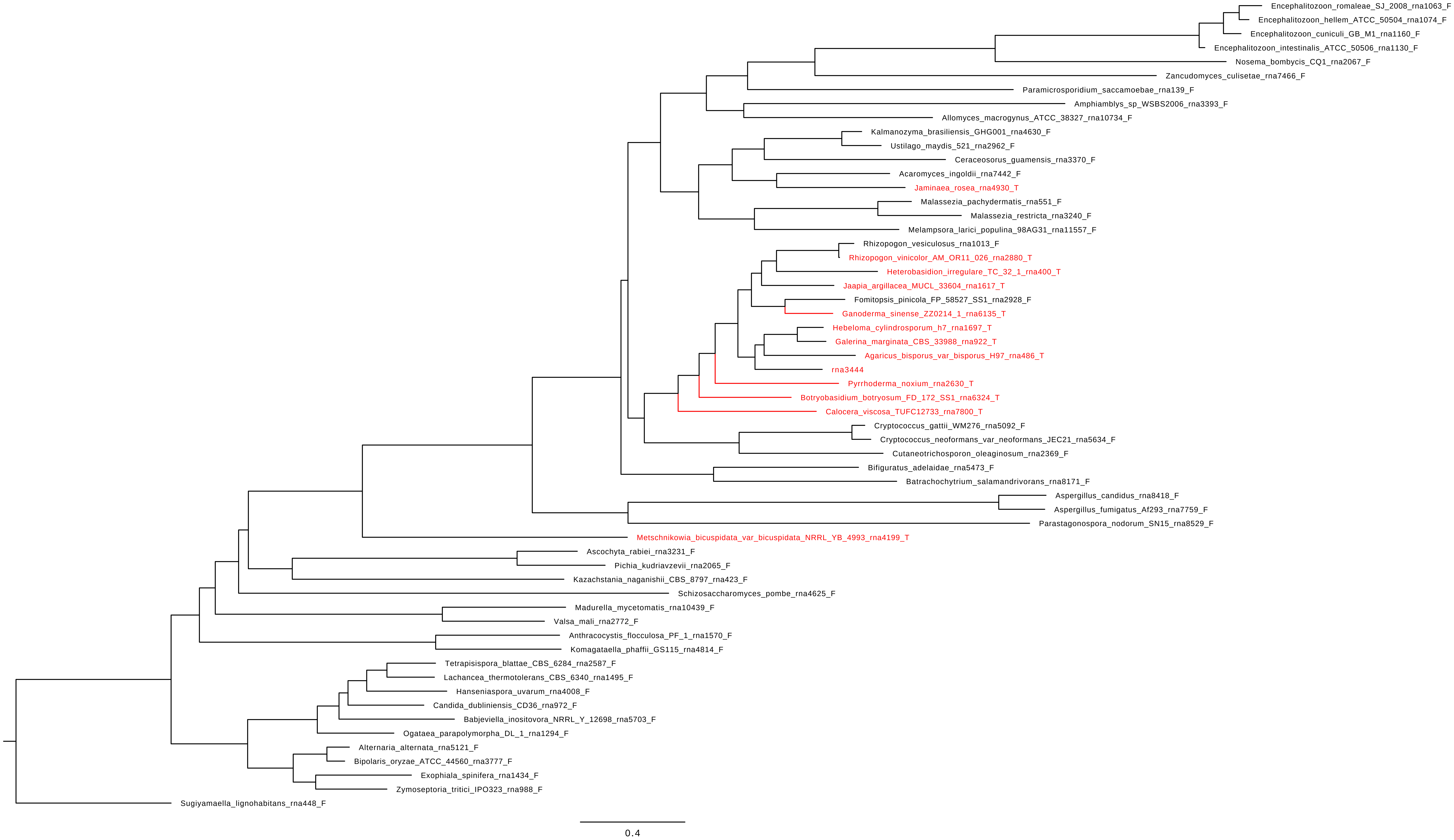
Encephalitozoon_romaleae_SJ_2008_rna1063_F
Encephalitozoon_hellem_ATCC_50504_rna1074_F
Encephalitozoon_cuniculi_GB_M1_rna1160_F
Encephalitozoon_intestinalis_ATCC_50506_rna1130_F
Nosema_bombycis_CQ1_rna2067_F
Zancudomyces_culisetae_rna7466_F
Paramicrosporidium_saccamoebae_rna139_F
Amphiamblys_sp_WSBS2006_rna3393_F
Allomyces_macrogynus_ATCC_38327_rna10734_F
Kalmanozyma_brasiliensis_GHG001_rna4630_F
Ustilago_maydis_521_rna2962_F
Ceraceosorus_guamensis_rna3370_F
Acaromyces_ingoldii_rna7442_F
Jaminaea_rosea_rna4930_T
Malassezia_pachydermatis_rna551_F
Malassezia_restricta_rna3240_F
Melampsora_larici_populina_98AG31_rna11557_F
Rhizopogon_vesiculosus_rna1013_F
Rhizopogon_vinicolor_AM_OR11_026_rna2880_T
Heterobasidion_irregulare_TC_32_1_rna400_T
Jaapia_argillacea_MUCL_33604_rna1617_T
Fomitopsis_pinicola_FP_58527_SS1_rna2928_F
Ganoderma_sinense_ZZ0214_1_rna6135_T
Hebeloma_cylindrosporum_h7_rna1697_T
Galerina_marginata_CBS_33988_rna922_T
Agaricus_bisporus_var_bisporus_H97_rna486_T
rna3444
Pyrrhoderma_noxium_rna2630_T
Botryobasidium_botryosum_FD_172_SS1_rna6324_T
Calocera_viscosa_TUFC12733_rna7800_T
Cryptococcus_gattii_WM276_rna5092_F
Cryptococcus_neoformans_var_neoformans_JEC21_rna5634_F
Cutaneotrichosporon_oleaginosum_rna2369_F
Bifiguratus_adelaidae_rna5473_F
Batrachochytrium_salamandrivorans_rna8171_F
Aspergillus_candidus_rna8418_F
Aspergillus_fumigatus_Af293_rna7759_F
Parastagonospora_nodorum_SN15_rna8529_F
Metschnikowia_bicuspidata_var_bicuspidata_NRRL_YB_4993_rna4199_T
Ascochyta_rabiei_rna3231_F
Pichia_kudriavzevii_rna2065_F
Kazachstania_naganishii_CBS_8797_rna423_F
Schizosaccharomyces_pombe_rna4625_F
Madurella_mycetomatis_rna10439_F
Valsa_mali_rna2772_F
Anthracocystis_flocculosa_PF_1_rna1570_F
Komagataella_phaffii_GS115_rna4814_F
Tetrapisispora_blattae_CBS_6284_rna2587_F
Lachancea_thermotolerans_CBS_6340_rna1495_F
Hanseniaspora_uvarum_rna4008_F
Candida_dubliniensis_CD36_rna972_F
Babjeviella_inositovora_NRRL_Y_12698_rna5703_F
Ogataea_parapolymorpha_DL_1_rna1294_F
Alternaria_alternata_rna5121_F
Bipolaris_oryzae_ATCC_44560_rna3777_F
Exophiala_spinifera_rna1434_F
Zymoseptoria_tritici_IPO323_rna988_F
Sugiyamaella_lignohabitans_rna448_F
0.4
