# Supplementary material for: Animal, Fungi, and Plant Genome Sequences Harbor Different Non-Canonical Splice Sites
Source: Cells. 2020 Feb 18;9(2):458. doi: 10.3390/cells9020458 (PMC7072748; doi:10.3390/cells9020458)
Supplement: Supplementary file 1 [file cells-09-00458-s001.zip › supplements/File S19.zip!/files_for_S19/6928.pdf]

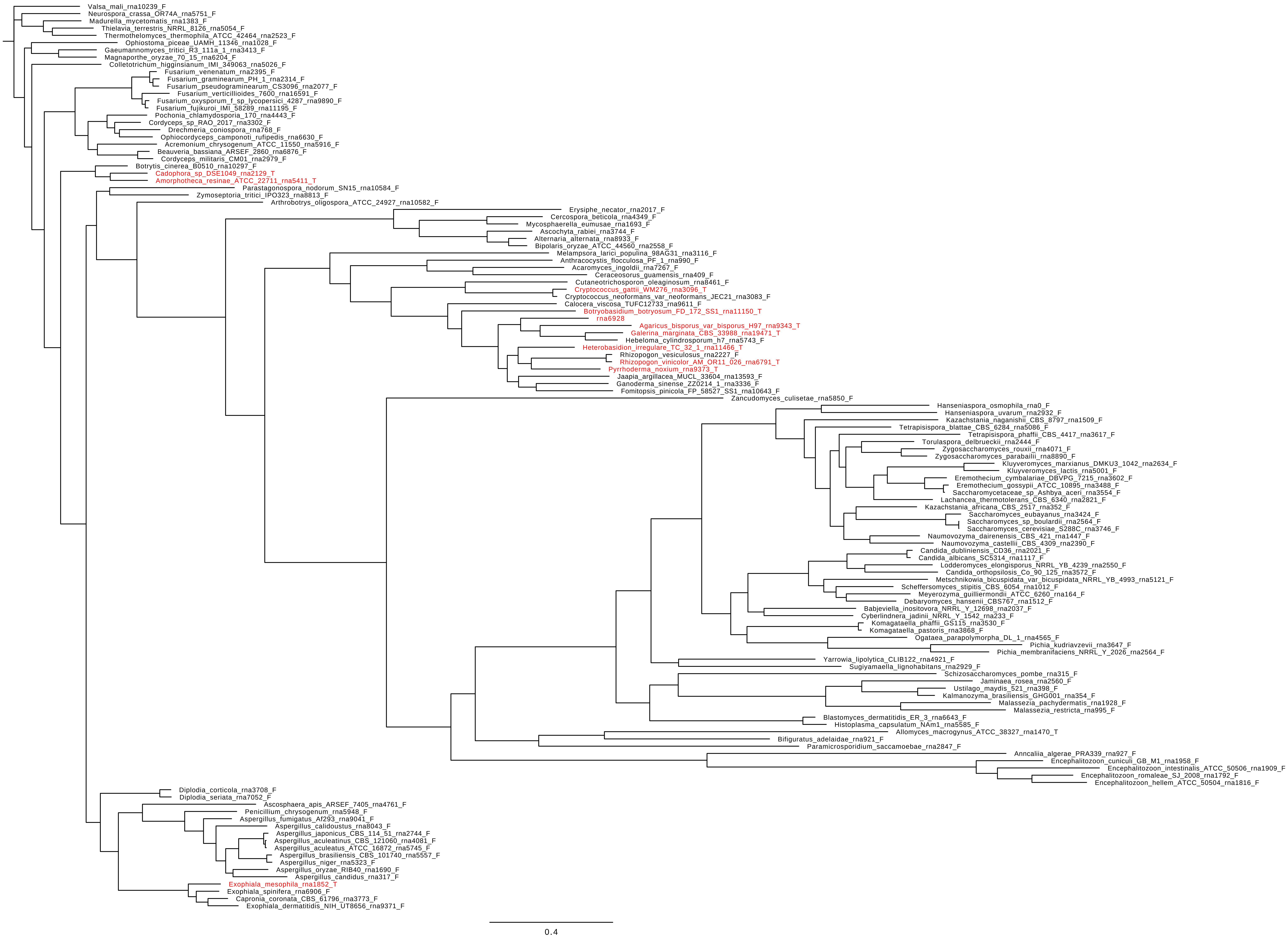

Valsa_mali_rna10239_F
Neurospora_crassa_OR74A_rna5751_F
Madurella_mycetomatis_rna1383_F
Thielavia_terrestris_NRRL_8126_rna5054_F
Thermothelomyces_thermophila_ATCC_42464_rna2523_F
Ophiostoma_piceae_UAMH_11346_rna1028_F
Gaeumannomyces_tritici_R3_111a_1_rna3413_F
Magnaporthe_oryzae_70_15_rna6204_F
Colletotrichum_higginsianum_IMI_349063_rna5026_F
Fusarium_venenatum_rna2395_F
Fusarium_graminearum_PH_1_rna2314_F
Fusarium_pseudograminearum_CS3096_rna2077_F
Fusarium_verticillioides_7600_rna16591_F
Fusarium_oxysporum_f_sp_lycopersici_4287_rna9890_F
Fusarium_fujikuroi_IMI_58289_rna11195_F
Pochonia_chlamydosporia_170_rna4443_F
Cordyceps_sp_RAO_2017_rna3302_F
Drechmeria_coniospora_rna768_F
Ophiocordyceps_camponoti_rufipedis_rna6630_F
Acremonium_chrysogenum_ATCC_11550_rna5916_F
Beauveria_bassiana_ARSEF_2860_rna6876_F
Cordyceps_militaris_CM01_rna2979_F
Botrytis_cinerea_B0510_rna10297_F
Cadophora_sp_DSE1049_rna2129_T
Amorphotheca_resinae_ATCC_22711_rna5411_T
Parastagonospora_nodorum_SN15_rna10584_F
Zymoseptoria_tritici_IPO323_rna8813_F
Arthrobotrys_oligospora_ATCC_24927_rna10582_F
Erysiphe_necator_rna2017_F
Cercospora_beticola_rna4349_F
Mycosphaerella_eumusae_rna1693_F
Ascochyta_rabiei_rna3744_F
Alternaria_alternata_rna8933_F
Bipolaris_oryzae_ATCC_44560_rna2558_F
Melampsora_larici_populina_98AG31_rna3116_F
Anthracocystis_flocculosa_PF_1_rna990_F
Acaromyces_ingoldii_rna7267_F
Ceraceosorus_guamensis_rna409_F
Cutaneotrichosporon_oleaginosum_rna8461_F
Cryptococcus_gattii_WM276_rna3096_T
Cryptococcus_neoformans_var_neoformans_JEC21_rna3083_F
Calocera_viscosa_TUFC12733_rna9611_F
Botryobasidium_botryosum_FD_172_SS1_rna11150_T
rna6928
Agaricus_bisporus_var_bisporus_H97_rna9343_T
Galerina_marginata_CBS_33988_rna19471_T
Hebeloma_cylindrosporum_h7_rna5743_F
Heterobasidion_irregulare_TC_32_1_rna11466_T
Rhizopogon_vesiculosus_rna2227_F
Rhizopogon_vinicolor_AM_OR11_026_rna6791_T
Pyrrhoderma_noxium_rna9373_T
Jaapia_argillacea_MUCL_33604_rna13593_F
Ganoderma_sinense_ZZ0214_1_rna3336_F
Fomitopsis_pinicola_FP_58527_SS1_rna10643_F
Zancudomyces_culisetae_rna5850_F
Hanseniaspora_osmophila_rna0_F
Hanseniaspora_uvarum_rna2932_F
Kazachstania_naganishii_CBS_8797_rna1509_F
Tetrapisispora_blattae_CBS_6284_rna5086_F
Tetrapisispora_phaffii_CBS_4417_rna3617_F
Torulaspora_delbrueckii_rna2444_F
Zygosaccharomyces_rouxii_rna4071_F
Zygosaccharomyces_parabailii_rna8890_F
Kluyveromyces_marxianus_DMKU3_1042_rna2634_F
Kluyveromyces_lactis_rna5001_F
Eremothecium_cymbalariae_DBVPG_7215_rna3602_F
Eremothecium_gossypii_ATCC_10895_rna3488_F
Saccharomycetaceae_sp_Ashbya_aceri_rna3554_F
Lachancea_thermotolerans_CBS_6340_rna2821_F
Kazachstania_africana_CBS_2517_rna352_F
Saccharomyces_eubayanus_rna3424_F
Saccharomyces_sp_boulardii_rna2564_F
Saccharomyces_cerevisiae_S288C_rna3746_F
Naumovozyma_dairenensis_CBS_421_rna1447_F
Naumovozyma_castellii_CBS_4309_rna2390_F
Candida_dubliniensis_CD36_rna2021_F
Candida_albicans_SC5314_rna1117_F
Lodderomyces_elongisporus_NRRL_YB_4239_rna2550_F
Candida_orthopsilosis_Co_90_125_rna3572_F
Metschnikowia_bicuspidata_var_bicuspidata_NRRL_YB_4993_rna5121_F
Scheffersomyces_stipitis_CBS_6054_rna1012_F
Meyerozyma_guilliermondii_ATCC_6260_rna164_F
Debaryomyces_hansenii_CBS767_rna1512_F
Babjeviella_inositovora_NRRL_Y_12698_rna2037_F
Cyberlindnera_jadinii_NRRL_Y_1542_rna233_F
Komagataella_phaffii_GS115_rna3530_F
Komagataella_pastoris_rna3868_F
Ogataea_parapolymorpha_DL_1_rna4565_F
Pichia_kudriavzevii_rna3647_F
Pichia_membranifaciens_NRRL_Y_2026_rna2564_F
Yarrowia_lipolytica_CLIB122_rna4921_F
Sugiyamaella_lignohabitans_rna2929_F
Schizosaccharomyces_pombe_rna315_F
Jaminaea_rosea_rna2560_F
Ustilago_maydis_521_rna398_F
Kalmanozyma_brasiliensis_GHG001_rna354_F
Malassezia_pachydermatis_rna1928_F
Malassezia_restricta_rna995_F
Blastomyces_dermatitidis_ER_3_rna6643_F
Histoplasma_capsulatum_NAm1_rna5585_F
Allomyces_macrogynus_ATCC_38327_rna1470_T
Bifiguratus_adelaidae_rna921_F
Paramicrosporidium_saccamoebae_rna2847_F
Anncaliia_algerae_PRA339_rna927_F
Encephalitozoon_cuniculi_GB_M1_rna1958_F
Encephalitozoon_intestinalis_ATCC_50506_rna1909_F
Encephalitozoon_romaleae_SJ_2008_rna1792_F
Encephalitozoon_hellem_ATCC_50504_rna1816_F
Diplodia_corticola_rna3708_F
Diplodia_seriata_rna7052_F
Ascosphaera_apis_ARSEF_7405_rna4761_F
Penicillium_chrysogenum_rna5948_F
Aspergillus_fumigatus_Af293_rna9041_F
Aspergillus_calidoustus_rna8043_F
Aspergillus_japonicus_CBS_114_51_rna2744_F
Aspergillus_aculeatinus_CBS_121060_rna4081_F
Aspergillus_aculeatus_ATCC_16872_rna5745_F
Aspergillus_brasiliensis_CBS_101740_rna5557_F
Aspergillus_niger_rna5323_F
Aspergillus_oryzae_RIB40_rna1690_F
Aspergillus_candidus_rna317_F
Exophiala_mesophila_rna1852_T
Exophiala_spinifera_rna6906_F
Capronia_coronata_CBS_61796_rna3773_F
Exophiala_dermatitidis_NIH_UT8656_rna9371_F
0.4
