# Supplementary material for: Animal, Fungi, and Plant Genome Sequences Harbor Different Non-Canonical Splice Sites
Source: Cells. 2020 Feb 18;9(2):458. doi: 10.3390/cells9020458 (PMC7072748; doi:10.3390/cells9020458)
Supplement: Supplementary file 1 [file cells-09-00458-s001.zip › supplements/File S19.zip!/files_for_S19/7350.pdf]

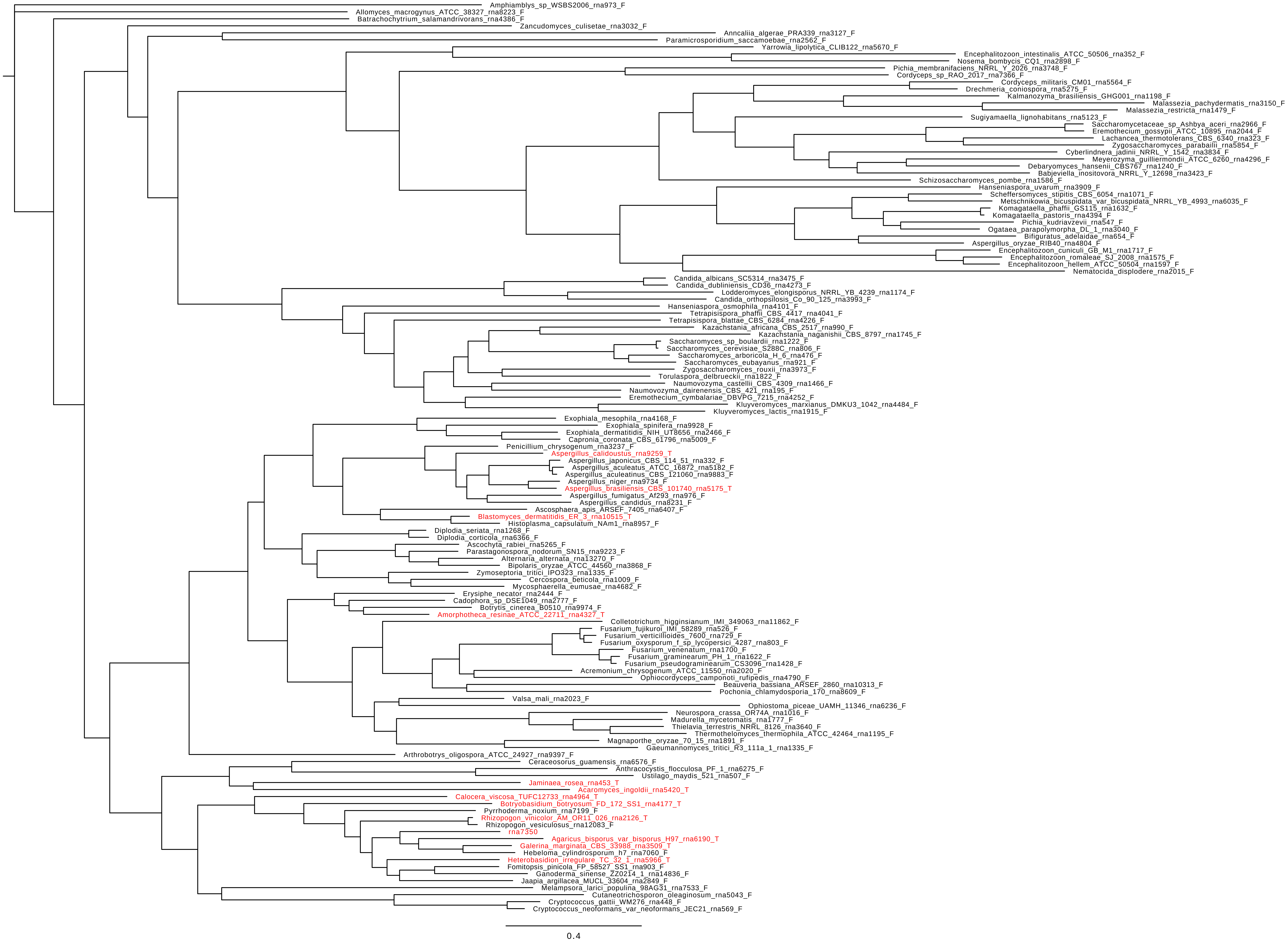
Amphiamblys_sp_WSBS2006_rna973_F
Allomyces_macrogynus_ATCC_38327_rna8223_F
Batrachochytrium_salamandrivorans_rna4386_F
Zancudomyces_culisetae_rna3032_F
Anncaliia_algerae_PRA339_rna3127_F
Paramicrosporidium_saccamoebae_rna2562_F
Yarrowia_lipolytica_CLIB122_rna5670_F
Encephalitozoon_intestinalis_ATCC_50506_rna352_F
Nosema_bombycis_CQ1_rna2898_F
Pichia_membranifaciens_NRRL_Y_2026_rna3748_F
Cordyceps_sp_RAO_2017_rna7366_F
Cordyceps_militaris_CM01_rna5564_F
Drechmeria_coniospora_rna5275_F
Kalmanozyma_brasiliensis_GHG001_rna1198_F
Malassezia_pachydermatis_rna3150_F
Malassezia_restricta_rna1479_F
Sugiyamaella_lignohabitans_rna5123_F
Saccharomycetaceae_sp_Ashbya_aceri_rna2966_F
Eremothecium_gossypii_ATCC_10895_rna2044_F
Lachancea_thermotolerans_CBS_6340_rna323_F
Zygosaccharomyces_parabailii_rna5854_F
Cyberlindnera_jadinii_NRRL_Y_1542_rna3834_F
Meyerozyma_guilliermondii_ATCC_6260_rna4296_F
Debaryomyces_hansenii_CBS767_rna1240_F
Babjeviella_inositovora_NRRL_Y_12698_rna3423_F
Schizosaccharomyces_pombe_rna1586_F
Hanseniaspora_uvarum_rna3909_F
Scheffersomyces_stipitis_CBS_6054_rna1071_F
Metschnikowia_bicuspidata_var_bicuspidata_NRRL_YB_4993_rna6035_F
Komagataella_phaffii_GS115_rna1632_F
Komagataella_pastoris_rna4394_F
Pichia_kudriavzevii_rna547_F
Ogataea_parapolymorpha_DL_1_rna3040_F
Bifiguratus_adelaidae_rna654_F
Aspergillus_oryzae_RIB40_rna4804_F
Encephalitozoon_cuniculi_GB_M1_rna1717_F
Encephalitozoon_romaleae_SJ_2008_rna1575_F
Encephalitozoon_hellem_ATCC_50504_rna1597_F
Nematocida_displodere_rna2015_F
Candida_albicans_SC5314_rna3475_F
Candida_dubliniensis_CD36_rna4273_F
Lodderomyces_elongisporus_NRRL_YB_4239_rna1174_F
Candida_orthopsilosis_Co_90_125_rna3993_F
Hanseniaspora_osmophila_rna4101_F
Tetrapisispora_phaffii_CBS_4417_rna4041_F
Tetrapisispora_blattae_CBS_6284_rna4226_F
Kazachstania_africana_CBS_2517_rna990_F
Kazachstania_naganishii_CBS_8797_rna1745_F
Saccharomyces_sp_boulardii_rna1222_F
Saccharomyces_cerevisiae_S288C_rna806_F
Saccharomyces_arboricola_H_6_rna476_F
Saccharomyces_eubayanus_rna921_F
Zygosaccharomyces_rouxii_rna3973_F
Torulaspora_delbrueckii_rna1822_F
Naumovozyma_castellii_CBS_4309_rna1466_F
Naumovozyma_dairenensis_CBS_421_rna195_F
Eremothecium_cymbalariae_DBVPG_7215_rna4252_F
Kluyveromyces_marxianus_DMKU3_1042_rna4484_F
Kluyveromyces_lactis_rna1915_F
Exophiala_mesophila_rna4168_F
Exophiala_spinifera_rna9928_F
Exophiala_dermatitidis_NIH_UT8656_rna2466_F
Capronia_coronata_CBS_61796_rna5009_F
Penicillium_chrysogenum_rna3237_F
Aspergillus_calidoustus_rna9259_T
Aspergillus_japonicus_CBS_114_51_rna332_F
Aspergillus_aculeatus_ATCC_16872_rna5182_F
Aspergillus_aculeatinus_CBS_121060_rna9883_F
Aspergillus_niger_rna9734_F
Aspergillus_brasiliensis_CBS_101740_rna5175_T
Aspergillus_fumigatus_Af293_rna976_F
Aspergillus_candidus_rna8231_F
Ascosphaera_apis_ARSEF_7405_rna6407_F
Blastomyces_dermatitidis_ER_3_rna10515_T
Histoplasma_capsulatum_NAm1_rna8957_F
Diplodia_seriata_rna1268_F
Diplodia_corticola_rna6366_F
Ascochyta_rabiei_rna5265_F
Parastagonospora_nodorum_SN15_rna9223_F
Alternaria_alternata_rna13270_F
Bipolaris_oryzae_ATCC_44560_rna3868_F
Zymoseptoria_tritici_IPO323_rna1335_F
Cercospora_beticola_rna1009_F
Mycosphaerella_eumusae_rna4682_F
Erysiphe_necator_rna2444_F
Cadophora_sp_DSE1049_rna2777_F
Botrytis_cinerea_B0510_rna9974_F
Amorphotheca_resinae_ATCC_22711_rna4327_T
Colletotrichum_higginsianum_IMI_349063_rna11862_F
Fusarium_fujikuroi_IMI_58289_rna526_F
Fusarium_verticillioides_7600_rna729_F
Fusarium_oxysporum_f_sp_lycopersici_4287_rna803_F
Fusarium_venenatum_rna1700_F
Fusarium_graminearum_PH_1_rna1622_F
Fusarium_pseudograminearum_CS3096_rna1428_F
Acremonium_chrysogenum_ATCC_11550_rna2020_F
Ophiocordyceps_camponoti_rufipedis_rna4790_F
Beauveria_bassiana_ARSEF_2860_rna10313_F
Pochonia_chlamydosporia_170_rna8609_F
Valsa_mali_rna2023_F
Ophiostoma_piceae_UAMH_11346_rna6236_F
Neurospora_crassa_OR74A_rna1016_F
Madurella_mycetomatis_rna1777_F
Thielavia_terrestris_NRRL_8126_rna3640_F
Thermothelomyces_thermophila_ATCC_42464_rna1195_F
Magnaporthe_oryzae_70_15_rna1891_F
Gaeumannomyces_tritici_R3_111a_1_rna1335_F
Arthrobotrys_oligospora_ATCC_24927_rna9397_F
Ceraceosorus_guamensis_rna6576_F
Anthracocystis_flocculosa_PF_1_rna6275_F
Ustilago_maydis_521_rna507_F
Jaminaea_rosea_rna453_T
Acaromyces_ingoldii_rna5420_T
Calocera_viscosa_TUFC12733_rna4964_T
Botryobasidium_botryosum_FD_172_SS1_rna4177_T
Pyrrhoderma_noxium_rna7199_F
Rhizopogon_vinicolor_AM_OR11_026_rna2126_T
Rhizopogon_vesiculosus_rna12083_F
rna7350
Agaricus_bisporus_var_bisporus_H97_rna6190_T
Galerina_marginata_CBS_33988_rna3509_T
Hebeloma_cylindrosporum_h7_rna7060_F
Heterobasidion_irregulare_TC_32_1_rna5966_T
Fomitopsis_pinicola_FP_58527_SS1_rna903_F
Ganoderma_sinense_ZZ0214_1_rna14836_F
Jaapia_argillacea_MUCL_33604_rna2849_F
Melampsora_larici_populina_98AG31_rna7533_F
Cutaneotrichosporon_oleaginosum_rna5043_F
Cryptococcus_gattii_WM276_rna448_F
Cryptococcus_neoformans_var_neoformans_JEC21_rna569_F
0.4
