# Supplementary material for: Animal, Fungi, and Plant Genome Sequences Harbor Different Non-Canonical Splice Sites
Source: Cells. 2020 Feb 18;9(2):458. doi: 10.3390/cells9020458 (PMC7072748; doi:10.3390/cells9020458)
Supplement: Supplementary file 1 [file cells-09-00458-s001.zip › supplements/File S19.zip!/files_for_S19/8171.pdf]

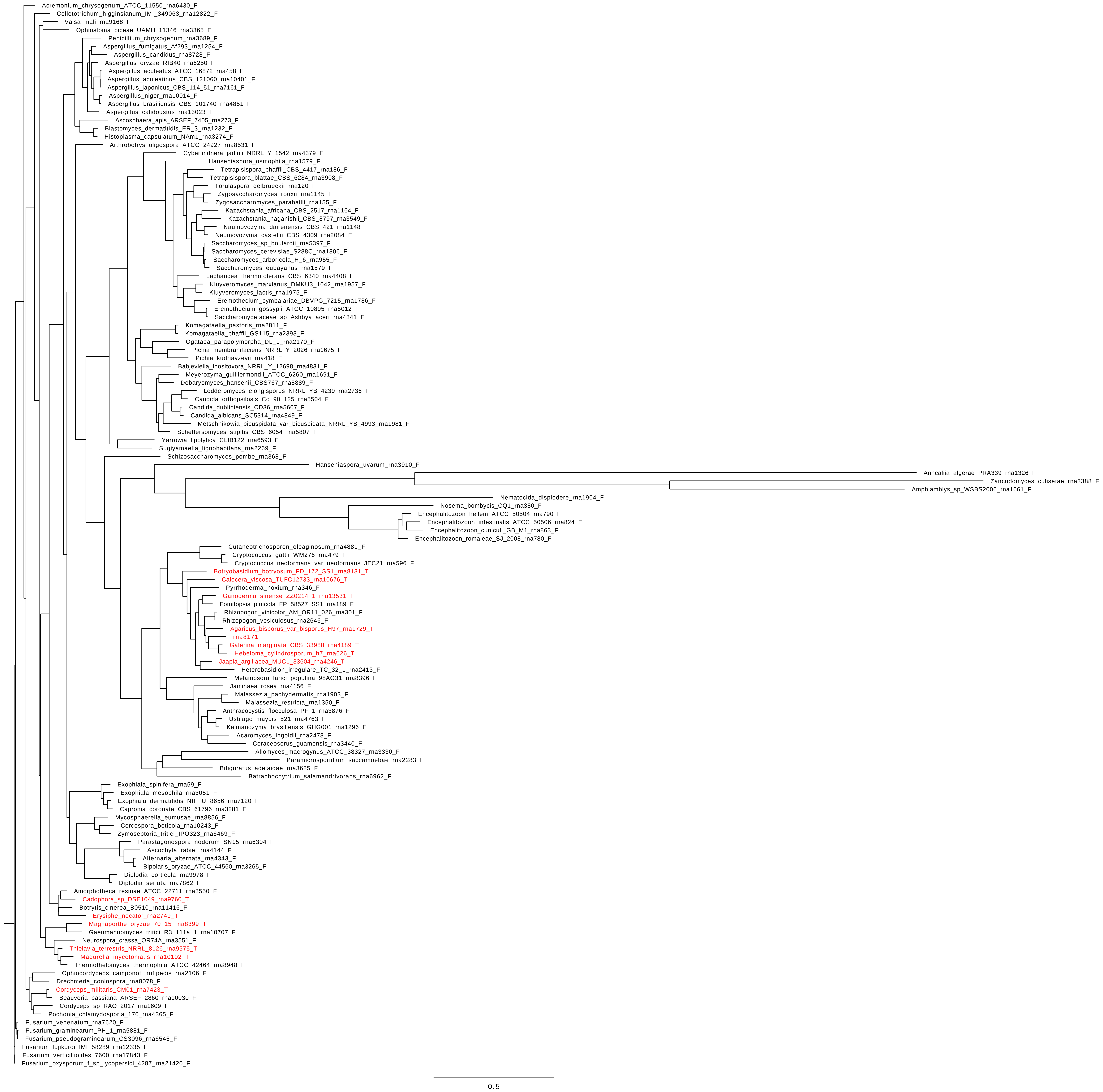
Acremonium_chrysogenum_ATCC_11550_rna6430_F
Colletotrichum_higginsianum_IMI_349063_rna12822_F
Valsa_mali_rna9168_F
Ophiostoma_piceae_UAMH_11346_rna3365_F
Penicillium_chrysogenum_rna3689_F
Aspergillus_fumigatus_Af293_rna1254_F
Aspergillus_candidus_rna8728_F
Aspergillus_oryzae_RIB40_rna6250_F
Aspergillus_aculeatus_ATCC_16872_rna458_F
Aspergillus_aculeatinus_CBS_121060_rna10401_F
Aspergillus_japonicus_CBS_114_51_rna7161_F
Aspergillus_niger_rna10014_F
Aspergillus_brasiliensis_CBS_101740_rna4851_F
Aspergillus_calidoustus_rna13023_F
Ascosphaera_apis_ARSEF_7405_rna273_F
Blastomyces_dermatitidis_ER_3_rna1232_F
Histoplasma_capsulatum_NAm1_rna3274_F
Arthrobotrys_oligospora_ATCC_24927_rna8531_F
Cyberlindnera_jadinii_NRRL_Y_1542_rna4379_F
Hanseniaspora_osmophila_rna1579_F
Tetrapisispora_phaffii_CBS_4417_rna186_F
Tetrapisispora_blattae_CBS_6284_rna3908_F
Torulaspora_delbrueckii_rna120_F
Zygosaccharomyces_rouxii_rna1145_F
Zygosaccharomyces_parabailii_rna155_F
Kazachstania_africana_CBS_2517_rna1164_F
Kazachstania_naganishii_CBS_8797_rna3549_F
Naumovozyma_dairenensis_CBS_421_rna1148_F
Naumovozyma_castellii_CBS_4309_rna2084_F
Saccharomyces_sp_boulardii_rna5397_F
Saccharomyces_cerevisiae_S288C_rna1806_F
Saccharomyces_arboricola_H_6_rna955_F
Saccharomyces_eubayanus_rna1579_F
Lachancea_thermotolerans_CBS_6340_rna4408_F
Kluyveromyces_marxianus_DMKU3_1042_rna1957_F
Kluyveromyces_lactis_rna1975_F
Eremothecium_cymbalariae_DBVPG_7215_rna1786_F
Eremothecium_gossypii_ATCC_10895_rna5012_F
Saccharomycetaceae_sp_Ashbya_aceri_rna4341_F
Komagataella_pastoris_rna2811_F
Komagataella_phaffii_GS115_rna2393_F
Ogataea_parapolymorpha_DL_1_rna2170_F
Pichia_membranifaciens_NRRL_Y_2026_rna1675_F
Pichia_kudriavzevii_rna418_F
Babjeviella_inositovora_NRRL_Y_12698_rna4831_F
Meyerozyma_guilliermondii_ATCC_6260_rna1691_F
Debaryomyces_hansenii_CBS767_rna5889_F
Lodderomyces_elongisporus_NRRL_YB_4239_rna2736_F
Candida_orthopsilosis_Co_90_125_rna5504_F
Candida_dubliniensis_CD36_rna5607_F
Candida_albicans_SC5314_rna4849_F
Metschnikowia_bicuspidata_var_bicuspidata_NRRL_YB_4993_rna1981_F
Scheffersomyces_stipitis_CBS_6054_rna5807_F
Yarrowia_lipolytica_CLIB122_rna6593_F
Sugiyamaella_lignohabitans_rna2269_F
Schizosaccharomyces_pombe_rna368_F
Hanseniaspora_uvarum_rna3910_F
Anncaliia_algerae_PRA339_rna1326_F
Zancudomyces_culisetae_rna3388_F
Amphiamblys_sp_WSBS2006_rna1661_F
Nematocida_displodere_rna1904_F
Nosema_bombycis_CQ1_rna380_F
Encephalitozoon_hellem_ATCC_50504_rna790_F
Encephalitozoon_intestinalis_ATCC_50506_rna824_F
Encephalitozoon_cuniculi_GB_M1_rna863_F
Encephalitozoon_romaleae_SJ_2008_rna780_F
Cutaneotrichosporon_oleaginosum_rna4881_F
Cryptococcus_gattii_WM276_rna479_F
Cryptococcus_neoformans_var_neoformans_JEC21_rna596_F
Botryobasidium_botryosum_FD_172_SS1_rna8131_T
Calocera_viscosa_TUFC12733_rna10676_T
Pyrrhoderma_noxium_rna346_F
Ganoderma_sinense_ZZ0214_1_rna13531_T
Fomitopsis_pinicola_FP_58527_SS1_rna189_F
Rhizopogon_vinicolor_AM_OR11_026_rna301_F
Rhizopogon_vesiculosus_rna2646_F
Agaricus_bisporus_var_bisporus_H97_rna1729_T
rna8171
Galerina_marginata_CBS_33988_rna4189_T
Hebeloma_cylindrosporum_h7_rna626_T
Jaapia_argillacea_MUCL_33604_rna4246_T
Heterobasidion_irregulare_TC_32_1_rna2413_F
Melampsora_larici_populina_98AG31_rna8396_F
Jaminaea_rosea_rna4156_F
Malassezia_pachydermatis_rna1903_F
Malassezia_restricta_rna1350_F
Anthracocystis_flocculosa_PF_1_rna3876_F
Ustilago_maydis_521_rna4763_F
Kalmanozyma_brasiliensis_GHG001_rna1296_F
Acaromyces_ingoldii_rna2478_F
Ceraceosorus_guamensis_rna3440_F
Allomyces_macrogynus_ATCC_38327_rna3330_F
Paramicrosporidium_saccamoebae_rna2283_F
Bifiguratus_adelaidae_rna3625_F
Batrachochytrium_salamandrivorans_rna6962_F
Exophiala_spinifera_rna59_F
Exophiala_mesophila_rna3051_F
Exophiala_dermatitidis_NIH_UT8656_rna7120_F
Capronia_coronata_CBS_61796_rna3281_F
Mycosphaerella_eumusae_rna8856_F
Cercospora_beticola_rna10243_F
Zymoseptoria_tritici_IPO323_rna6469_F
Parastagonospora_nodorum_SN15_rna6304_F
Ascochyta_rabiei_rna4144_F
Alternaria_alternata_rna4343_F
Bipolaris_oryzae_ATCC_44560_rna3265_F
Diplodia_corticola_rna9978_F
Diplodia_seriata_rna7862_F
Amorphotheca_resinae_ATCC_22711_rna3550_F
Cadophora_sp_DSE1049_rna9760_T
Botrytis_cinerea_B0510_rna11416_F
Erysiphe_necator_rna2749_T
Magnaporthe_oryzae_70_15_rna8399_T
Gaeumannomyces_tritici_R3_111a_1_rna10707_F
Neurospora_crassa_OR74A_rna3551_F
Thielavia_terrestris_NRRL_8126_rna9575_T
Madurella_mycetomatis_rna10102_T
Thermothelomyces_thermophila_ATCC_42464_rna8948_F
Ophiocordyceps_camponoti_rufipedis_rna2106_F
Drechmeria_coniospora_rna8078_F
Cordyceps_militaris_CM01_rna7423_T
Beauveria_bassiana_ARSEF_2860_rna10030_F
Cordyceps_sp_RAO_2017_rna1609_F
Pochonia_chlamydosporia_170_rna4365_F
Fusarium_venenatum_rna7620_F
Fusarium_graminearum_PH_1_rna5881_F
Fusarium_pseudograminearum_CS3096_rna6545_F
Fusarium_fujikuroi_IMI_58289_rna12335_F
Fusarium_verticillioides_7600_rna17843_F
Fusarium_oxysporum_f_sp_lycopersici_4287_rna21420_F
0.5
