# Supplementary material for: Animal, Fungi, and Plant Genome Sequences Harbor Different Non-Canonical Splice Sites
Source: Cells. 2020 Feb 18;9(2):458. doi: 10.3390/cells9020458 (PMC7072748; doi:10.3390/cells9020458)
Supplement: Supplementary file 1 [file cells-09-00458-s001.zip › supplements/File S19.zip!/files_for_S19/15438.pdf]

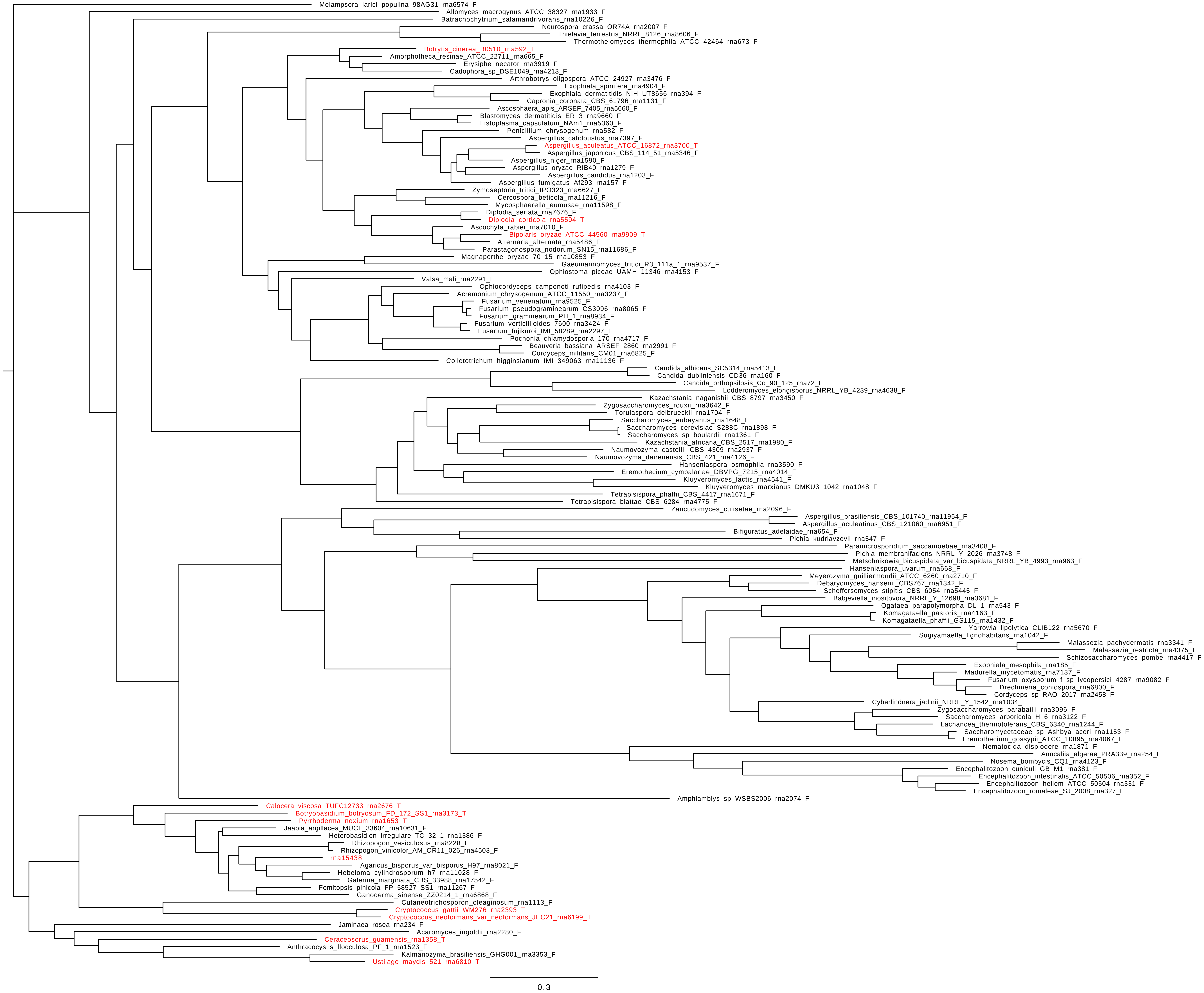
Melampsora_larici_populina_98AG31_rna6574_F
Allomyces_macrogynus_ATCC_38327_rna1933_F
Batrachochytrium_salamandrivorans_rna10226_F
Neurospora_crassa_OR74A_rna2007_F
Thielavia_terrestris_NRRL_8126_rna8606_F
Thermothelomyces_thermophila_ATCC_42464_rna673_F
Botrytis_cinerea_B0510_rna592_T
Amorphotheca_resinae_ATCC_22711_rna665_F
Erysiphe_necator_rna3919_F
Cadophora_sp_DSE1049_rna4213_F
Arthrobotrys_oligospora_ATCC_24927_rna3476_F
Exophiala_spinifera_rna4904_F
Exophiala_dermatitidis_NIH_UT8656_rna394_F
Capronia_coronata_CBS_61796_rna1131_F
Ascosphaera_apis_ARSEF_7405_rna5660_F
Blastomyces_dermatitidis_ER_3_rna9660_F
Histoplasma_capsulatum_NAm1_rna5360_F
Penicillium_chrysogenum_rna582_F
Aspergillus_calidoustus_rna7397_F
Aspergillus_aculeatus_ATCC_16872_rna3700_T
Aspergillus_japonicus_CBS_114_51_rna5346_F
Aspergillus_niger_rna1590_F
Aspergillus_oryzae_RIB40_rna1279_F
Aspergillus_candidus_rna1203_F
Aspergillus_fumigatus_Af293_rna157_F
Zymoseptoria_tritici_IPO323_rna6627_F
Cercospora_beticola_rna11216_F
Mycosphaerella_eumusae_rna11598_F
Diplodia_seriata_rna7676_F
Diplodia_corticola_rna5594_T
Ascochyta_rabiei_rna7010_F
Bipolaris_oryzae_ATCC_44560_rna9909_T
Alternaria_alternata_rna5486_F
Parastagonospora_nodorum_SN15_rna11686_F
Magnaporthe_oryzae_70_15_rna10853_F
Gaeumannomyces_tritici_R3_111a_1_rna9537_F
Ophiostoma_piceae_UAMH_11346_rna4153_F
Valsa_mali_rna2291_F
Ophiocordyceps_camponoti_rufipedis_rna4103_F
Acremonium_chrysogenum_ATCC_11550_rna3237_F
Fusarium_venenatum_rna9525_F
Fusarium_pseudograminearum_CS3096_rna8065_F
Fusarium_graminearum_PH_1_rna8934_F
Fusarium_verticillioides_7600_rna3424_F
Fusarium_fujikuroi_IMI_58289_rna2297_F
Pochonia_chlamydosporia_170_rna4717_F
Beauveria_bassiana_ARSEF_2860_rna2991_F
Cordyceps_militaris_CM01_rna6825_F
Colletotrichum_higginsianum_IMI_349063_rna11136_F
Candida_albicans_SC5314_rna5413_F
Candida_dubliniensis_CD36_rna160_F
Candida_orthopsilosis_Co_90_125_rna72_F
Lodderomyces_elongisporus_NRRL_YB_4239_rna4638_F
Kazachstania_naganishii_CBS_8797_rna3450_F
Zygosaccharomyces_rouxii_rna3642_F
Torulaspora_delbrueckii_rna1704_F
Saccharomyces_eubayanus_rna1648_F
Saccharomyces_cerevisiae_S288C_rna1898_F
Saccharomyces_sp_boulardii_rna1361_F
Kazachstania_africana_CBS_2517_rna1980_F
Naumovozyma_castellii_CBS_4309_rna2937_F
Naumovozyma_dairenensis_CBS_421_rna4126_F
Hanseniaspora_osmophila_rna3590_F
Eremothecium_cymbalariae_DBVPG_7215_rna4014_F
Kluyveromyces_lactis_rna4541_F
Kluyveromyces_marxianus_DMKU3_1042_rna1048_F
Tetrapisispora_phaffii_CBS_4417_rna1671_F
Tetrapisispora_blattae_CBS_6284_rna4775_F
Zancudomyces_culisetae_rna2096_F
Aspergillus_brasiliensis_CBS_101740_rna11954_F
Aspergillus_aculeatinus_CBS_121060_rna6951_F
Bifiguratus_adelaidae_rna654_F
Pichia_kudriavzevii_rna547_F
Paramicrosporidium_saccamoebae_rna3408_F
Pichia_membranifaciens_NRRL_Y_2026_rna3748_F
Metschnikowia_bicuspidata_var_bicuspidata_NRRL_YB_4993_rna963_F
Hanseniaspora_uvarum_rna668_F
Meyerozyma_guilliermondii_ATCC_6260_rna2710_F
Debaryomyces_hansenii_CBS767_rna1342_F
Scheffersomyces_stipitis_CBS_6054_rna5445_F
Babjeviella_inositovora_NRRL_Y_12698_rna3681_F
Ogataea_parapolymorpha_DL_1_rna543_F
Komagataella_pastoris_rna4163_F
Komagataella_phaffii_GS115_rna1432_F
Yarrowia_lipolytica_CLIB122_rna5670_F
Sugiyamaella_lignohabitans_rna1042_F
Malassezia_pachydermatis_rna3341_F
Malassezia_restricta_rna4375_F
Schizosaccharomyces_pombe_rna4417_F
Exophiala_mesophila_rna185_F
Madurella_mycetomatis_rna7137_F
Fusarium_oxysporum_f_sp_lycopersici_4287_rna9082_F
Drechmeria_coniospora_rna6800_F
Cordyceps_sp_RAO_2017_rna2458_F
Cyberlindnera_jadinii_NRRL_Y_1542_rna1034_F
Zygosaccharomyces_parabailii_rna3096_F
Saccharomyces_arboricola_H_6_rna3122_F
Lachancea_thermotolerans_CBS_6340_rna1244_F
Saccharomycetaceae_sp_Ashbya_aceri_rna1153_F
Eremothecium_gossypii_ATCC_10895_rna4067_F
Nematocida_displodere_rna1871_F
Anncaliia_algerae_PRA339_rna254_F
Nosema_bombycis_CQ1_rna4123_F
Encephalitozoon_cuniculi_GB_M1_rna381_F
Encephalitozoon_intestinalis_ATCC_50506_rna352_F
Encephalitozoon_hellem_ATCC_50504_rna331_F
Encephalitozoon_romaleae_SJ_2008_rna327_F
Amphiamblys_sp_WSBS2006_rna2074_F
Calocera_viscosa_TUFC12733_rna2676_T
Botryobasidium_botryosum_FD_172_SS1_rna3173_T
Pyrrhoderma_noxium_rna1653_T
Jaapia_argillacea_MUCL_33604_rna10631_F
Heterobasidion_irregulare_TC_32_1_rna1386_F
Rhizopogon_vesiculosus_rna8228_F
Rhizopogon_vinicolor_AM_OR11_026_rna4503_F
rna15438
Agaricus_bisporus_var_bisporus_H97_rna8021_F
Hebeloma_cylindrosporum_h7_rna11028_F
Galerina_marginata_CBS_33988_rna17542_F
Fomitopsis_pinicola_FP_58527_SS1_rna11267_F
Ganoderma_sinense_ZZ0214_1_rna6868_F
Cutaneotrichosporon_oleaginosum_rna1113_F
Cryptococcus_gattii_WM276_rna2393_T
Cryptococcus_neoformans_var_neoformans_JEC21_rna6199_T
Jaminaea_rosea_rna234_F
Acaromyces_ingoldii_rna2280_F
Ceraceosorus_guamensis_rna1358_T
Anthracocystis_flocculosa_PF_1_rna1523_F
Kalmanozyma_brasiliensis_GHG001_rna3353_F
Ustilago_maydis_521_rna6810_T
0.3
