# Supplementary material for: Animal, Fungi, and Plant Genome Sequences Harbor Different Non-Canonical Splice Sites
Source: Cells. 2020 Feb 18;9(2):458. doi: 10.3390/cells9020458 (PMC7072748; doi:10.3390/cells9020458)
Supplement: Supplementary file 1 [file cells-09-00458-s001.zip › supplements/File S19.zip!/files_for_S19/16048.pdf]

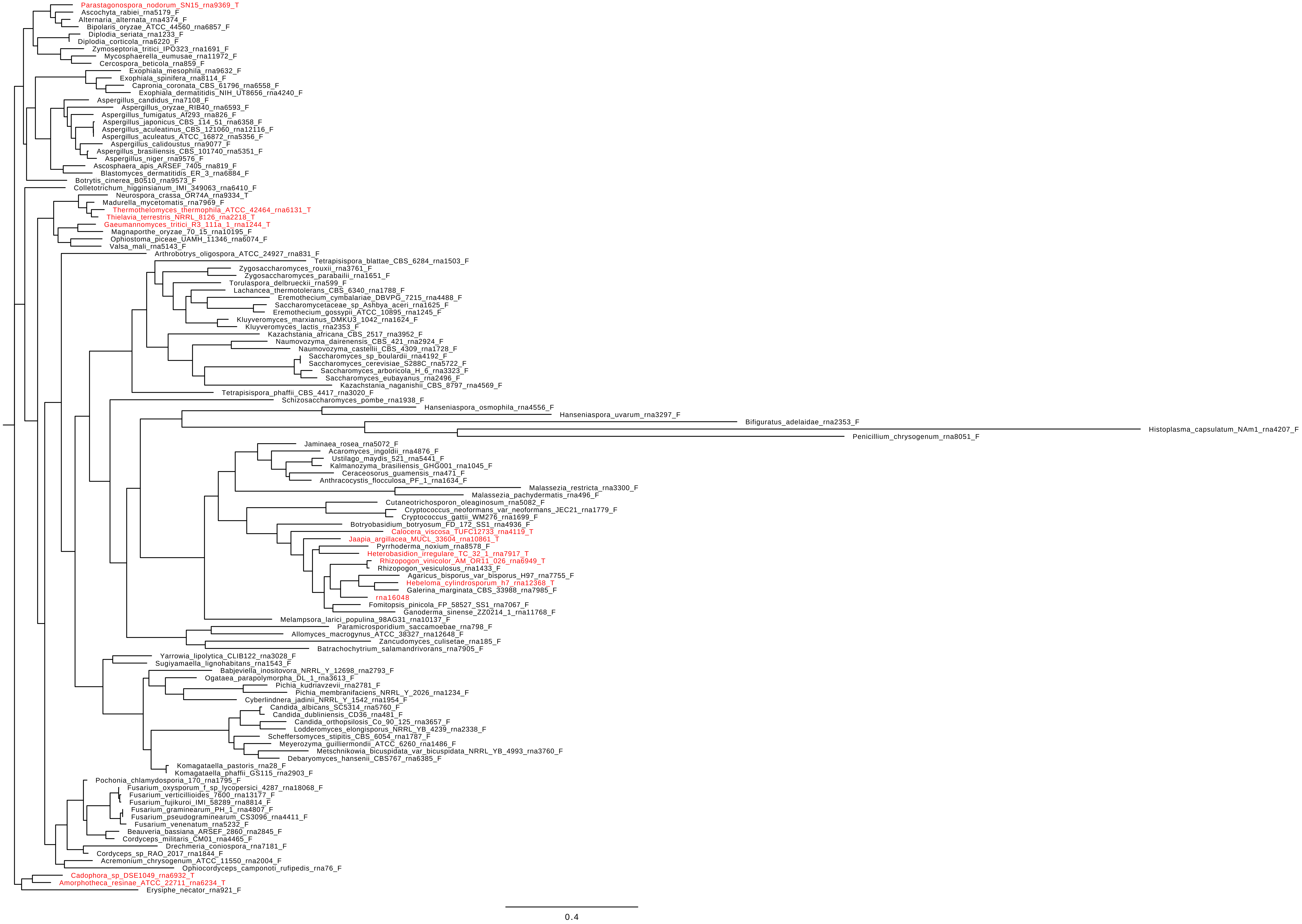

Parastagonospora_nodorum_SN15_rna9369_T
Ascochyta_rabiei_rna5179_F
Alternaria_alternata_rna4374_F
Bipolaris_oryzae_ATCC_44560_rna6857_F
Diplodia_seriata_rna1233_F
Diplodia_corticola_rna6220_F
Zymoseptoria_tritici_IPO323_rna1691_F
Mycosphaerella_eumusae_rna11972_F
Cercospora_beticola_rna859_F
Exophiala_mesophila_rna9632_F
Exophiala_spinifera_rna8114_F
Capronia_coronata_CBS_61796_rna6558_F
Exophiala_dermatitidis_NIH_UT8656_rna4240_F
Aspergillus_candidus_rna7108_F
Aspergillus_oryzae_RIB40_rna6593_F
Aspergillus_fumigatus_Af293_rna826_F
Aspergillus_japonicus_CBS_114_51_rna6358_F
Aspergillus_aculeatinus_CBS_121060_rna12116_F
Aspergillus_aculeatus_ATCC_16872_rna5356_F
Aspergillus_calidoustus_rna9077_F
Aspergillus_brasiliensis_CBS_101740_rna5351_F
Aspergillus_niger_rna9576_F
Ascosphaera_apis_ARSEF_7405_rna819_F
Blastomyces_dermatitidis_ER_3_rna6884_F
Botrytis_cinerea_B0510_rna9573_F
Colletotrichum_higginsianum_IMI_349063_rna6410_F
Neurospora_crassa_OR74A_rna9334_T
Madurella_mycetomatis_rna7969_F
Thermothelomyces_thermophila_ATCC_42464_rna6131_T
Thielavia_terrestris_NRRL_8126_rna2218_T
Gaeumannomyces_tritici_R3_111a_1_rna1244_T
Magnaporthe_oryzae_70_15_rna10195_F
Ophiostoma_piceae_UAMH_11346_rna6074_F
Valsa_mali_rna5143_F
Arthrobotrys_oligospora_ATCC_24927_rna831_F
Tetrapisispora_blattae_CBS_6284_rna1503_F
Zygosaccharomyces_rouxii_rna3761_F
Zygosaccharomyces_parabailii_rna1651_F
Torulaspora_delbrueckii_rna599_F
Lachancea_thermotolerans_CBS_6340_rna1788_F
Eremothecium_cymbalariae_DBVPG_7215_rna4488_F
Saccharomycetaceae_sp_Ashbya_aceri_rna1625_F
Eremothecium_gossypii_ATCC_10895_rna1245_F
Kluyveromyces_marxianus_DMKU3_1042_rna1624_F
Kluyveromyces_lactis_rna2353_F
Kazachstania_africana_CBS_2517_rna3952_F
Naumovozyma_dairenensis_CBS_421_rna2924_F
Naumovozyma_castellii_CBS_4309_rna1728_F
Saccharomyces_sp_boulardii_rna4192_F
Saccharomyces_cerevisiae_S288C_rna5722_F
Saccharomyces_arboricola_H_6_rna3323_F
Saccharomyces_eubayanus_rna2496_F
Kazachstania_naganishii_CBS_8797_rna4569_F
Tetrapisispora_phaffii_CBS_4417_rna3020_F
Schizosaccharomyces_pombe_rna1938_F
Hanseniaspora_osmophila_rna4556_F
Hanseniaspora_uvarum_rna3297_F
Bifiguratus_adelaidae_rna2353_F
Histoplasma_capsulatum_NAm1_rna4207_F
Penicillium_chrysogenum_rna8051_F
Jaminaea_rosea_rna5072_F
Acaromyces_ingoldii_rna4876_F
Ustilago_maydis_521_rna5441_F
Kalmanozyma_brasiliensis_GHG001_rna1045_F
Ceraceosorus_guamensis_rna471_F
Anthracocystis_flocculosa_PF_1_rna1634_F
Malassezia_restricta_rna3300_F
Malassezia_pachydermatis_rna496_F
Cutaneotrichosporon_oleaginosum_rna5082_F
Cryptococcus_neoformans_var_neoformans_JEC21_rna1779_F
Cryptococcus_gattii_WM276_rna1699_F
Botryobasidium_botryosum_FD_172_SS1_rna4936_F
Calocera_viscosa_TUFC12733_rna4119_T
Jaapia_argillacea_MUCL_33604_rna10861_T
Pyrrhoderma_noxium_rna8578_F
Heterobasidion_irregulare_TC_32_1_rna7917_T
Rhizopogon_vinicolor_AM_OR11_026_rna6949_T
Rhizopogon_vesiculosus_rna1433_F
Agaricus_bisporus_var_bisporus_H97_rna7755_F
Hebeloma_cylindrosporum_h7_rna12368_T
Galerina_marginata_CBS_33988_rna7985_F
rna16048
Fomitopsis_pinicola_FP_58527_SS1_rna7067_F
Ganoderma_sinense_ZZ0214_1_rna11768_F
Melampsora_larici_populina_98AG31_rna10137_F
Paramicrosporidium_saccamoebae_rna798_F
Allomyces_macrogynus_ATCC_38327_rna12648_F
Zancudomyces_culisetae_rna185_F
Batrachochytrium_salamandrivorans_rna7905_F
Yarrowia_lipolytica_CLIB122_rna3028_F
Sugiyamaella_lignohabitans_rna1543_F
Babjeviella_inositovora_NRRL_Y_12698_rna2793_F
Ogataea_parapolymorpha_DL_1_rna3613_F
Pichia_kudriavzevii_rna2781_F
Pichia_membranifaciens_NRRL_Y_2026_rna1234_F
Cyberlindnera_jadinii_NRRL_Y_1542_rna1954_F
Candida_albicans_SC5314_rna5760_F
Candida_dubliniensis_CD36_rna481_F
Candida_orthopsilosis_Co_90_125_rna3657_F
Lodderomyces_elongisporus_NRRL_YB_4239_rna2338_F
Scheffersomyces_stipitis_CBS_6054_rna1787_F
Meyerozyma_guilliermondii_ATCC_6260_rna1486_F
Metschnikowia_bicuspidata_var_bicuspidata_NRRL_YB_4993_rna3760_F
Debaryomyces_hansenii_CBS767_rna6385_F
Komagataella_pastoris_rna28_F
Komagataella_phaffii_GS115_rna2903_F
Pochonia_chlamydosporia_170_rna1795_F
Fusarium_oxysporum_f_sp_lycopersici_4287_rna18068_F
Fusarium_verticillioides_7600_rna13177_F
Fusarium_fujikuroi_IMI_58289_rna8814_F
Fusarium_graminearum_PH_1_rna4807_F
Fusarium_pseudograminearum_CS3096_rna4411_F
Fusarium_venenatum_rna5232_F
Beauveria_bassiana_ARSEF_2860_rna2845_F
Cordyceps_militaris_CM01_rna4465_F
Drechmeria_coniospora_rna7181_F
Cordyceps_sp_RAO_2017_rna1844_F
Acremonium_chrysogenum_ATCC_11550_rna2004_F
Ophiocordyceps_camponoti_rufipedis_rna76_F
Cadophora_sp_DSE1049_rna6932_T
Amorphotheca_resinae_ATCC_22711_rna6234_T
Erysiphe_necator_rna921_F
0.4
