# Supplementary material for: Animal, Fungi, and Plant Genome Sequences Harbor Different Non-Canonical Splice Sites
Source: Cells. 2020 Feb 18;9(2):458. doi: 10.3390/cells9020458 (PMC7072748; doi:10.3390/cells9020458)
Supplement: Supplementary file 1 [file cells-09-00458-s001.zip › supplements/File S19.zip!/files_for_S19/17492.pdf]

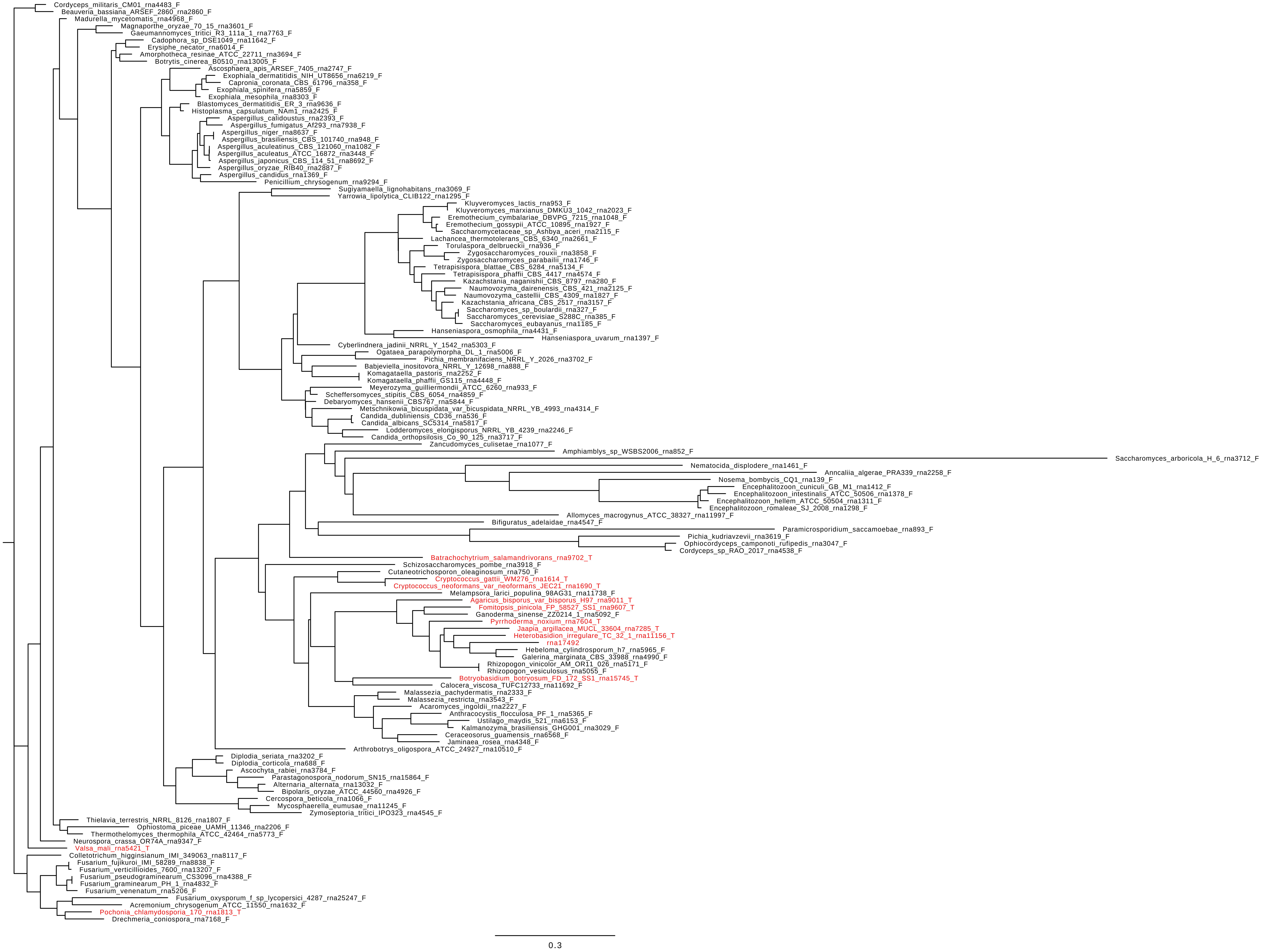

Cordyceps_militaris_CM01_rna4483_F
Beauveria_bassiana_ARSEF_2860_rna2860_F
Madurella_mycetomatis_rna4968_F
Magnaporthe_oryzae_70_15_rna3601_F
Gaeumannomyces_tritici_R3_111a_1_rna7763_F
Cadophora_sp_DSE1049_rna11642_F
Erysiphe_necator_rna6014_F
Amorphotheca_resinae_ATCC_22711_rna3694_F
Botrytis_cinerea_B0510_rna13005_F
Ascosphaera_apis_ARSEF_7405_rna2747_F
Exophiala_dermatitidis_NIH_UT8656_rna6219_F
Capronia_coronata_CBS_61796_rna358_F
Exophiala_spinifera_rna5859_F
Exophiala_mesophila_rna8303_F
Blastomyces_dermatitidis_ER_3_rna9636_F
Histoplasma_capsulatum_NAm1_rna2425_F
Aspergillus_calidoustus_rna2393_F
Aspergillus_fumigatus_Af293_rna7938_F
Aspergillus_niger_rna8637_F
Aspergillus_brasiliensis_CBS_101740_rna948_F
Aspergillus_aculeatinus_CBS_121060_rna1082_F
Aspergillus_aculeatus_ATCC_16872_rna3448_F
Aspergillus_japonicus_CBS_114_51_rna8692_F
Aspergillus_oryzae_RIB40_rna2887_F
Aspergillus_candidus_rna1369_F
Penicillium_chrysogenum_rna9294_F
Sugiyamaella_lignohabitans_rna3069_F
Yarrowia_lipolytica_CLIB122_rna1295_F
Kluyveromyces_lactis_rna953_F
Kluyveromyces_marxianus_DMKU3_1042_rna2023_F
Eremothecium_cymbalariae_DBVPG_7215_rna1048_F
Eremothecium_gossypii_ATCC_10895_rna1927_F
Saccharomycetaceae_sp_Ashbya_aceri_rna2115_F
Lachancea_thermotolerans_CBS_6340_rna2661_F
Torulaspora_delbrueckii_rna936_F
Zygosaccharomyces_rouxii_rna3858_F
Zygosaccharomyces_parabailii_rna1746_F
Tetrapisispora_blattae_CBS_6284_rna5134_F
Tetrapisispora_phaffii_CBS_4417_rna4574_F
Kazachstania_naganishii_CBS_8797_rna280_F
Naumovozyma_dairenensis_CBS_421_rna2125_F
Naumovozyma_castellii_CBS_4309_rna1827_F
Kazachstania_africana_CBS_2517_rna3157_F
Saccharomyces_sp_boulardii_rna327_F
Saccharomyces_cerevisiae_S288C_rna385_F
Saccharomyces_eubayanus_rna1185_F
Hanseniaspora_osmophila_rna4431_F
Hanseniaspora_uvarum_rna1397_F
Cyberlindnera_jadinii_NRRL_Y_1542_rna5303_F
Ogataea_parapolymorpha_DL_1_rna5006_F
Pichia_membranifaciens_NRRL_Y_2026_rna3702_F
Babjeviella_inositovora_NRRL_Y_12698_rna888_F
Komagataella_pastoris_rna2252_F
Komagataella_phaffii_GS115_rna4448_F
Meyerozyma_guilliermondii_ATCC_6260_rna933_F
Scheffersomyces_stipitis_CBS_6054_rna4859_F
Debaryomyces_hansenii_CBS767_rna5844_F
Metschnikowia_bicuspidata_var_bicuspidata_NRRL_YB_4993_rna4314_F
Candida_dubliniensis_CD36_rna536_F
Candida_albicans_SC5314_rna5817_F
Lodderomyces_elongisporus_NRRL_YB_4239_rna2246_F
Candida_orthopsilosis_Co_90_125_rna3717_F
Zancudomyces_culisetae_rna1077_F
Amphiamblys_sp_WSBS2006_rna852_F
Saccharomyces_arboricola_H_6_rna3712_F
Nematocida_displodere_rna1461_F
Anncaliia_algerae_PRA339_rna2258_F
Nosema_bombycis_CQ1_rna139_F
Encephalitozoon_cuniculi_GB_M1_rna1412_F
Encephalitozoon_intestinalis_ATCC_50506_rna1378_F
Encephalitozoon_hellem_ATCC_50504_rna1311_F
Encephalitozoon_romaleae_SJ_2008_rna1298_F
Allomyces_macrogynus_ATCC_38327_rna11997_F
Bifiguratus_adelaidae_rna4547_F
Paramicrosporidium_saccamoebae_rna893_F
Pichia_kudriavzevii_rna3619_F
Ophiocordyceps_camponoti_rufipedis_rna3047_F
Cordyceps_sp_RAO_2017_rna4538_F
Batrachochytrium_salamandrivorans_rna9702_T
Schizosaccharomyces_pombe_rna3918_F
Cutaneotrichosporon_oleaginosum_rna750_F
Cryptococcus_gattii_WM276_rna1614_T
Cryptococcus_neoformans_var_neoformans_JEC21_rna1690_T
Melampsora_larici_populina_98AG31_rna11738_F
Agaricus_bisporus_var_bisporus_H97_rna9011_T
Fomitopsis_pinicola_FP_58527_SS1_rna9607_T
Ganoderma_sinense_ZZ0214_1_rna5092_F
Pyrrhoderma_noxium_rna7604_T
Jaapia_argillacea_MUCL_33604_rna7285_T
Heterobasidion_irregulare_TC_32_1_rna11156_T
rna17492
Hebeloma_cylindrosporum_h7_rna5965_F
Galerina_marginata_CBS_33988_rna4990_F
Rhizopogon_vinicolor_AM_OR11_026_rna5171_F
Rhizopogon_vesiculosus_rna5055_F
Botryobasidium_botryosum_FD_172_SS1_rna15745_T
Calocera_viscosa_TUFC12733_rna11692_F
Malassezia_pachydermatis_rna2333_F
Malassezia_restricta_rna3543_F
Acaromyces_ingoldii_rna2227_F
Anthracocystis_flocculosa_PF_1_rna5365_F
Ustilago_maydis_521_rna6153_F
Kalmanozyma_brasiliensis_GHG001_rna3029_F
Ceraceosorus_guamensis_rna6568_F
Jaminaea_rosea_rna4348_F
Arthrobotrys_oligospora_ATCC_24927_rna10510_F
Diplodia_seriata_rna3202_F
Diplodia_corticola_rna688_F
Ascochyta_rabiei_rna3784_F
Parastagonospora_nodorum_SN15_rna15864_F
Alternaria_alternata_rna13032_F
Bipolaris_oryzae_ATCC_44560_rna4926_F
Cercospora_beticola_rna1066_F
Mycosphaerella_eumusae_rna11245_F
Zymoseptoria_tritici_IPO323_rna4545_F
Thielavia_terrestris_NRRL_8126_rna1807_F
Ophiostoma_piceae_UAMH_11346_rna2206_F
Thermothelomyces_thermophila_ATCC_42464_rna5773_F
Neurospora_crassa_OR74A_rna9347_F
Valsa_mali_rna5421_T
Colletotrichum_higginsianum_IMI_349063_rna8117_F
Fusarium_fujikuroi_IMI_58289_rna8838_F
Fusarium_verticillioides_7600_rna13207_F
Fusarium_pseudograminearum_CS3096_rna4388_F
Fusarium_graminearum_PH_1_rna4832_F
Fusarium_venenatum_rna5206_F
Fusarium_oxysporum_f_sp_lycopersici_4287_rna25247_F
Acremonium_chrysogenum_ATCC_11550_rna1632_F
Pochonia_chlamydosporia_170_rna1813_T
Drechmeria_coniospora_rna7168_F
0.3
