# Supplementary material for: Animal, Fungi, and Plant Genome Sequences Harbor Different Non-Canonical Splice Sites
Source: Cells. 2020 Feb 18;9(2):458. doi: 10.3390/cells9020458 (PMC7072748; doi:10.3390/cells9020458)
Supplement: Supplementary file 1 [file cells-09-00458-s001.zip › supplements/File S19.zip!/files_for_S19/20034.pdf]

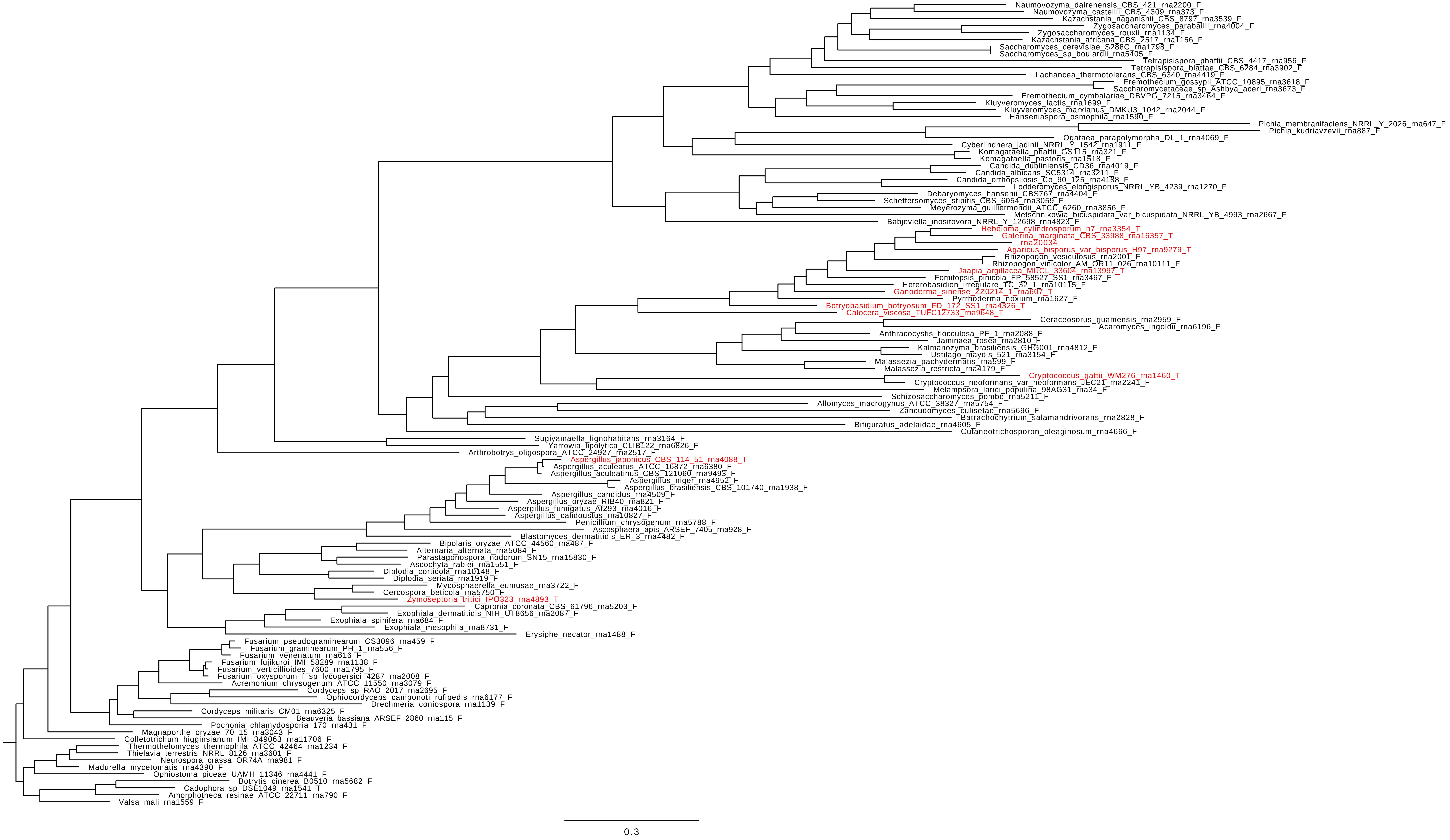

Naumovozyma_dairenensis_CBS_421_rna2200_F
Naumovozyma_castellii_CBS_4309_rna373_F
Kazachstania_naganishii_CBS_8797_rna3539_F
Zygosaccharomyces_parabailii_rna4004_F
Zygosaccharomyces_rouxii_rna1134_F
Kazachstania_africana_CBS_2517_rna1156_F
Saccharomyces_cerevisiae_S288C_rna1798_F
Saccharomyces_sp_boulardii_rna5405_F
Tetrapisispora_phaffii_CBS_4417_rna956_F
Tetrapisispora_blattae_CBS_6284_rna3902_F
Lachancea_thermotolerans_CBS_6340_rna4419_F
Eremothecium_gossypii_ATCC_10895_rna3618_F
Saccharomycetaceae_sp_Ashbya_aceri_rna3673_F
Eremothecium_cymbalariae_DBVPG_7215_rna3464_F
Kluyveromyces_lactis_rna1699_F
Kluyveromyces_marxianus_DMKU3_1042_rna2044_F
Hanseniaspora_osmophila_rna1590_F
Pichia_membranifaciens_NRRL_Y_2026_rna647_F
Pichia_kudriavzevii_rna887_F
Ogataea_parapolymorpha_DL_1_rna4069_F
Cyberlindnera_jadinii_NRRL_Y_1542_rna1911_F
Komagataella_phaffii_GS115_rna321_F
Komagataella_pastoris_rna1518_F
Candida_dubliniensis_CD36_rna4019_F
Candida_albicans_SC5314_rna3211_F
Candida_orthopsilosis_Co_90_125_rna4188_F
Lodderomyces_elongisporus_NRRL_YB_4239_rna1270_F
Debaryomyces_hansenii_CBS767_rna4404_F
Scheffersomyces_stipitis_CBS_6054_rna3059_F
Meyerozyma_guilliermondii_ATCC_6260_rna3856_F
Metschnikowia_bicuspidata_var_bicuspidata_NRRL_YB_4993_rna2667_F
Babjeviella_inositovora_NRRL_Y_12698_rna4823_F
Hebeloma_cylindrosporum_h7_rna3354_T
Galerina_marginata_CBS_33988_rna16357_T
rna20034
Agaricus_bisporus_var_bisporus_H97_rna9279_T
Rhizopogon_vesiculosus_rna2001_F
Rhizopogon_vinicolor_AM_OR11_026_rna10111_F
Jaapia_argillacea_MUCL_33604_rna13997_T
Fomitopsis_pinicola_FP_58527_SS1_rna3467_F
Heterobasidion_irregulare_TC_32_1_rna10115_F
Ganoderma_sinense_ZZ0214_1_rna607_T
Pyrrhoderma_noxium_rna1627_F
Botryobasidium_botryosum_FD_172_SS1_rna4326_T
Calocera_viscosa_TUFC12733_rna9648_T
Ceraceosorus_guamensis_rna2959_F
Acaromyces_ingoldii_rna6196_F
Anthracocystis_flocculosa_PF_1_rna2088_F
Jaminaea_rosea_rna2810_F
Kalmanozyma_brasiliensis_GHG001_rna4812_F
Ustilago_maydis_521_rna3154_F
Malassezia_pachydermatis_rna599_F
Malassezia_restricta_rna4179_F
Cryptococcus_gattii_WM276_rna1460_T
Cryptococcus_neoformans_var_neoformans_JEC21_rna2241_F
Melampsora_larici_populina_98AG31_rna34_F
Schizosaccharomyces_pombe_rna5211_F
Allomyces_macrogynus_ATCC_38327_rna5754_F
Zancudomyces_culisetae_rna5696_F
Batrachochytrium_salamandrivorans_rna2828_F
Bifiguratus_adelaidae_rna4605_F
Cutaneotrichosporon_oleaginosum_rna4666_F
Sugiyamaella_lignohabitans_rna3164_F
Yarrowia_lipolytica_CLIB122_rna6826_F
Arthrobotrys_oligospora_ATCC_24927_rna2517_F
Aspergillus_japonicus_CBS_114_51_rna4088_T
Aspergillus_aculeatus_ATCC_16872_rna6380_F
Aspergillus_aculeatinus_CBS_121060_rna9493_F
Aspergillus_niger_rna4952_F
Aspergillus_brasiliensis_CBS_101740_rna1938_F
Aspergillus_candidus_rna4509_F
Aspergillus_oryzae_RIB40_rna821_F
Aspergillus_fumigatus_Af293_rna4016_F
Aspergillus_calidoustus_rna10827_F
Penicillium_chrysogenum_rna5788_F
Ascosphaera_apis_ARSEF_7405_rna928_F
Blastomyces_dermatitidis_ER_3_rna4482_F
Bipolaris_oryzae_ATCC_44560_rna487_F
Alternaria_alternata_rna5084_F
Parastagonospora_nodorum_SN15_rna15830_F
Ascochyta_rabiei_rna1551_F
Diplodia_corticola_rna10148_F
Diplodia_seriata_rna1919_F
Mycosphaerella_eumusae_rna3722_F
Cercospora_beticola_rna5750_F
Zymoseptoria_tritici_IPO323_rna4893_T
Capronia_coronata_CBS_61796_rna5203_F
Exophiala_dermatitidis_NIH_UT8656_rna2087_F
Exophiala_spinifera_rna684_F
Exophiala_mesophila_rna8731_F
Erysiphe_necator_rna1488_F
Fusarium_pseudograminearum_CS3096_rna459_F
Fusarium_graminearum_PH_1_rna556_F
Fusarium_venenatum_rna616_F
Fusarium_fujikuroi_IMI_58289_rna1138_F
Fusarium_verticillioides_7600_rna1795_F
Fusarium_oxysporum_f_sp_lycopersici_4287_rna2008_F
Acremonium_chrysogenum_ATCC_11550_rna3079_F
Cordyceps_sp_RAO_2017_rna2695_F
Ophiocordyceps_camponoti_rufipedis_rna6177_F
Drechmeria_coniospora_rna1139_F
Cordyceps_militaris_CM01_rna6325_F
Beauveria_bassiana_ARSEF_2860_rna115_F
Pochonia_chlamydosporia_170_rna431_F
Magnaporthe_oryzae_70_15_rna3043_F
Colletotrichum_higginsianum_IMI_349063_rna11706_F
Thermothelomyces_thermophila_ATCC_42464_rna1234_F
Thielavia_terrestris_NRRL_8126_rna3601_F
Neurospora_crassa_OR74A_rna981_F
Madurella_mycetomatis_rna4390_F
Ophiostoma_piceae_UAMH_11346_rna4441_F
Botrytis_cinerea_B0510_rna5682_F
Cadophora_sp_DSE1049_rna1541_T
Amorphotheca_resinae_ATCC_22711_rna790_F
Valsa_mali_rna1559_F
0.3
