# Supplementary material for: Animal, Fungi, and Plant Genome Sequences Harbor Different Non-Canonical Splice Sites
Source: Cells. 2020 Feb 18;9(2):458. doi: 10.3390/cells9020458 (PMC7072748; doi:10.3390/cells9020458)
Supplement: Supplementary file 1 [file cells-09-00458-s001.zip › supplements/File S19.zip!/files_for_S19/22308.pdf]

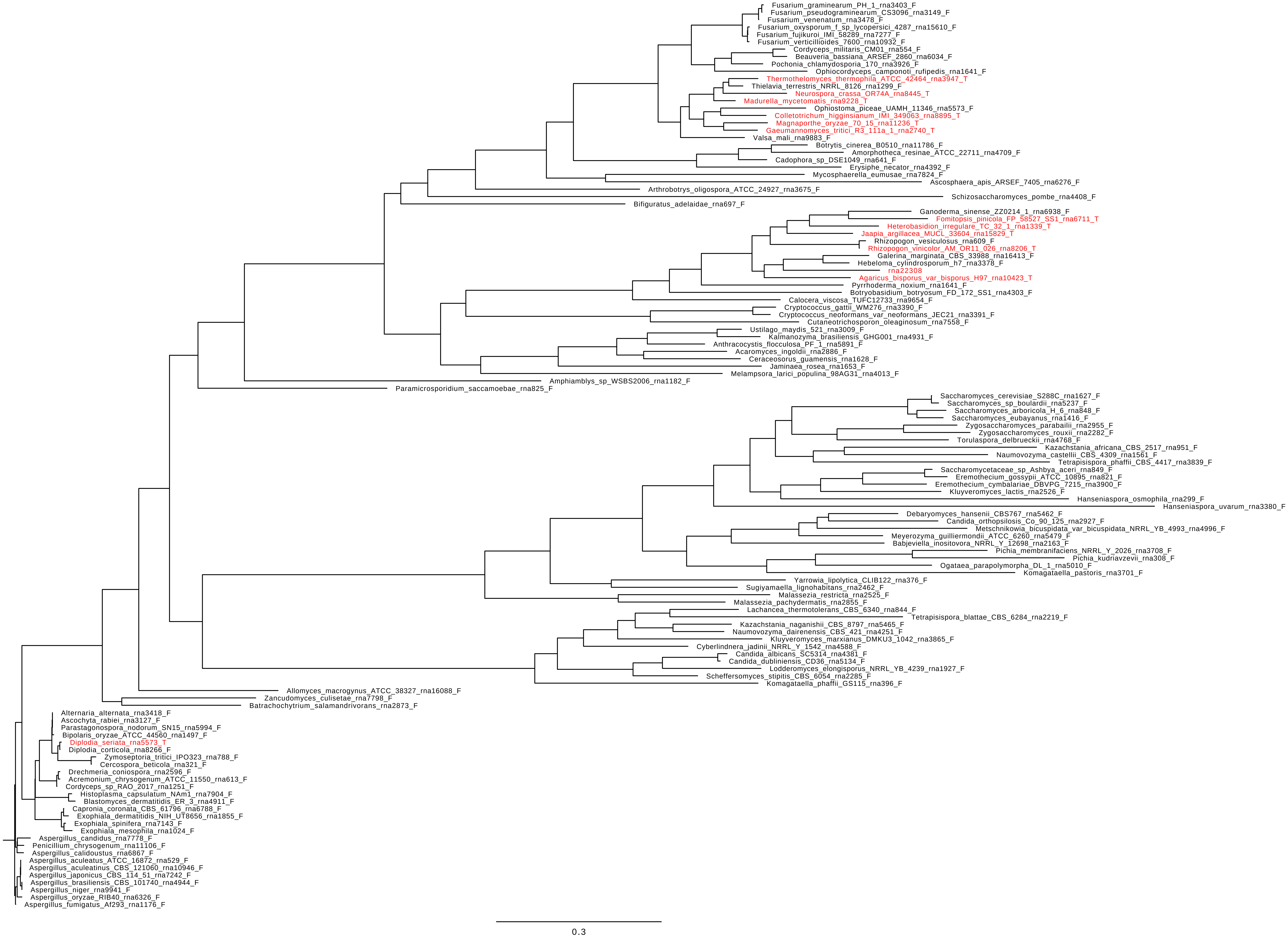
Fusarium_graminearum_PH_1_rna3403_F
Fusarium_pseudograminearum_CS3096_rna3149_F
Fusarium_venenatum_rna3478_F
Fusarium_oxysporum_f_sp_lycopersici_4287_rna15610_F
Fusarium_fujikuroi_IMI_58289_rna7277_F
Fusarium_verticillioides_7600_rna10932_F
Cordyceps_militaris_CM01_rna554_F
Beauveria_bassiana_ARSEF_2860_rna6034_F
Pochonia_chlamydosporia_170_rna3926_F
Ophiocordyceps_camponoti_rufipedis_rna1641_F
Thermothelomyces_thermophila_ATCC_42464_rna3947_T
Thielavia_terrestris_NRRL_8126_rna1299_F
Neurospora_crassa_OR74A_rna8445_T
Madurella_mycetomatis_rna9228_T
Ophiostoma_piceae_UAMH_11346_rna5573_F
Colletotrichum_higginsianum_IMI_349063_rna8895_T
Magnaporthe_oryzae_70_15_rna11236_T
Gaeumannomyces_tritici_R3_111a_1_rna2740_T
Valsa_mali_rna9883_F
Botrytis_cinerea_B0510_rna11786_F
Amorphotheca_resinae_ATCC_22711_rna4709_F
Cadophora_sp_DSE1049_rna641_F
Erysiphe_necator_rna4392_F
Mycosphaerella_eumusae_rna7824_F
Ascosphaera_apis_ARSEF_7405_rna6276_F
Arthrobotrys_oligospora_ATCC_24927_rna3675_F
Schizosaccharomyces_pombe_rna4408_F
Bifiguratus_adelaidae_rna697_F
Ganoderma_sinense_ZZ0214_1_rna6938_F
Fomitopsis_pinicola_FP_58527_SS1_rna6711_T
Heterobasidion_irregulare_TC_32_1_rna1339_T
Jaapia_argillacea_MUCL_33604_rna15829_T
Rhizopogon_vesiculosus_rna609_F
Rhizopogon_vinicolor_AM_OR11_026_rna8206_T
Galerina_marginata_CBS_33988_rna16413_F
Hebeloma_cylindrosporum_h7_rna3378_F
rna22308
Agaricus_bisporus_var_bisporus_H97_rna10423_T
Pyrrhoderma_noxium_rna1641_F
Botryobasidium_botryosum_FD_172_SS1_rna4303_F
Calocera_viscosa_TUFC12733_rna9654_F
Cryptococcus_gattii_WM276_rna3390_F
Cryptococcus_neoformans_var_neoformans_JEC21_rna3391_F
Cutaneotrichosporon_oleaginosum_rna7558_F
Ustilago_maydis_521_rna3009_F
Kalmanozyma_brasiliensis_GHG001_rna4931_F
Anthracocystis_flocculosa_PF_1_rna5891_F
Acaromyces_ingoldii_rna2886_F
Ceraceosorus_guamensis_rna1628_F
Jaminaea_rosea_rna1653_F
Melampsora_larici_populina_98AG31_rna4013_F
Amphiamblys_sp_WSBS2006_rna1182_F
Paramicrosporidium_saccamoebae_rna825_F
Saccharomyces_cerevisiae_S288C_rna1627_F
Saccharomyces_sp_boulardii_rna5237_F
Saccharomyces_arboricola_H_6_rna848_F
Saccharomyces_eubayanus_rna1416_F
Zygosaccharomyces_parabailii_rna2955_F
Zygosaccharomyces_rouxii_rna2282_F
Torulaspora_delbrueckii_rna4768_F
Kazachstania_africana_CBS_2517_rna951_F
Naumovozyma_castellii_CBS_4309_rna1561_F
Tetrapisispora_phaffii_CBS_4417_rna3839_F
Saccharomycetaceae_sp_Ashbya_aceri_rna849_F
Eremothecium_gossypii_ATCC_10895_rna821_F
Eremothecium_cymbalariae_DBVPG_7215_rna3900_F
Kluyveromyces_lactis_rna2526_F
Hanseniaspora_osmophila_rna299_F
Hanseniaspora_uvarum_rna3380_F
Debaryomyces_hansenii_CBS767_rna5462_F
Candida_orthopsilosis_Co_90_125_rna2927_F
Metschnikowia_bicuspidata_var_bicuspidata_NRRL_YB_4993_rna4996_F
Meyerozyma_guilliermondii_ATCC_6260_rna5479_F
Babjeviella_inositovora_NRRL_Y_12698_rna2163_F
Pichia_membranifaciens_NRRL_Y_2026_rna3708_F
Pichia_kudriavzevii_rna308_F
Ogataea_parapolymorpha_DL_1_rna5010_F
Komagataella_pastoris_rna3701_F
Yarrowia_lipolytica_CLIB122_rna376_F
Sugiyamaella_lignohabitans_rna2462_F
Malassezia_restricta_rna2525_F
Malassezia_pachydermatis_rna2855_F
Lachancea_thermotolerans_CBS_6340_rna844_F
Tetrapisispora_blattae_CBS_6284_rna2219_F
Kazachstania_naganishii_CBS_8797_rna5465_F
Naumovozyma_dairenensis_CBS_421_rna4251_F
Kluyveromyces_marxianus_DMKU3_1042_rna3865_F
Cyberlindnera_jadinii_NRRL_Y_1542_rna4588_F
Candida_albicans_SC5314_rna4381_F
Candida_dubliniensis_CD36_rna5134_F
Lodderomyces_elongisporus_NRRL_YB_4239_rna1927_F
Scheffersomyces_stipitis_CBS_6054_rna2285_F
Komagataella_phaffii_GS115_rna396_F
Allomyces_macrogynus_ATCC_38327_rna16088_F
Zancudomyces_culisetae_rna7798_F
Batrachochytrium_salamandrivorans_rna2873_F
Alternaria_alternata_rna3418_F
Ascochyta_rabiei_rna3127_F
Parastagonospora_nodorum_SN15_rna5994_F
Bipolaris_oryzae_ATCC_44560_rna1497_F
Diplodia_seriata_rna5573_T
Diplodia_corticola_rna8266_F
Zymoseptoria_tritici_IPO323_rna788_F
Cercospora_beticola_rna321_F
Drechmeria_coniospora_rna2596_F
Acremonium_chrysogenum_ATCC_11550_rna613_F
Cordyceps_sp_RAO_2017_rna1251_F
Histoplasma_capsulatum_NAm1_rna7904_F
Blastomyces_dermatitidis_ER_3_rna4911_F
Capronia_coronata_CBS_61796_rna6788_F
Exophiala_dermatitidis_NIH_UT8656_rna1855_F
Exophiala_spinifera_rna7143_F
Exophiala_mesophila_rna1024_F
Aspergillus_candidus_rna7778_F
Penicillium_chrysogenum_rna11106_F
Aspergillus_calidoustus_rna6867_F
Aspergillus_aculeatus_ATCC_16872_rna529_F
Aspergillus_aculeatinus_CBS_121060_rna10946_F
Aspergillus_japonicus_CBS_114_51_rna7242_F
Aspergillus_brasiliensis_CBS_101740_rna4944_F
Aspergillus_niger_rna9941_F
Aspergillus_oryzae_RIB40_rna6326_F
Aspergillus_fumigatus_Af293_rna1176_F
0.3
