# Supplementary material for: Animal, Fungi, and Plant Genome Sequences Harbor Different Non-Canonical Splice Sites
Source: Cells. 2020 Feb 18;9(2):458. doi: 10.3390/cells9020458 (PMC7072748; doi:10.3390/cells9020458)
Supplement: Supplementary file 1 [file cells-09-00458-s001.zip › supplements/File S19.zip!/files_for_S19/25483.pdf]

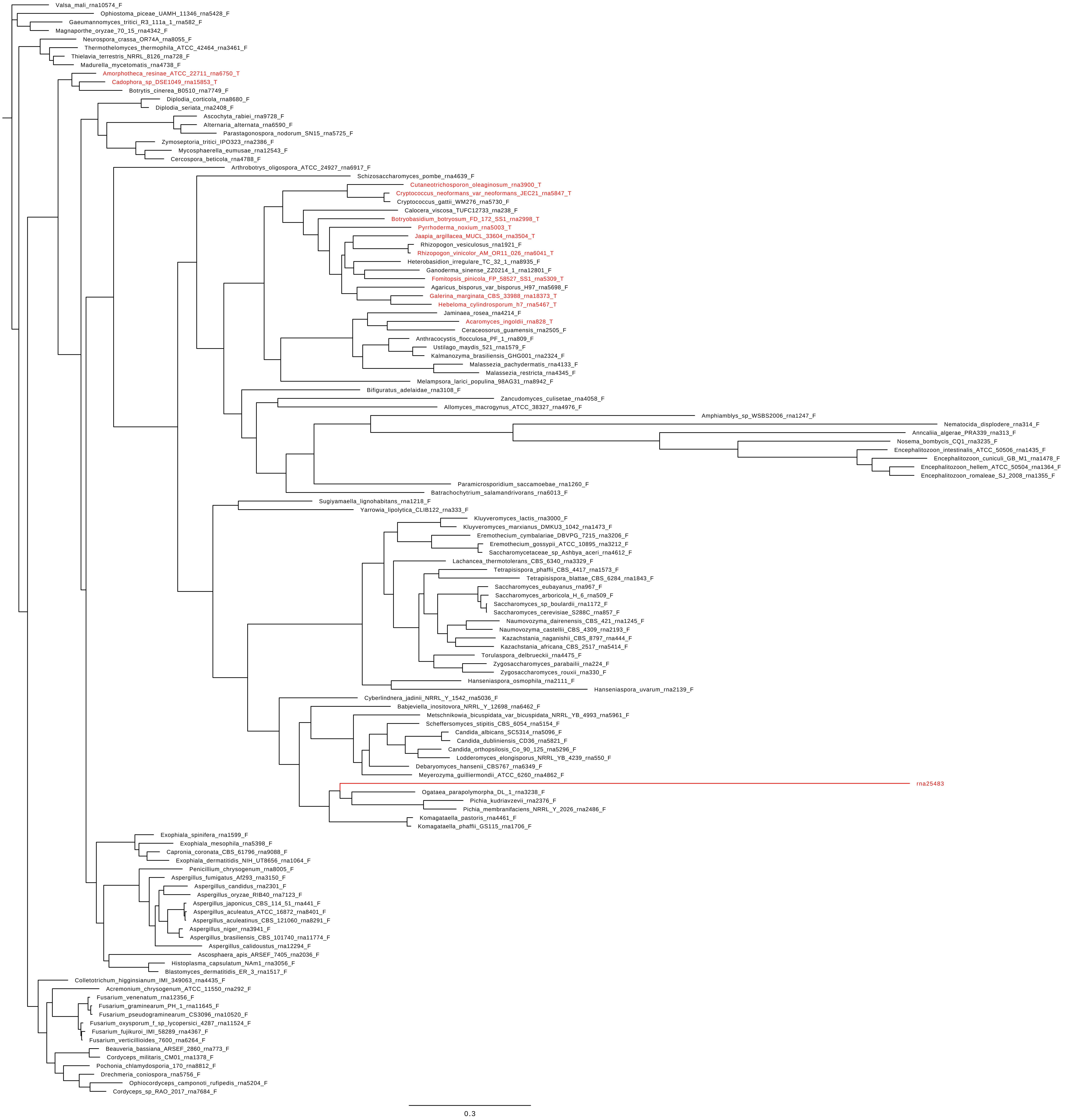
Valsa_mali_rna10574_F
Ophiostoma_piceae_UAMH_11346_rna5428_F
Gaeumannomyces_tritici_R3_111a_1_rna582_F
Magnaporthe_oryzae_70_15_rna4342_F
Neurospora_crassa_OR74A_rna8055_F
Thermothelomyces_thermophila_ATCC_42464_rna3461_F
Thielavia_terrestris_NRRL_8126_rna728_F
Madurella_mycetomatis_rna4738_F
Amorphotheca_resinae_ATCC_22711_rna6750_T
Cadophora_sp_DSE1049_rna15853_T
Botrytis_cinerea_B0510_rna7749_F
Diplodia_corticola_rna8680_F
Diplodia_seriata_rna2408_F
Ascochyta_rabiei_rna9728_F
Alternaria_alternata_rna6590_F
Parastagonospora_nodorum_SN15_rna5725_F
Zymoseptoria_tritici_IPO323_rna2386_F
Mycosphaerella_eumusae_rna12543_F
Cercospora_beticola_rna4788_F
Arthrobotrys_oligospora_ATCC_24927_rna6917_F
Schizosaccharomyces_pombe_rna4639_F
Cutaneotrichosporon_oleaginosum_rna3900_T
Cryptococcus_neoformans_var_neoformans_JEC21_rna5847_T
Cryptococcus_gattii_WM276_rna5730_F
Calocera_viscosa_TUFC12733_rna238_F
Botryobasidium_botryosum_FD_172_SS1_rna2998_T
Pyrrhoderma_noxium_rna5003_T
Jaapia_argillacea_MUCL_33604_rna3504_T
Rhizopogon_vesiculosus_rna1921_F
Rhizopogon_vinicolor_AM_OR11_026_rna6041_T
Heterobasidion_irregulare_TC_32_1_rna8935_F
Ganoderma_sinense_ZZ0214_1_rna12801_F
Fomitopsis_pinicola_FP_58527_SS1_rna5309_T
Agaricus_bisporus_var_bisporus_H97_rna5698_F
Galerina_marginata_CBS_33988_rna18373_T
Hebeloma_cylindrosporum_h7_rna5467_T
Jaminaea_rosea_rna4214_F
Acaromyces_ingoldii_rna828_T
Ceraceosorus_guamensis_rna2505_F
Anthracocystis_flocculosa_PF_1_rna809_F
Ustilago_maydis_521_rna1579_F
Kalmanozyma_brasiliensis_GHG001_rna2324_F
Malassezia_pachydermatis_rna4133_F
Malassezia_restricta_rna4345_F
Melampsora_larici_populina_98AG31_rna8942_F
Bifiguratus_adelaidae_rna3108_F
Zancudomyces_culisetae_rna4058_F
Allomyces_macrogynus_ATCC_38327_rna4976_F
Amphiamblys_sp_WSBS2006_rna1247_F
Nematocida_displodere_rna314_F
Anncaliia_algerae_PRA339_rna313_F
Nosema_bombycis_CQ1_rna3235_F
Encephalitozoon_intestinalis_ATCC_50506_rna1435_F
Encephalitozoon_cuniculi_GB_M1_rna1478_F
Encephalitozoon_hellem_ATCC_50504_rna1364_F
Encephalitozoon_romaleae_SJ_2008_rna1355_F
Paramicrosporidium_saccamoebae_rna1260_F
Batrachochytrium_salamandrivorans_rna6013_F
Sugiyamaella_lignohabitans_rna1218_F
Yarrowia_lipolytica_CLIB122_rna333_F
Kluyveromyces_lactis_rna3000_F
Kluyveromyces_marxianus_DMKU3_1042_rna1473_F
Eremothecium_cymbalariae_DBVPG_7215_rna3206_F
Eremothecium_gossypii_ATCC_10895_rna3212_F
Saccharomycetaceae_sp_Ashbya_aceri_rna4612_F
Lachancea_thermotolerans_CBS_6340_rna3329_F
Tetrapisispora_phaffii_CBS_4417_rna1573_F
Tetrapisispora_blattae_CBS_6284_rna1843_F
Saccharomyces_eubayanus_rna967_F
Saccharomyces_arboricola_H_6_rna509_F
Saccharomyces_sp_boulardii_rna1172_F
Saccharomyces_cerevisiae_S288C_rna857_F
Naumovozyma_dairenensis_CBS_421_rna1245_F
Naumovozyma_castellii_CBS_4309_rna2193_F
Kazachstania_naganishii_CBS_8797_rna444_F
Kazachstania_africana_CBS_2517_rna5414_F
Torulaspora_delbrueckii_rna4475_F
Zygosaccharomyces_parabailii_rna224_F
Zygosaccharomyces_rouxii_rna330_F
Hanseniaspora_osmophila_rna2111_F
Hanseniaspora_uvarum_rna2139_F
Cyberlindnera_jadinii_NRRL_Y_1542_rna5036_F
Babjeviella_inositovora_NRRL_Y_12698_rna6462_F
Metschnikowia_bicuspidata_var_bicuspidata_NRRL_YB_4993_rna5961_F
Scheffersomyces_stipitis_CBS_6054_rna5154_F
Candida_albicans_SC5314_rna5096_F
Candida_dubliniensis_CD36_rna5821_F
Candida_orthopsilosis_Co_90_125_rna5296_F
Lodderomyces_elongisporus_NRRL_YB_4239_rna550_F
Debaryomyces_hansenii_CBS767_rna6349_F
Meyerozyma_guilliermondii_ATCC_6260_rna4862_F
rna25483
Ogataea_parapolymorpha_DL_1_rna3238_F
Pichia_kudriavzevii_rna2376_F
Pichia_membranifaciens_NRRL_Y_2026_rna2486_F
Komagataella_pastoris_rna4461_F
Komagataella_phaffii_GS115_rna1706_F
Exophiala_spinifera_rna1599_F
Exophiala_mesophila_rna5398_F
Capronia_coronata_CBS_61796_rna9088_F
Exophiala_dermatitidis_NIH_UT8656_rna1064_F
Penicillium_chrysogenum_rna8005_F
Aspergillus_fumigatus_Af293_rna3150_F
Aspergillus_candidus_rna2301_F
Aspergillus_oryzae_RIB40_rna7123_F
Aspergillus_japonicus_CBS_114_51_rna441_F
Aspergillus_aculeatus_ATCC_16872_rna8401_F
Aspergillus_aculeatinus_CBS_121060_rna8291_F
Aspergillus_niger_rna3941_F
Aspergillus_brasiliensis_CBS_101740_rna11774_F
Aspergillus_calidoustus_rna12294_F
Ascosphaera_apis_ARSEF_7405_rna2036_F
Histoplasma_capsulatum_NAm1_rna3056_F
Blastomyces_dermatitidis_ER_3_rna1517_F
Colletotrichum_higginsianum_IMI_349063_rna4435_F
Acremonium_chrysogenum_ATCC_11550_rna292_F
Fusarium_venenatum_rna12356_F
Fusarium_graminearum_PH_1_rna11645_F
Fusarium_pseudograminearum_CS3096_rna10520_F
Fusarium_oxysporum_f_sp_lycopersici_4287_rna11524_F
Fusarium_fujikuroi_IMI_58289_rna4367_F
Fusarium_verticillioides_7600_rna6264_F
Beauveria_bassiana_ARSEF_2860_rna773_F
Cordyceps_militaris_CM01_rna1378_F
Pochonia_chlamydosporia_170_rna8812_F
Drechmeria_coniospora_rna5756_F
Ophiocordyceps_camponoti_rufipedis_rna5204_F
Cordyceps_sp_RAO_2017_rna7684_F
0.3
